# Recent advances in the synthesis and synthetic applications of Betti base (aminoalkylnaphthol) and bis-Betti base derivatives

**DOI:** 10.1039/c9ra02813g

**Published:** 2019-06-11

**Authors:** Abolfazl Olyaei, Mahdieh Sadeghpour

**Affiliations:** Department of Chemistry, Payame Noor University (PNU) PO Box 19395-4697 Tehran Iran olyaei_a@pnu.ac.ir; Department of Chemistry, Takestan Branch, Islamic Azad University Takestan Iran

## Abstract

The multicomponent reaction between 2-naphthol, arylaldehydes and ammonia yields aminobenzylnaphthols in a process known as the Betti reaction, which was first uncovered at the beginning of the 20th century. Various methods have been reported for the synthesis of aminobenzylnaphthol (Betti base) and bis-Betti base derivatives using various types of naphthols, aromatic amines, heteroaromatic amines, and aliphatic and cyclic amines instead of ammonia or diamines and aliphatic and aromatic aldehydes or dialdehyde compounds under various conditions in recent years. The Betti reaction produces racemic and non-racemic aminobenzylnaphthol ligands. It is also clear that the most important area of application of the non-racemic aminonaphthols prepared in this manner is their use in asymmetric synthesis, either as chiral ligands or as chiral auxiliaries. The functional groups in these Mannich products offer many ring closure possibilities. Some of these products or the starting bifunctional compounds possess biological activity. Herein, we present a selection of the relevant studies on this topic.

## Abstract 2

The multicomponent reaction between 2-naphthol, arylaldehydes and ammonia yields aminobenzylnaphthols in a process known as the Betti reaction, which was first uncovered at the beginning of the 20th century.

## Graphical abstract

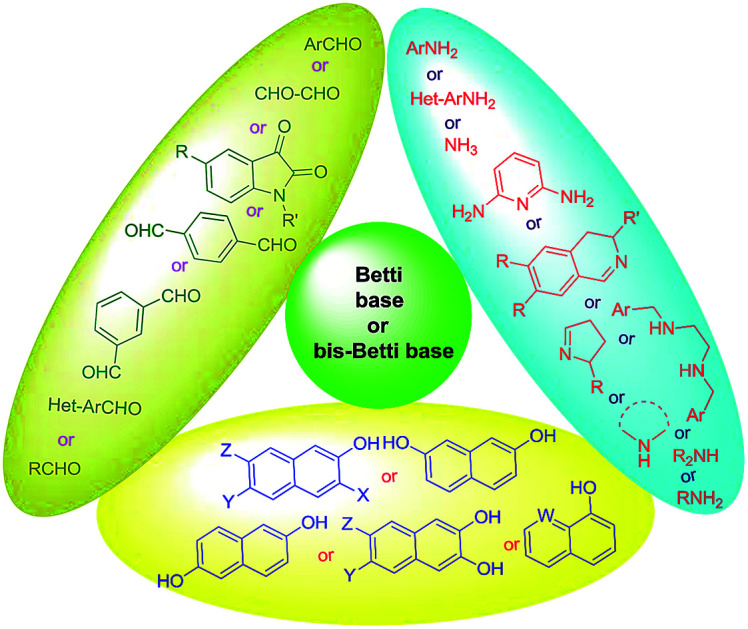

## 1. Introduction

**Fig. 1:**
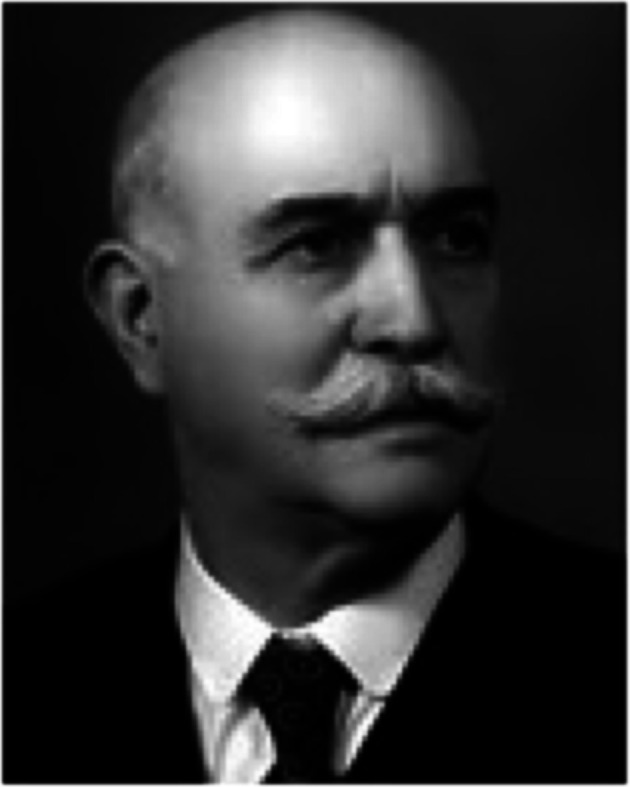
Italian Senator Mario Betti.

Senator Mario Betti (1875–1942) (Fig. 1) was a distinguished Italian chemist,^1,2^ very active at the beginning of the 20th century. In 1892 Betti registered as a student at the chemistry school of the University of Pisa. In 1897, Betti obtained his degree with a thesis on the reaction of methylisoxazolones with aldehydes and co-authored with Roberto Schiff the first two papers of his career.^3,4^ In 1898, Betti moved to the University of Florence as an assistant of Ugo Schiff, who was founder and director of the Institute of Chemistry. In the Florence laboratories, Betti developed his interests in stereochemistry with important studies on the relationship between optical rotatory power and the structure of groups connected with the stereocentre.^5^ Noyori considered him to be the real pioneer of asymmetric synthesis,^6^ since Betti reacted methylmagnesium iodide and benzaldehyde in the presence of *N*,*N*-dimethylbornylamine.

**Fig. 2:**
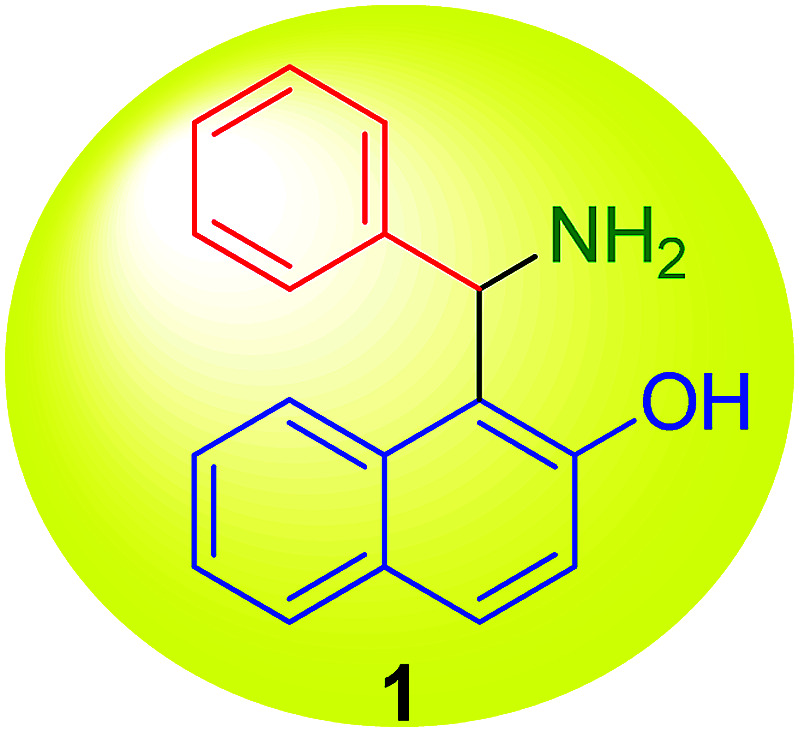
The Betti base structure.

On performing these studies, a great advantage was achieved by the synthetic versatility of the reaction, nowadays known as the Betti reaction, that in the original version produced 1-(α-aminobenzyl)-2-naphthol (1) (Fig. 2), starting with 2-naphthol, benzaldehyde, and ammonia.^7^ The product began to be known by the name of the author (*i.e.* Betti base) and the multi-component process was published in *Organic Syntheses*,^8^ a prestigious international book series, where only independently checked synthetic procedures are reported. Finally, the base was also easily resolved into its optical isomers by means of tartaric acid.^9^

Also, he worked at the universities of Cagliari, Siena, Genoa and finally Bologna, where he was the successor of Giacomo Ciamician. In 1939, he was appointed a Senator of the Kingdom of Italy.

The purpose of the present review is to summarize the utility of the Betti bases and bis-Betti bases with emphasis on recent synthesis and synthetic applications.

## 2. The Betti reaction

**Scheme 1:**
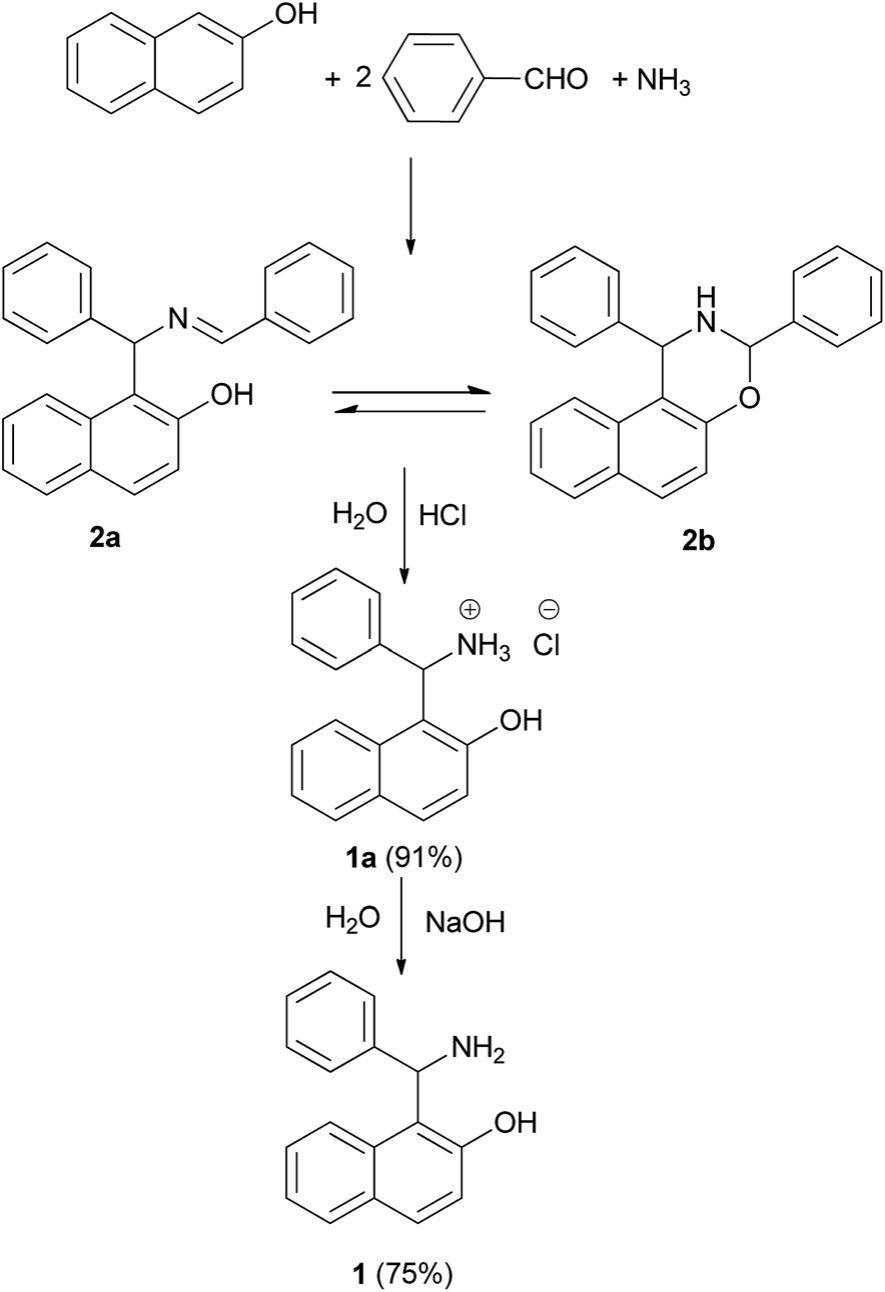
The Betti reaction.

This synthetic strategy originated between the end of the 19th and the beginning of the 20th century when research in several laboratories was performed on reactions between ammonia, or amines, formaldehyde and enolisable carbonyl compounds.^10^ The first two components yield an imine that reacts with the carbonyl compound. These procedures are commonly classified as Mannich aminoalkylations, after the systematic work of the latter author, which began in 1912, thus subsequent to Betti's research.^7,9,11^ Eventually, Betti also reported^7^ that the product 2 (Scheme 1) could be obtained from a three-component condensation of 2-naphthol, an ethanolic solution of ammonia and 2 equiv. of benzaldehyde (91% yield). Actually, the product of the reaction is represented by the forms 2a and 2b in equilibrium.^12^ The intermediate 2 was treated with hydrochloric acid to obtain the salt of the Betti base 1a HCl (91% yield, Scheme 1). Addition of a solution of sodium hydroxide to chloride yielded Betti base 1 (75% yield).^7,13^

## 3. Synthesis and synthetic applications of Betti base derivatives

**Scheme 2:**
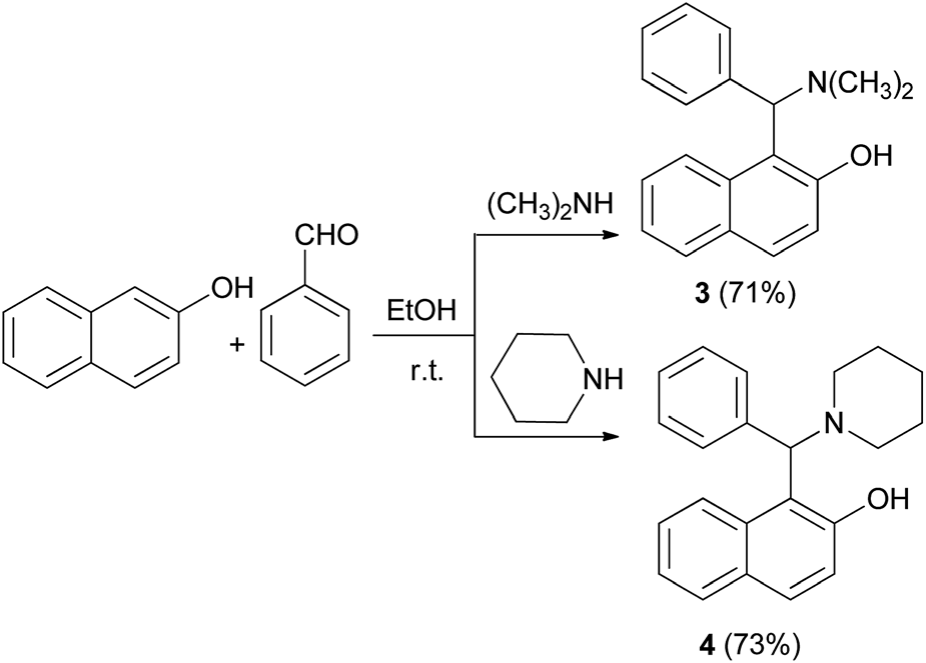
The Betti reaction with secondary amines.

In 1930, after a long period of silence, work was performed by Littman and Brode, who used different secondary amines such as dimethylamine and piperidine instead of ammonia in a one-pot multicomponent process.^14^ In this reaction, dimethylamino derivative of the Betti base 3 and 1-(1-piperidylbenzyl)-2-naphthol (4) were obtained (Scheme 2).^14^ According to Littman and Brode, secondary amines should react with benzaldehyde *via* the formation of a benzylidinediamine. This intermediate attacks 2-naphthol and yields aminobenzylnaphthol, after the elimination of an amine molecule.

**Scheme 3:**
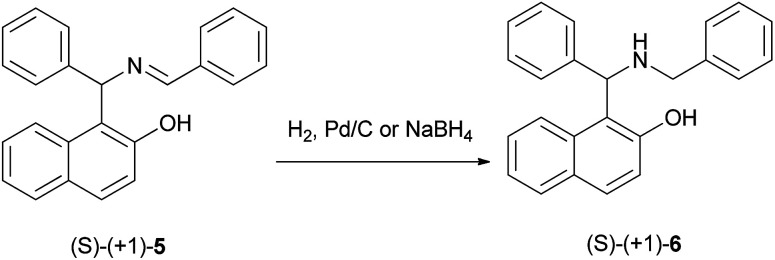
Synthesis of the *N*-benzyl derivative 6.

Two decades ago, Cardellicchio *et al.*^15^ reported a protocol for the resolution of the Betti base and the absolute configuration of the Betti base hydrobromide was established by means of X-ray diffractometry. Also, a series of optically active derivatives such as the *N*,*N*-dimethyl and the *N*,*N*,*O*-trimethyl derivatives were prepared in racemic form by means of the Betti reaction and were resolved into two enantiomers with an extremely easy and efficient procedure. The configuration of each base was determined by correlation with the configuration of the Betti amine. In their work, the *N*-benzyl derivative 6 was easily obtained by reducing (*S*)-(+)-5 with hydrogen in the presence of Pd/C, or with NaBH_4_ (Scheme 3).

**Scheme 4:**
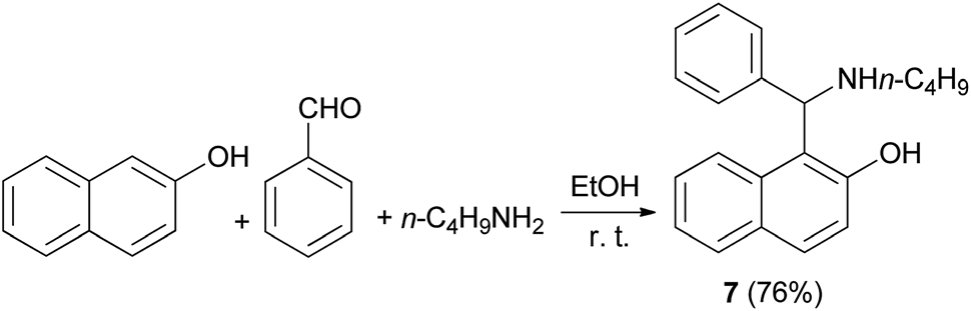
Synthesis of the *N*-butyl derivative 7.

In 1999, Naso and coworkers reported a procedure for the synthesis of l-(α-*N*-butylaminobenzyl)-2-naphthol (7) *via* condensation of 2-naphthol, benzaldehyde and *n*-butylamine in EtOH (95%) at room temperature for 6 days, obtaining the corresponding product in 76% yield (Scheme 4).

**Scheme 5:**
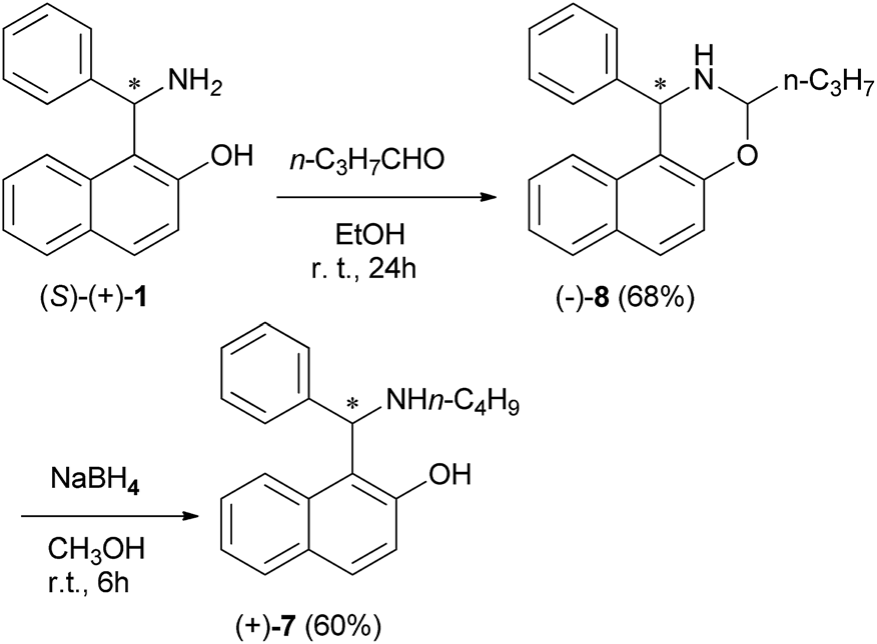
Synthesis of chiral aminonaphthol (+)-7.

Also, in order to establish the configuration of product 7, the Betti base (*S*)-(+)-1 was treated with *n*-butanal, yielding the oxazine (−)-8, which was reduced to (+)-7 by NaBH_4_; thus the (*S*) configuration has been assigned to compound (+)-7 and consequently the (*R*) configuration to (−)-7 (Scheme 5). The chiral non-racemic aminonaphthols 7 were tested as complexing agents in the catalytic enantioselective addition of diethylzinc to arylaldehydes. The use of these bases gave high ee values (up to >99%). An important role was played by the solvent. Toluene or hexane gave the best results and THF lowered the ee values of the resulting alcohol.^16^

**Scheme 6:**
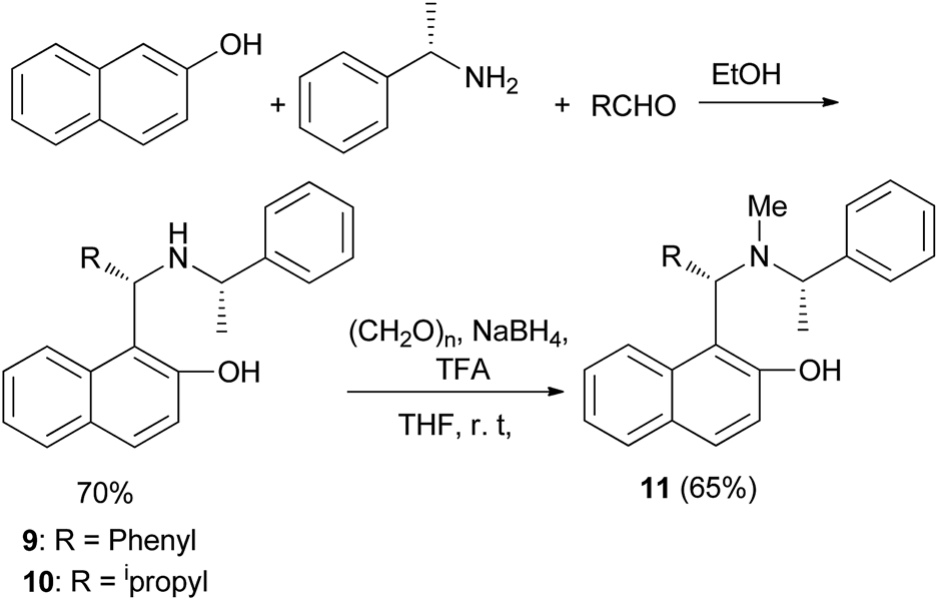
Synthesis of chiral aminonaphthol 11.

Wang *et al.*^17^ have obtained optically active aminonaphthols 9 and 10 through the reaction of 2-naphthol, benzaldehyde, and (*S*)-methylbenzylamine in EtOH at room temperature for 6 days in 70% yield. Moreover, *N*-methylation of 9 with paraformaldehyde and NaBH_4_ in THF at room temperature gave 1-((*S*)-phenyl(((1′*S*)-1′-phenylethyl)methylamino)methyl)-2-naphthol (11) in 65% yield (Scheme 6). Aminonaphthol 11 (15 mol%) catalyzed the enantioselective ethylation of arylaldehydes to secondary alcohols (70–98% yields) with high enantioselectivities (up to 99.8%) in toluene at room temperature for 24 h.

**Scheme 7:**
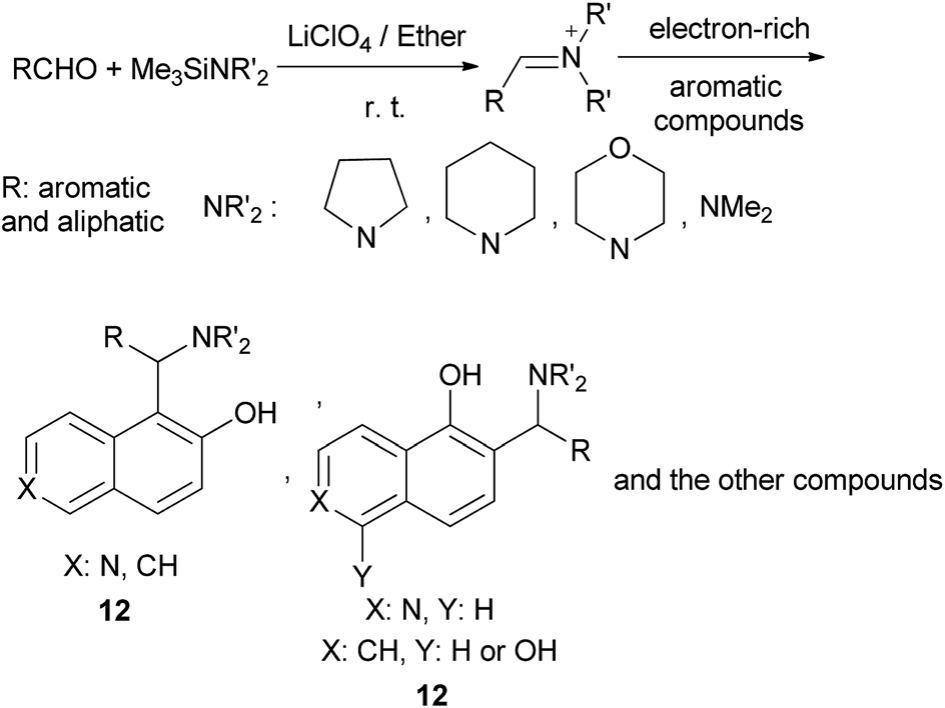
LiClO_4_-mediated aminoalkylation of electron-rich aromatic compounds.

Saidi *et al.*^18^ described an efficient three-component and one-pot method for aminoalkylation of electron-rich aromatic compounds using aldehydes, (trimethylsilyl)dialkylamines, and an electron-rich aromatic compound such as 1-naphthol, 2-naphthol, 1,5-dihydroxynaphthalene, 2,4-dimethylphenol, 6-hydroxyisoquinoline, 7-hydroxycoumarin, indole or *N*-methylindole, at room temperature for 1–6 h in a concentrated solution of lithium perchlorate in diethyl ether. The reaction give good yields up to 95% of aminoalkylated aromatic and aliphatic compounds 12 and with moderate yield (35%) in the case of aliphatic aldehydes (Scheme 7).

**Scheme 8:**
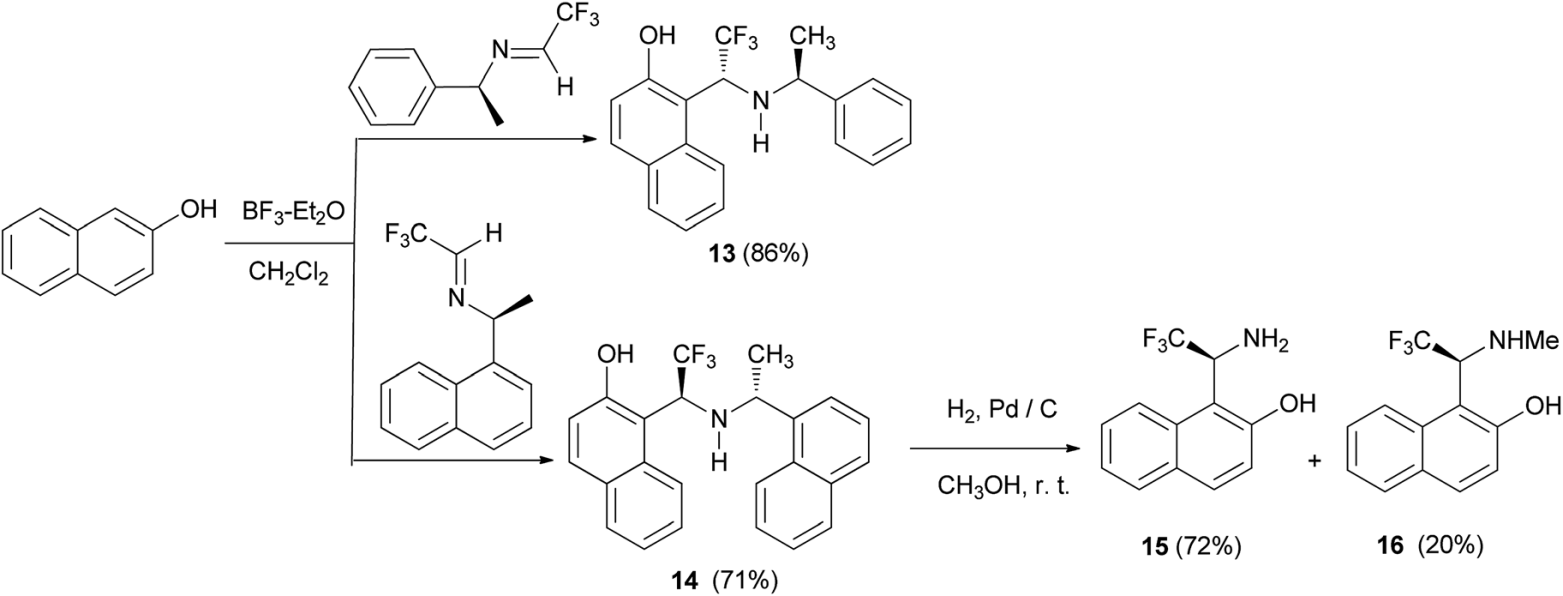
Diastereoselective synthesis of Betti bases 13–16.

Gong *et al.*^19^ demonstrated diastereoselective Friedel–Crafts reaction of 2-naphthol with α-trifluoromethylimines derived from chiral amines. The reactions proceeded readily at room temperature for 48 h in the presence of BF_3_·Et_2_O, resulting in the corresponding chiral Betti bases 13 and 14 in yields of 86% and 71%, respectively. Then, hydrogenolysis of 14 was carried out in methanol at room temperature under a hydrogen atmosphere in the presence of Pd/C. Under these conditions, compound 14 gave 1-(1-amino-2,2,2-trifluoro)ethylnaphthalen-2-ol (15) (72%) and 1-(2,2,2-trifluoro-1-methylamino)ethylnaphthalen-2-ol (16) (20%) (Scheme 8).

**Scheme 9:**
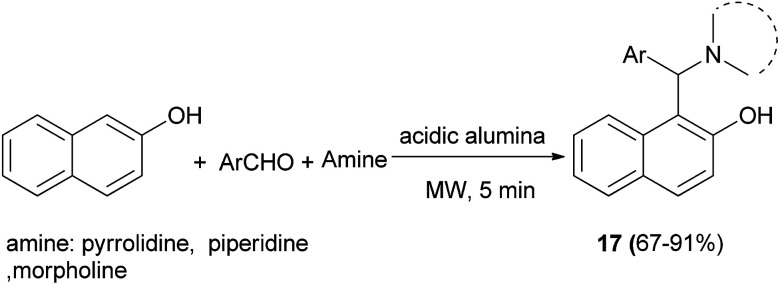
Aminoalkylation of 2-naphthol on acidic alumina promoted by microwave irradiation.

The Mannich reaction on acidic alumina assisted by microwave irradiation was reported for the aminoalkylation of 2-naphthol with aromatic aldehydes and secondary amines such as piperidine, pyrrolidine and morpholine for 5 min under solvent-free condition, resulting in the desired products 17 in 67–91% yields (Scheme 9).^20^

**Scheme 10:**
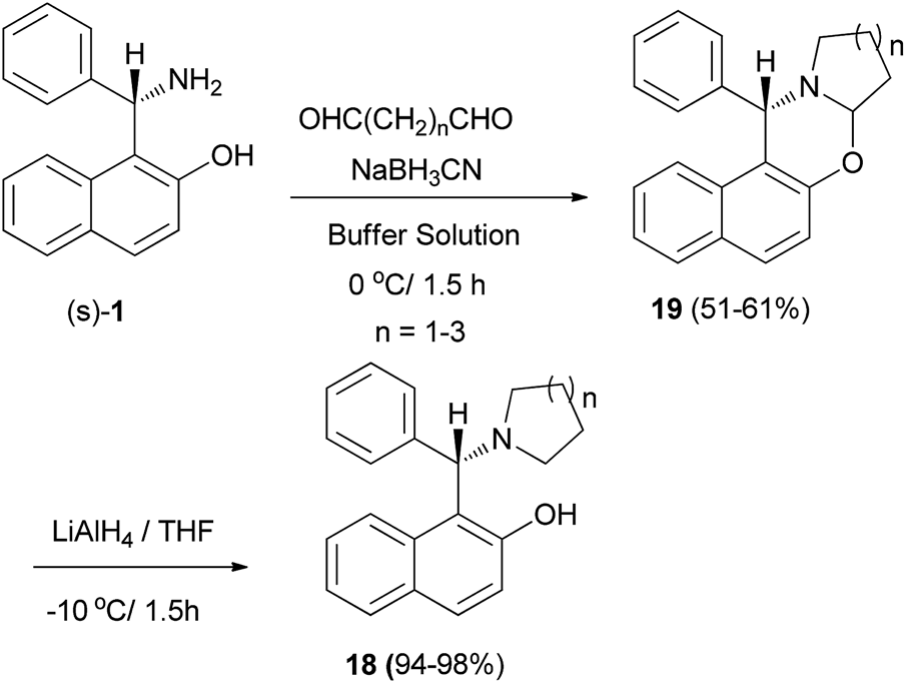
Synthesis of chiral ligands (*S*)-18.

A procedure for selective direct *N*,*N*-alkylation of the chiral Betti base was developed, and a family of chiral ligands, (*S*)-1-(α-cycloaminobenzyl)-2-naphthols 18, were prepared. Initially, oxazine derivatives 19 were achieved by the condensation of (*S*)-1-(α-aminobenzyl)-2-naphthol and bis-aldehydes in the presence of NaBH_3_CN in a buffer solution (aqueous EtOH solution of Na_2_HPO_4_–KH_2_PO_4_) at 0 °C in 1.5 h. Subsequently, compounds 19 were treated with LiAlH_4_, the C–O bond being cleaved selectively to yield the desired products 18 in 94–98% yields at −10 °C in 1.5 h without any loss of enantiomeric excess (Scheme 10).^21^

**Scheme 11:**
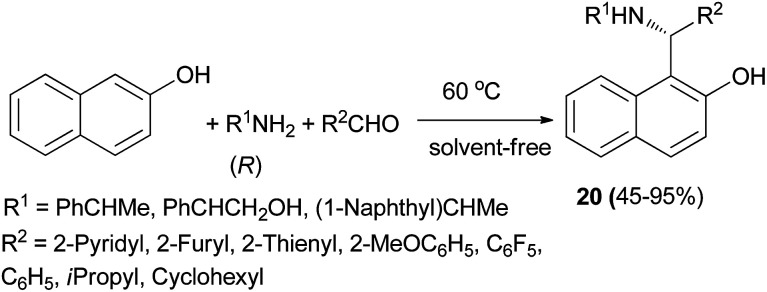
Synthesis of secondary aminoalkylnaphthols 20.

**Scheme 12:**
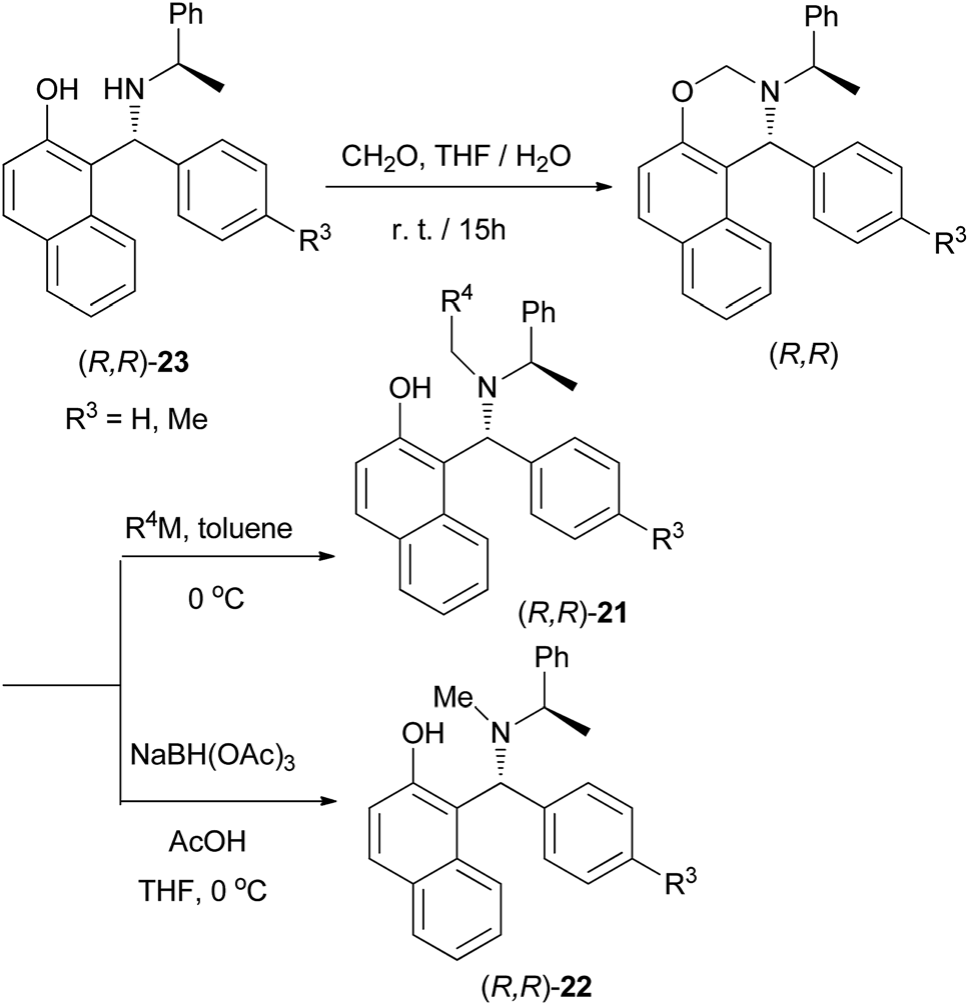
Synthesis of tertiary aminoalkylnaphthols 21 and 22.

A practical procedure for the stereoselective synthesis of a group of functionalized secondary and tertiary aminoalkylnaphthols (20, 21 and 22) was employed by Palmieri *et al.*^22^ A series of secondary aminoalkylnaphthols were prepared by heating a mixture of 2-naphthol, (*R*)-amines and aldehydes at 60 °C in 8–30 h, under solvent-free conditions with yields from 45 to 95% (Scheme 11). Then, selective *N*-alkylation was carried out by cyclization of compounds 23 with formaldehyde, followed by reduction or alkylation with organometallic reagents (R^4^M), resulting in the corresponding chiral tertiary aminonaphthols 21 and 22 (Scheme 12). The catalytic activity of this class of compounds was used in the addition of diethylzinc to benzaldehyde in toluene at room temperature, resulting in moderate to good enantioselectivities (12–89% ee) and 26–97% yields.

**Scheme 13:**
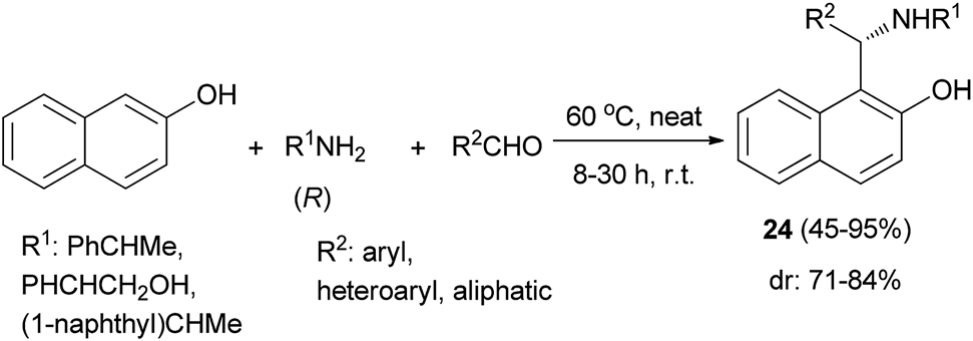
Synthesis of secondary aminoalkylnaphthols 24.

A practical procedure for the stereoselective synthesis of a group of functionalized aminoalkylnaphthols (24) in 45–95% yields was reported, involving heating a mixture of 2-naphthol, secondary amines (*R*) and aldehydes at 60 °C within 8–30 h under solvent-free conditions (Scheme 13). It is noteworthy that the aminonaphthols obtained as the major diastereomer (dr: 71–84%) in the solvent-free synthesis have the best asymmetric induction properties in the alkylation reaction.^22^

**Scheme 14:**
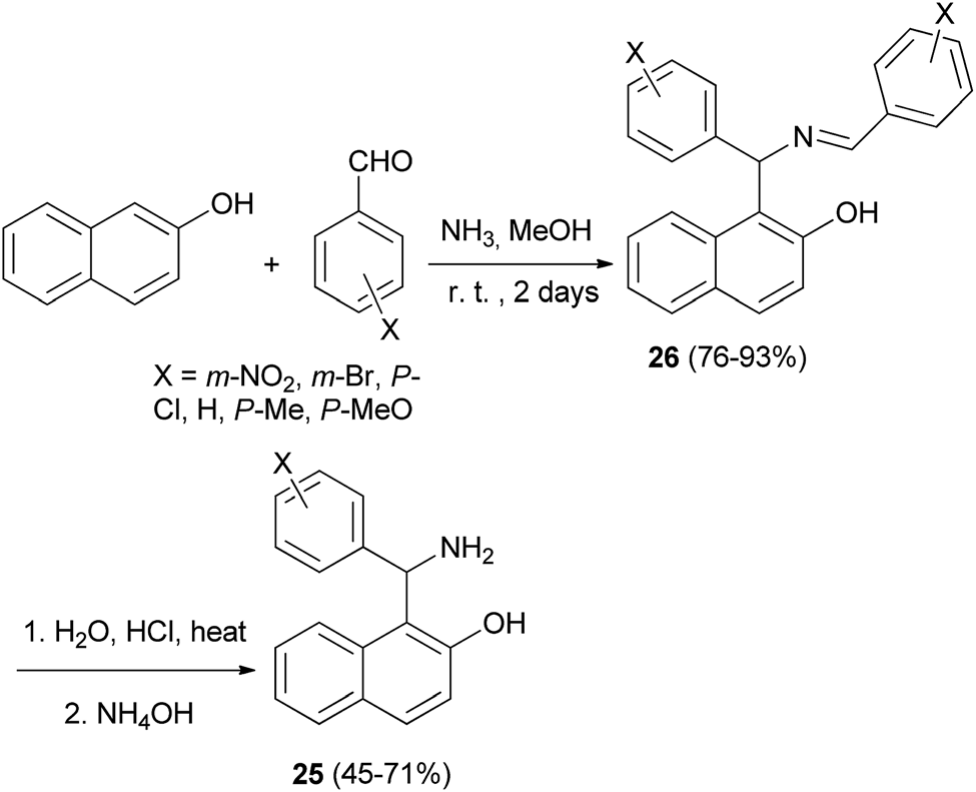
Synthesis of aminoalkylnaphthols 25.

Condensation of 2-naphthol and substituted benzaldehydes in the presence of 25% methanolic ammonia, and subsequent acidic hydrolysis afforded the aminoalkylnaphthols 25 in 45–71% yields. The crystalline product 26 separated out after 2 days, which was suspended in 20% HCl and the mixture was stirred and refluxed for 3 h (Scheme 14).^23^

**Scheme 15:**
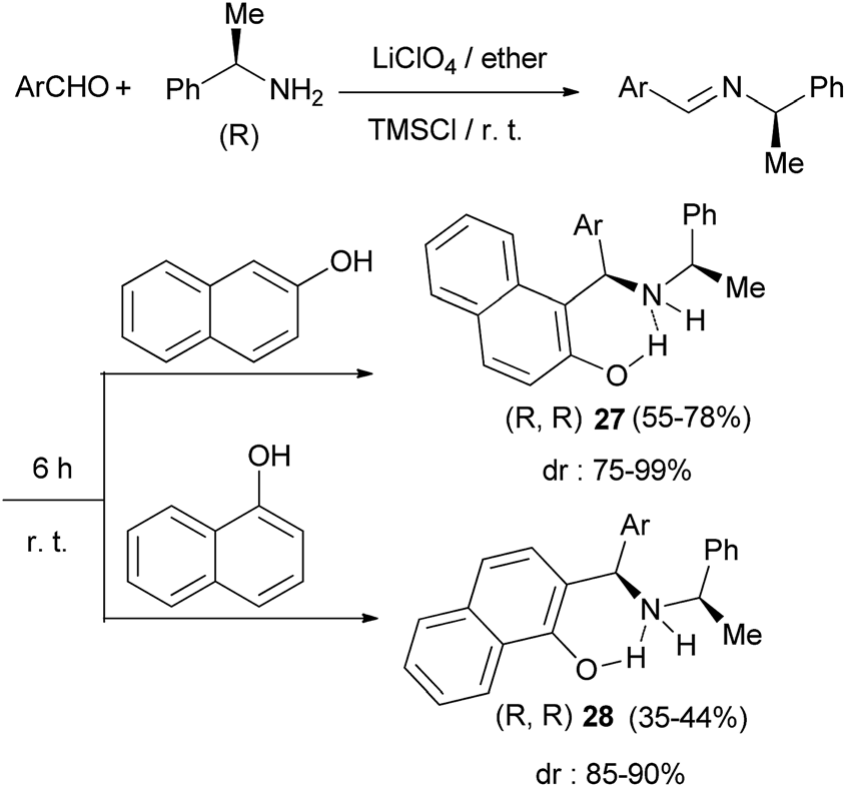
LiClO_4_-mediated stereoselective aminoalkylation of naphthols with chiral amine.

The Mannich reaction of 2-naphthol and 1-naphthol with *in situ* prepared imines from (*R*)-1-phenylethylamine and aromatic aldehydes in 5 M ethereal lithium perchlorate at room temperature in the presence of TMSCl under an argon atmosphere within 6 h afforded the corresponding aminoalkylated products 27 and 28 in moderate to good yields (35–78%) with moderate to very high diastereoselectivities (75–99%) (Scheme 15).^24^

**Scheme 16:**
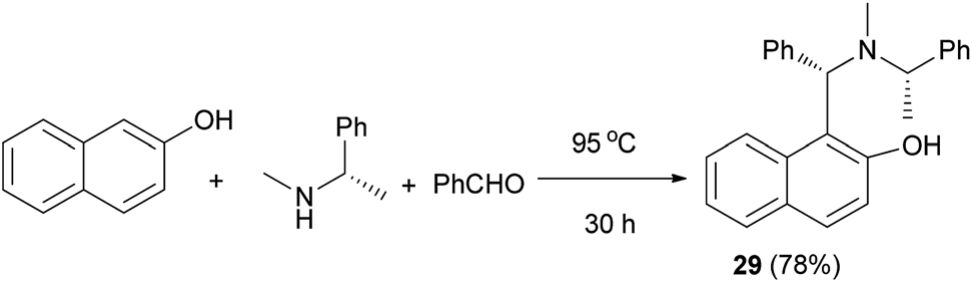
One-step synthesis of aminonaphthol 29.

Chan *et al.*^25^ reported a one-step procedure for the synthesis of optically active tertiary aminonaphthol 29*via* benzaldehyde, (*S*)-(−)-*N*,*R*-dimethylbenzylamine and 2-naphthol at 95 °C for 30 h in 78% yield (Scheme 16). Optically active tertiary aminonaphthol 29 (15 mol%) was found to catalyze the enantioselective alkenylation of various aldehydes in toluene at −30 °C for 15 h with high ee values (up to >99%), which provides a practical method for the synthesis of chiral (*E*)-allyl alcohols in 77–95% yields.

**Scheme 17:**
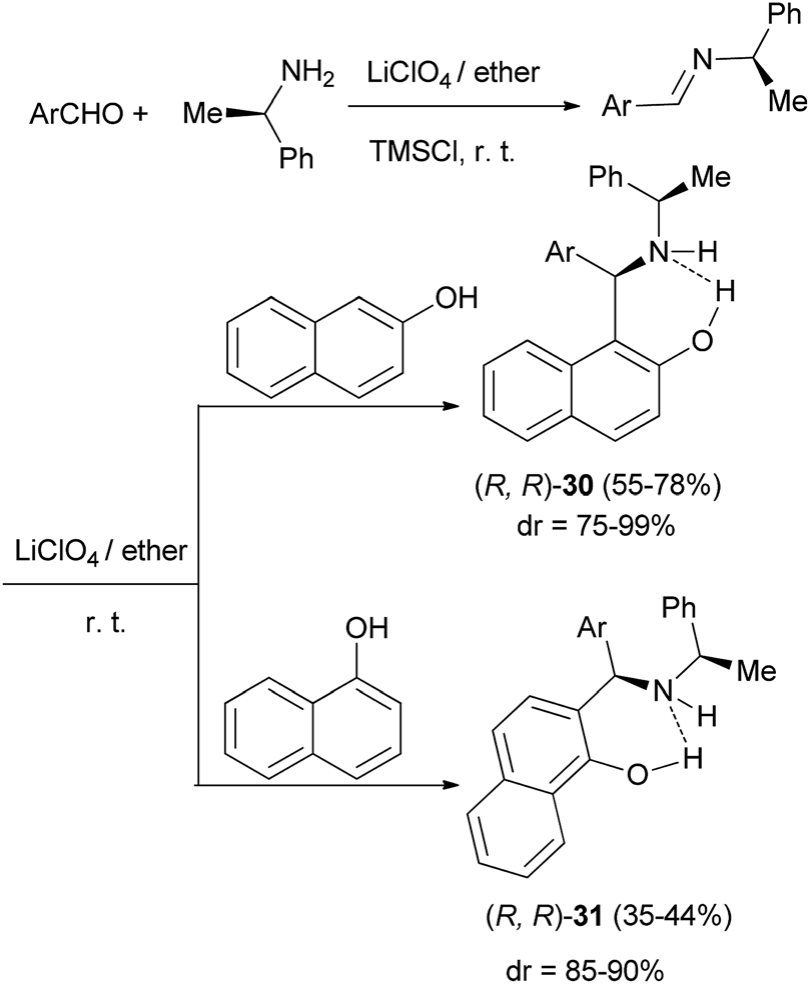
LiClO_4_-mediated stereoselective aminoalkylation of naphthols with chiral amine.

Saidi *et al.*^26^ reported a one-pot, three-component Mannich reaction of 2-naphthol or 1-naphthol with (*R*)-1-phenylethylamine and an aromatic aldehyde in concentrated ethereal lithium perchlorate solution at room temperature, which afforded highly diastereoselective access to the requisite 2-aminoalkylated products 30 and 31 in high to low yields, respectively (Scheme 17).

**Scheme 18:**
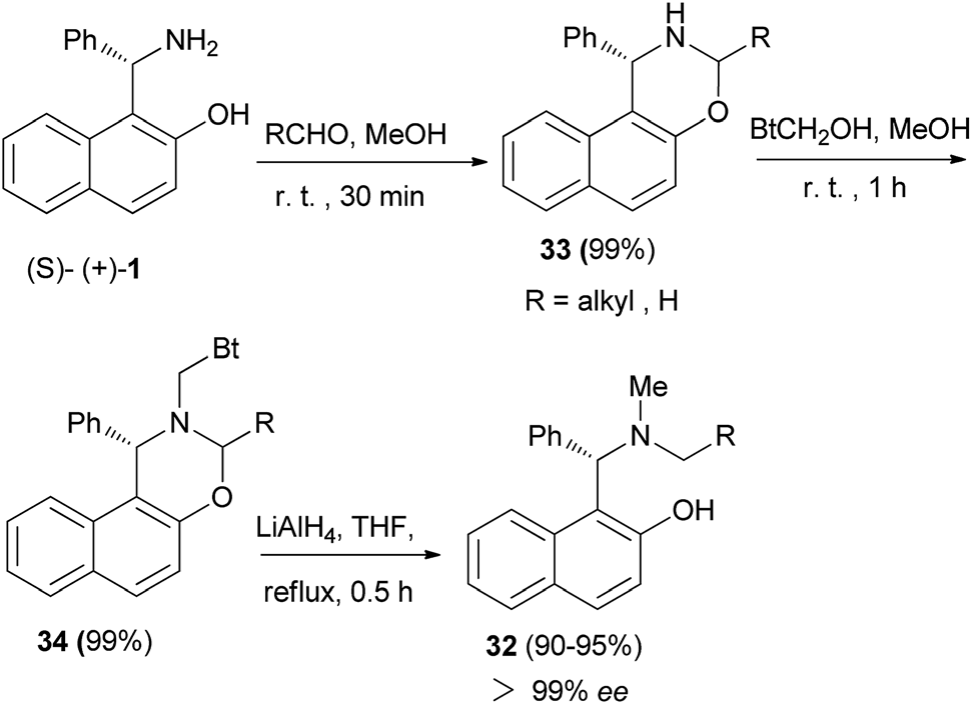
Synthesis of chiral *N*-methyl-*N*-alkyl Betti bases 32.

In 2004, a one-pot preparation of chiral *N*-methyl-*N*-alkyl Betti bases 32 was reported involving a highly regioselective *N*-alkylation of (*S*)-(+)-Betti base. The strategy involved formation of an oxazine ring (33), obtained by condensation of (*S*)-(+)-Betti base and aldehydes, and *N*-methylation with BtCH_2_OH under neutral conditions to yield oxazine 34. Chiral *N*-methyl-*N*-alkyl Betti base 32 was then obtained by simultaneously cleaving the C–Bt bond and C–O bond in the structure of 34*via* LiAlH_4_ (Scheme 18).^27^

**Scheme 19:**
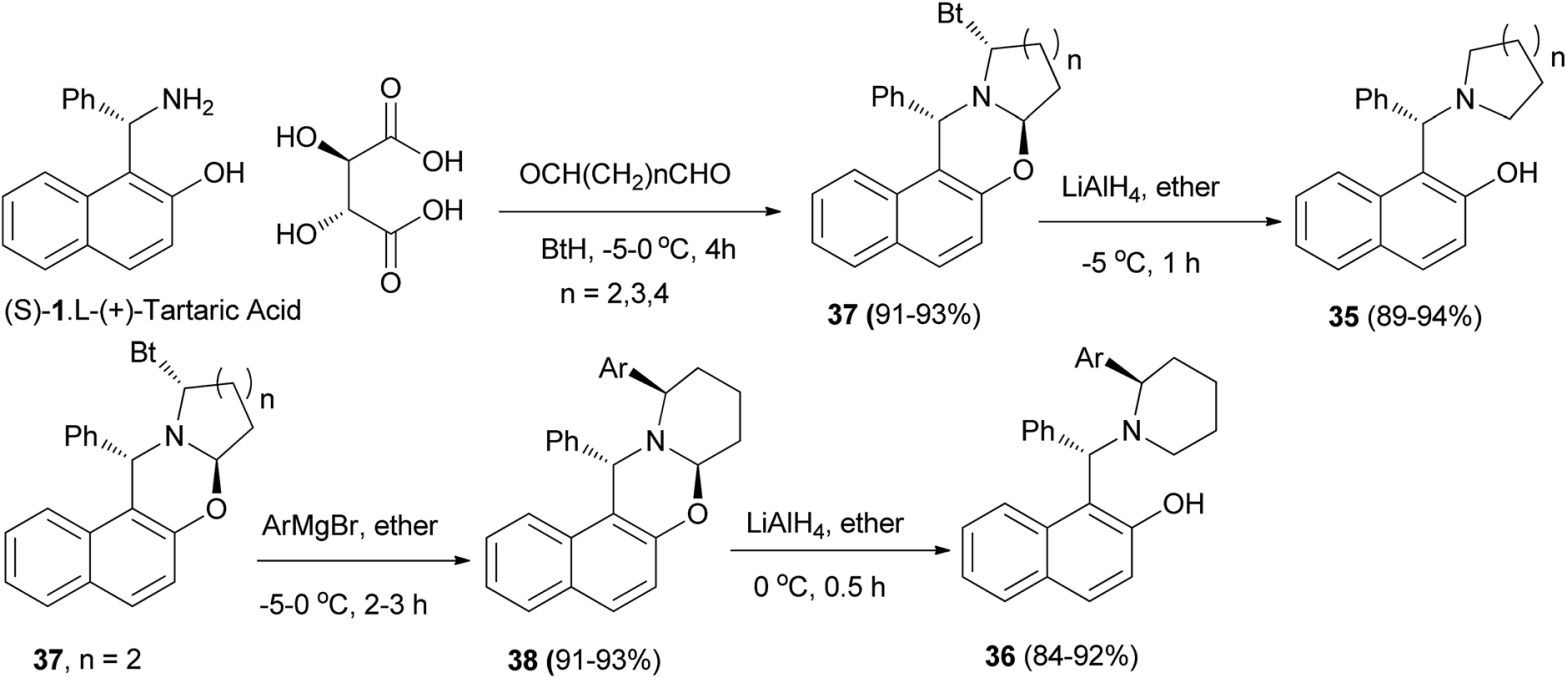
Synthesis of homochiral 1-[α-(1-azacycloalkyl)benzyl]-2-naphthols 35 and 36.

Hu *et al.*^28^ improved the synthesis of homochiral 1-[α-(1-azacycloalkyl)benzyl]-2-naphthols 35 and 1-[α-(2-arylpiperidyl)benzyl]-2-naphthols 36 by employing diastereomerically pure α-benzotriazolyl-1-azacycloalka[2,1-*b*][1,3]oxazines 37 and 38 as homochiral precursors, which were obtained by condensation between nonracemic Betti base and dialdehydes in the presence of benzotriazole. Reduction of oxazines 37 with LiAlH_4_ afforded chiral ligands 35 in yields of 89–94%. Moreover, oxazines 38 can be arylated with ArMgBr, followed by reduction with LiAlH_4_ to give a group of potential chiral ligands 36 in 84–92% yield (Scheme 19).

**Scheme 20:**
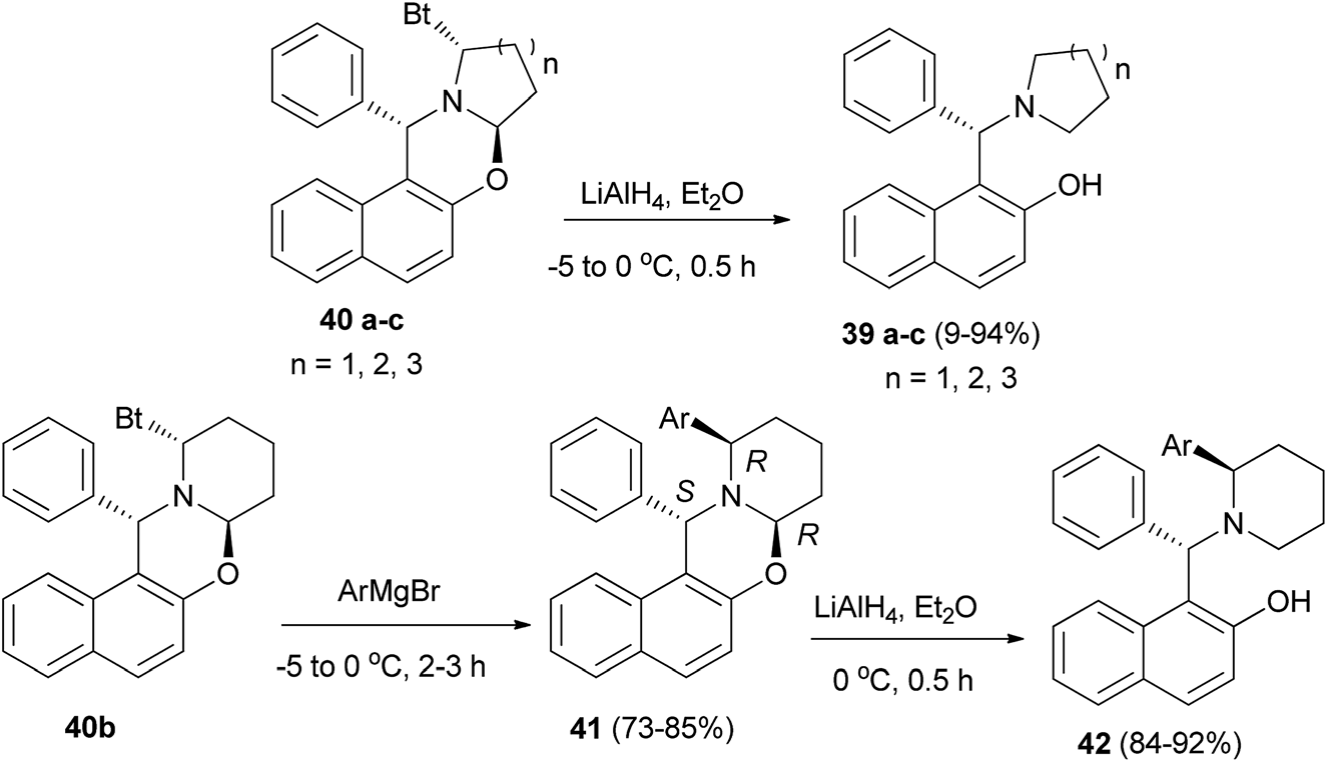
Synthesis of aminonaphthols 39 and 42.

The aminonaphthols 39a–c were obtained in high yields (89–94%) from the reaction of oxazines 40a–c with LiAlH_4_ within half hour at −5 to 0 °C. Also, by using compound 40b as a model, the arylation at its α-position was carried out by treating it with ArMgBr at −5 to 0 °C to give a series of diastereopure compounds 41 after 2–3 h in 73–85% yields. Reductions of 41*via* LiAlH_4_ at 0 °C within 0.5 h yielded a group of highly hindered 1,3-aminophenols (42) in 84–92% yields, which could serve as potential chiral ligands in catalytic asymmetric reactions (Scheme 20).^29^

**Scheme 21:**
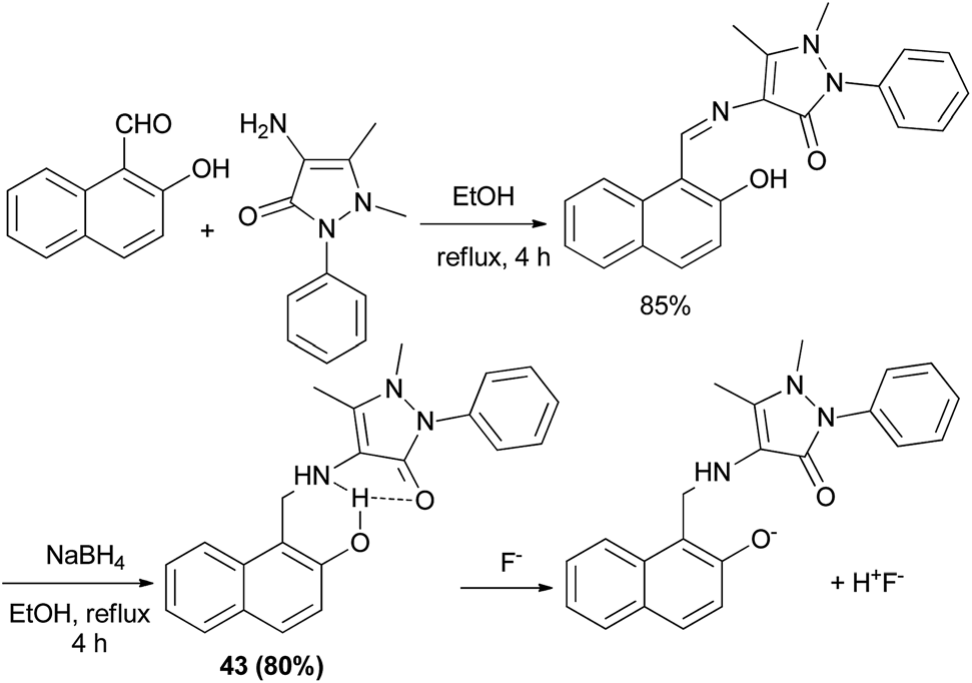
Synthesis of fluorescent chemosensor containing aminonaphthol 43.

Wu *et al.*^30^ introduced a fluorescent chemosensor (43) containing aminonaphthol, which selectively recognizes fluoride anions with high sensitivity. A fluoride anion can strongly interact with a hydrogen-donating group such as hydroxyl or amide through hydrogen bonding interaction to form HF. Aminonaphthol 43 was synthesized from the reaction of 2-naphthol and 4-aminoantipyrine in ethanol at reflux for 4 h. Then reduction with NaBH_4_ in EtOH afforded the desired product after 4 h in 80% yield (Scheme 21).

**Scheme 22:**
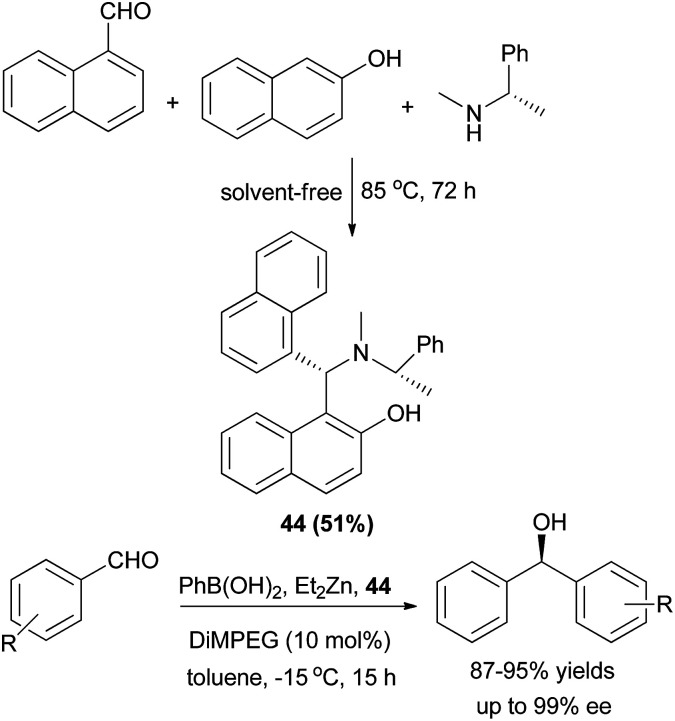
Synthesis of chiral tertiary aminonaphthol 44 and catalyzed enantioselective phenyl transfer to aryl aldehydes.

Chiral tertiary aminonaphthol ligand 44 was synthesized *via* the condensation of 1-naphthaldehyde, (*S*)-(−)-*N*,*R*-dimethylbenzylamine and 2-naphthol in the absence of solvent at 85 °C for 72 h in 51% yield. It served as a highly efficient ligand for the asymmetric catalytic phenyl transfer to aromatic aldehydes and a variety of chiral diarylmethanols was prepared in toluene at −15 °C for 15 h with high ee values (ee up to 99%) and in 87–95% yields (Scheme 22).^31^

**Scheme 23:**
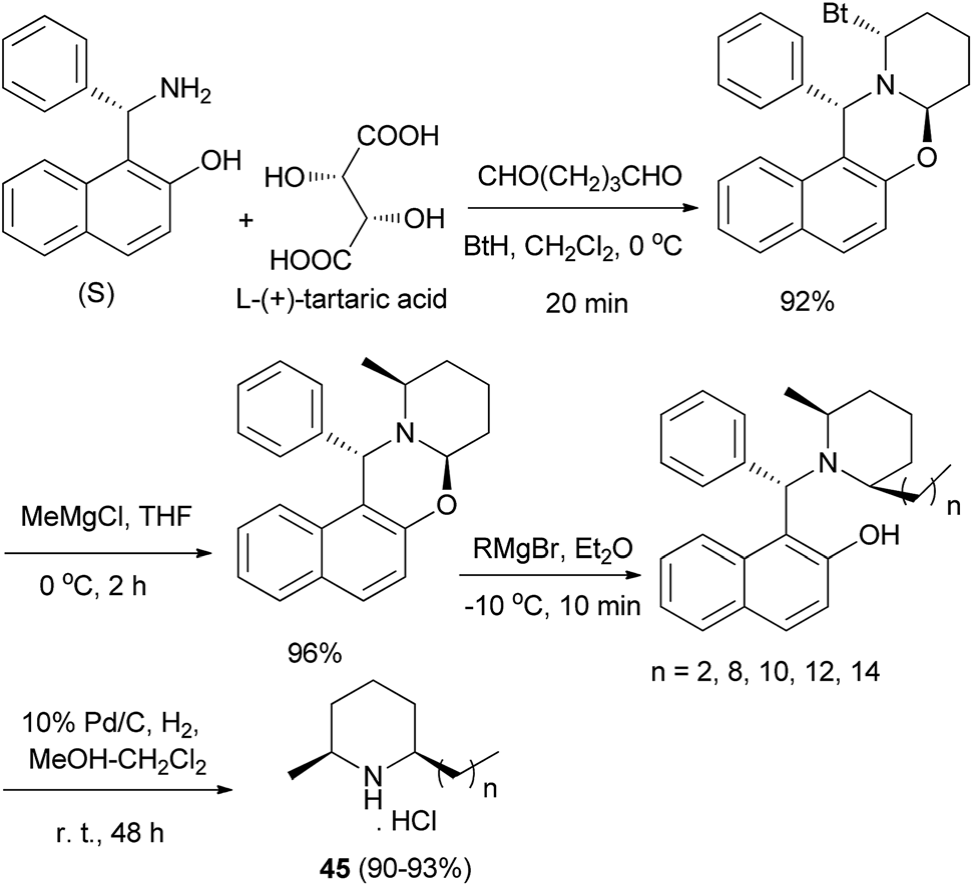
Total syntheses of enantiopure (2*S*,6*R*)-dihydropinidine and (2*S*,6*R*)-isosolenopsins 45.

Total syntheses of enantiopure alkaloidal natural products (2*S*,6*R*)-dihydropinidine (as hydrochloride) and (2*S*,6*R*)-isosolenopsins (as hydrochlorides) (45) were achieved in four steps and in 80–82% total yields by using a synthetic strategy involving the formation and cleavage of 1,3-oxazinane. (*S*)-Betti base proved to be an excellent chiral auxiliary and a novel Pd/C-catalyzed *N*-debenzylation straightforwardly to amine hydrochloride in the presence of CH_2_Cl_2_ was developed (Scheme 23).^32^

**Scheme 24:**
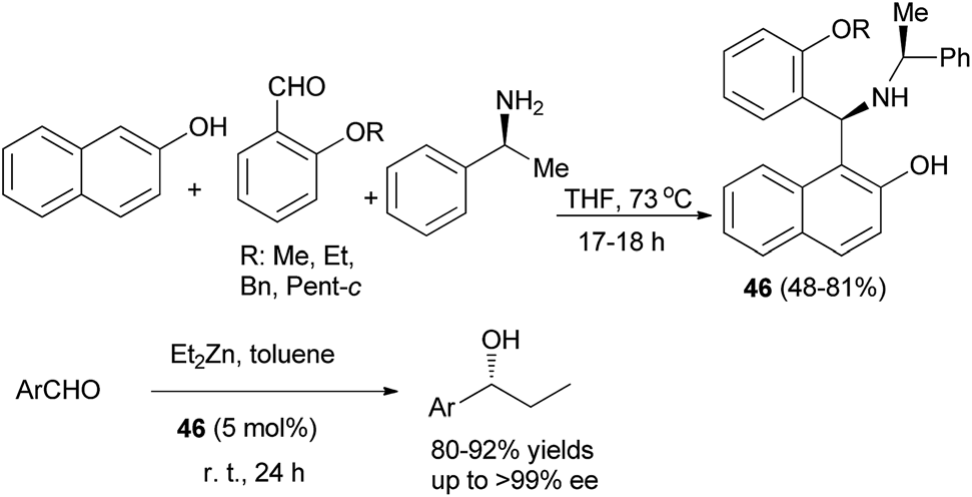
Chiral aminonaphthol 46-catalyzed enantioselective carbonyl addition of diethylzinc to aromatic aldehydes.

Optically active aminonaphthol derivatives 46 were obtained by condensation of 2-naphthol, substituted benzaldehyde, and (*S*)-methylbenzylamine in THF at 73 °C for 17–18 h under an argon atmosphere in 48–81% yields. The addition of diethylzinc to aromatic aldehydes was considerably accelerated by the presence of a catalytic amount of crystalline 46 in toluene at room temperature for 24 h to give, after hydrolysis, the corresponding 1-phenylpropanol in good enantiomeric purity (up to >99% ee) and 80–92% yields (Scheme 24).^33^

**Scheme 25:**
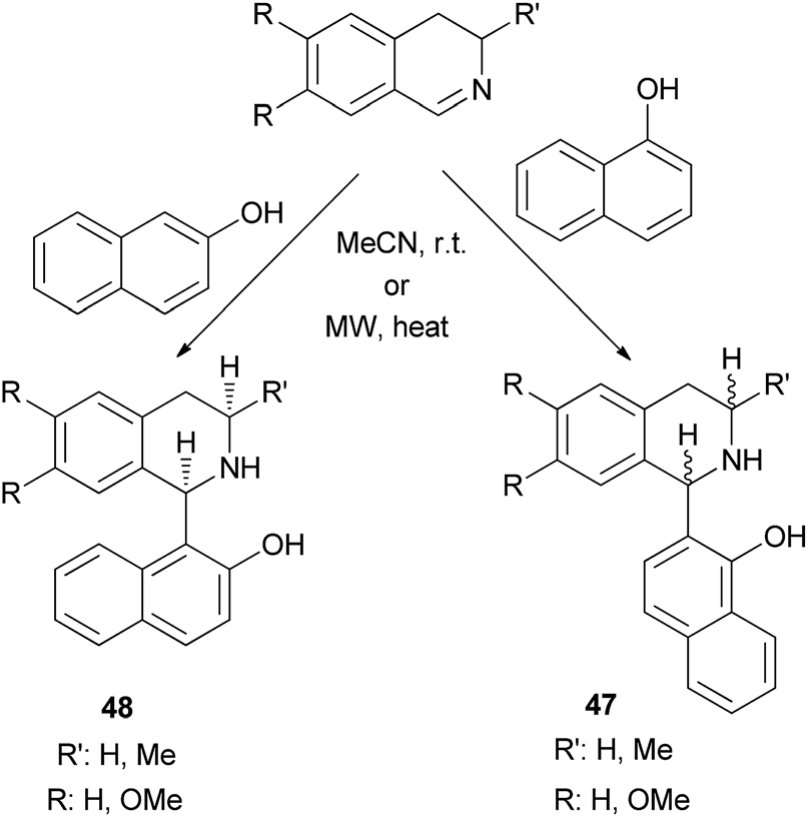
Synthesis of 1-(α- or β-hydroxynaphthyl)-1,2,3,4-tetrahydroisoquinolines 47 and 48.

Solvent-free synthesis of 1-(α- or β-hydroxynaphthyl)-1,2,3,4-tetrahydroisoquinolines 47 and 48 has been described by Fulop *et al.*^34^*via* nucleophilic addition of 1- or 2-naphthol to 3,4-dihydroisoquinolines in MeCN at room temperature for 10–14 days under solvent-free conditions or using microwave irradiation at 70–90 °C within 40–60 min. The reactions yielded the corresponding products in 35–85% yields (Scheme 25).

**Scheme 26:**
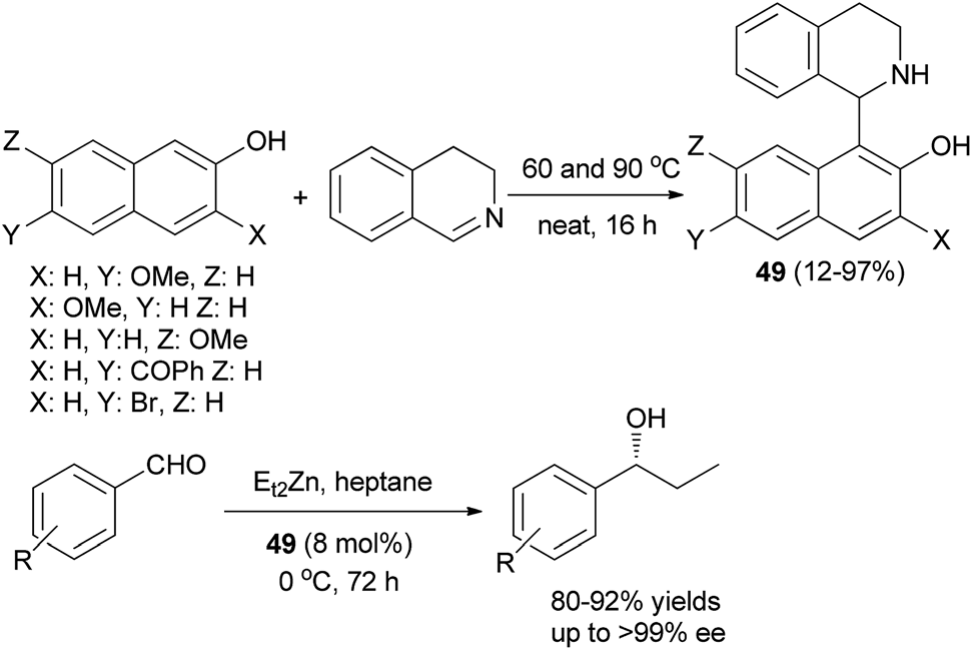
Synthesis of THIQNOL (49) and catalyzed asymmetric addition of diethylzinc to aldehydes.

A self-catalytic aza-Friedel–Crafts method was employed to generate various chiral 1-naphtholyl tetrahydroisoquinoline products (THIQNOL) 49 from 3,4-dihydroisoquinoline and naphthols at 60 and 90 °C under neat conditions after 16 h (Scheme 26). A marked electronic effect was observed in the reaction: 2-naphthols with electron-donating substituents gave excellent yields (62–97%), whereas 2-naphthols with electron-withdrawing substituents gave lower yields (12–54%). A method was developed to resolve the two enantiomers of a THIQNOL derivative. The enantiomerically pure derivative showed moderate catalytic activity in the asymmetric diethylzinc addition to aldehydes and afforded, enantioselectively, 1-propanol derivatives (up to 70% ee) in heptane-toluene at 0 °C within 72 h in 55–85% yields.^35^

**Scheme 27:**
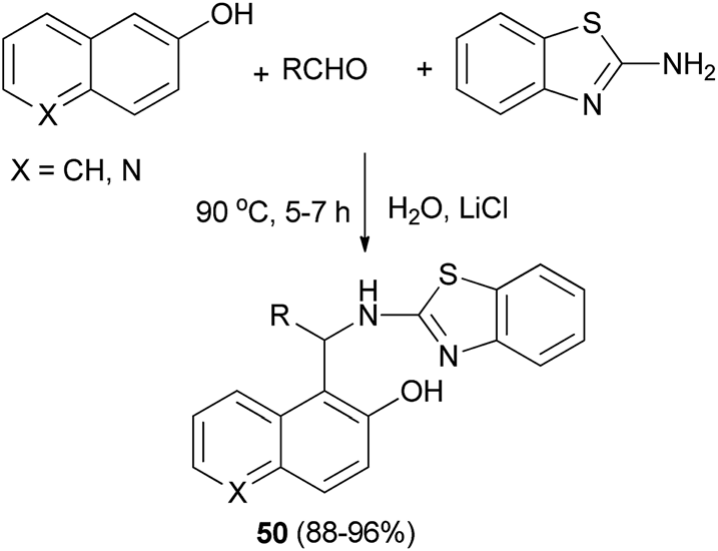
Water-promoted one-pot synthesis of compound 50.

2′-Aminobenzothiazolomethylnaphthols or 5-(2′-aminobenzothiazolomethyl)-6-hydroxyquinolines 50 could also be achieved in 88–96% yields by warming aldehydes and 2-aminobenzothiazole with 2-naphthol or 6-hydroxyquinoline in water containing LiCl as ionic solute for the increasing hydrophobic effect of water at 90 °C after 5–7 h (Scheme 27). When the reaction was carried out in the absence of LiCl, a decreased yield (12–16%) was obtained.^36^

**Scheme 28:**
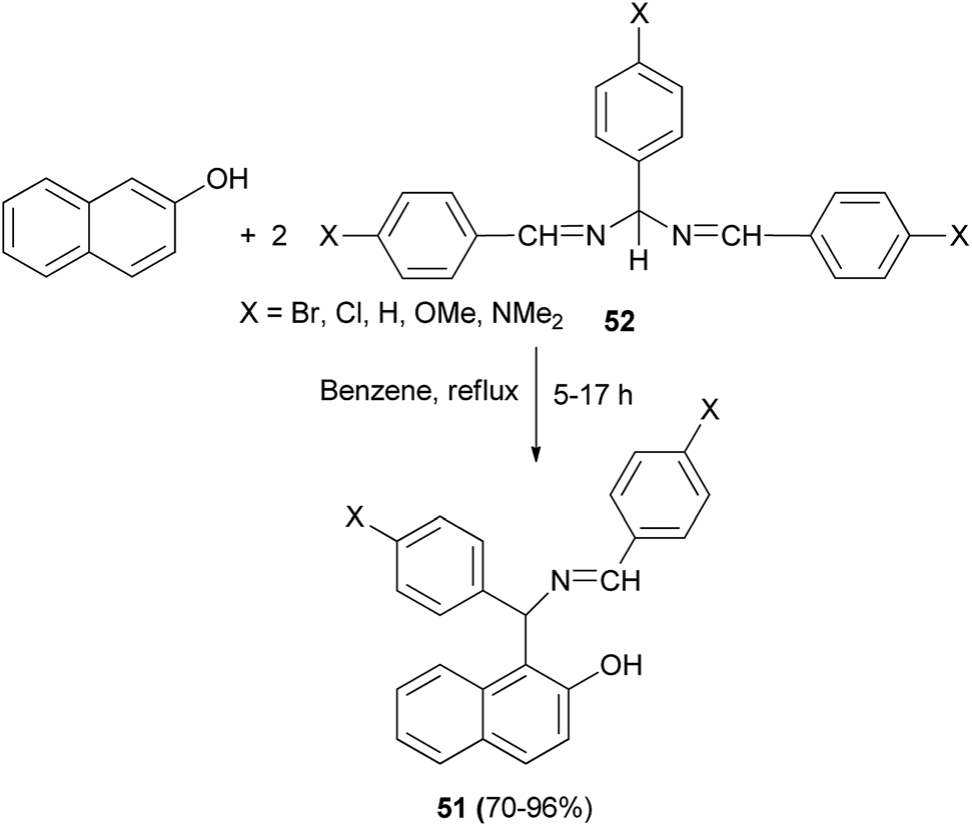
Synthesis of benzylidene derivatives 51.

In 2007, Alfonsov *et al.*^37^ reported a new approach to the synthesis of benzylidene derivatives of 1-(α-aminobenzyl)-2-naphthols (Betti bases) 51, by reaction of 2-naphthol with 1,3,5-triaryl-2,4-diazapenta-1,4-dienes (3 : 2) 52 in refluxing benzene for 5–17 h. This method provides desired products in high yields (70–96%) and purity, facilitating subsequent separation into the individual enantiomers (Scheme 28).

**Scheme 29:**
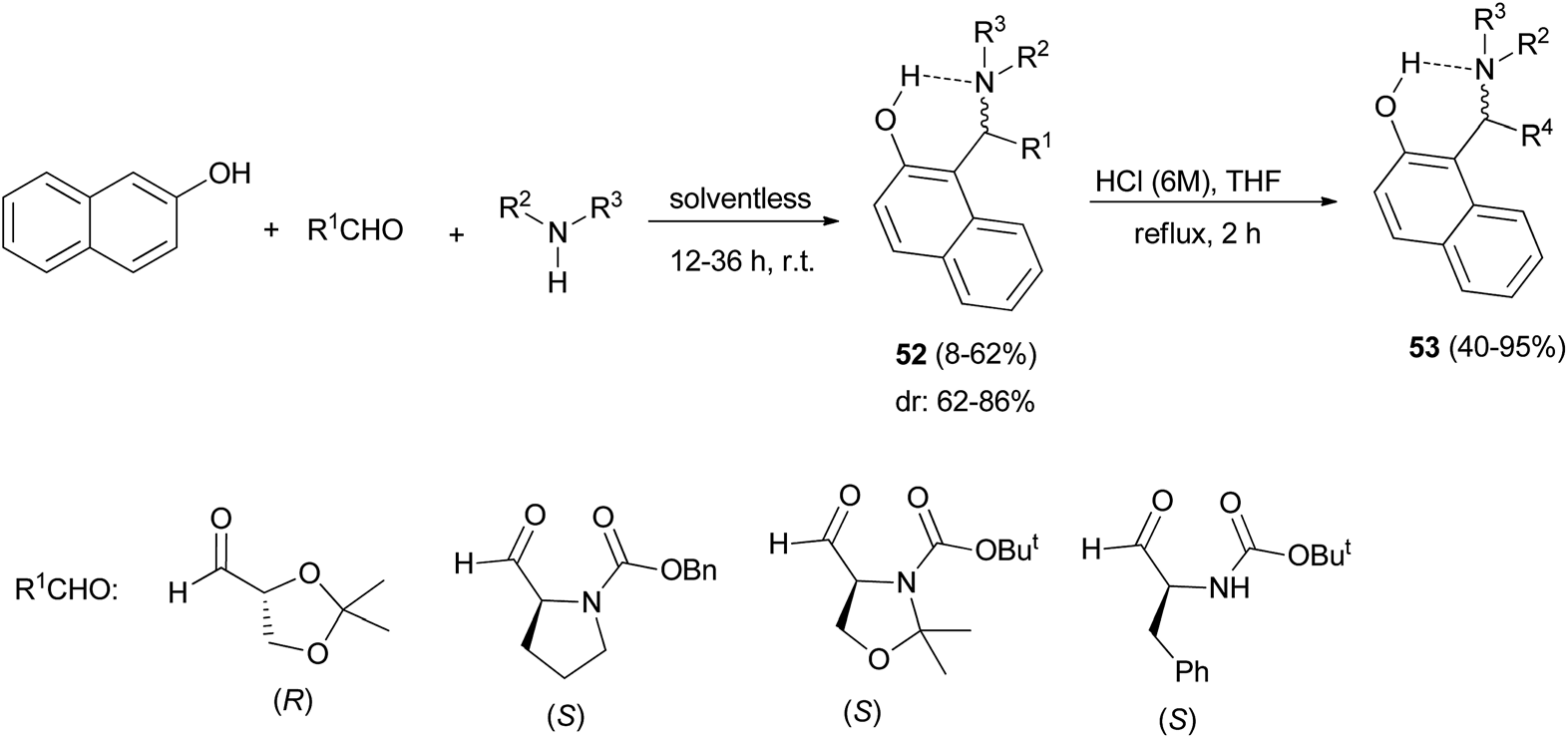
Stereoselective synthesis of aminoalkylnaphthols 52 and 53.

Palmieri *et al.*^38^ introduced a short and stereoselective synthesis of vicinal aminodiols, diamines and diaminols in good yields, through a three-component aromatic Mannich-type reaction. Initially, the reaction of 2-naphthol, enantiopure aldehydes containing stereogenic centres and amines at room temperature in solventless conditions for 12–36 h afforded a mixture of two diastereomers of aminoalkylnaphthols 52 in 8–62% yields. Then, the obtained aminoalkylnaphthols 52 were subjected to acid hydrolysis (HCl aq./THF) under reflux conditions for 2 h, to deprotect amino and hydroxyl groups in aldehydes resulting in enantiopure aminoalkylnaphthols 53 in 40–95% yields (Scheme 29).

**Scheme 30:**
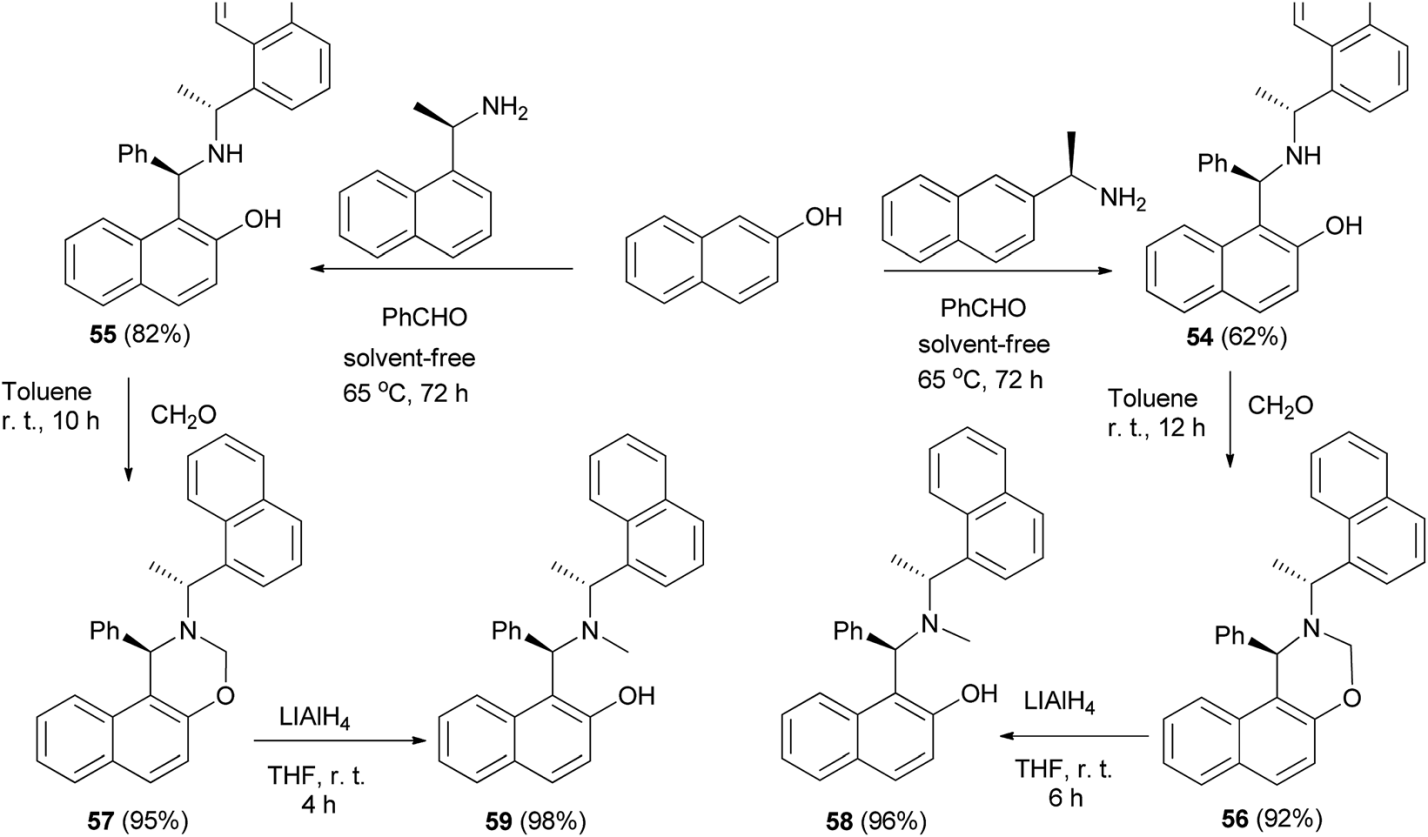
Synthesis of optically active aminonaphthols.

Fulop *et al.*^39^ achieved optically active aminonaphthols 54 and 55 by condensation of 2-naphthol, benzaldehyde and (*R*)-(+)-1-(1-naphthyl)ethylamine or (*R*)-(+)-1-(2-naphthyl)ethylamine at 65 °C within 72 h under solvent-free conditions. Then, the reaction of aminonaphthols 54 and 55 with paraformaldehyde in toluene at room temperature for 10 and 12 h afforded naphthoxazines 56 and 57, respectively. Naphthoxazines were reduced to chiral aminonaphthols 58 and 59 in THF at room temperature in 4 and 6 h by LiAlH_4_ (Scheme 30).

**Scheme 31:**
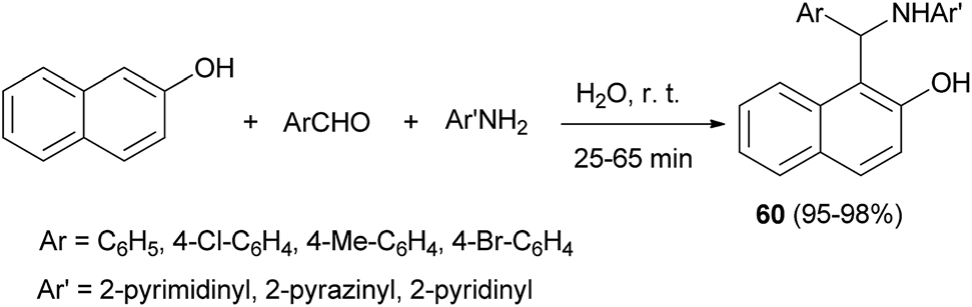
Synthesis of aminonaphthols 60 in water.

Ghandi *et al.*^40^ described the reaction of 2-naphthol, aromatic aldehyde, and heteroarylamine in water at room temperature for 25–65 min, furnishing the 1-(aryl(heteroarylamino)methyl)naphthalene-2-ols 60 in 95–98% yields for the first time (Scheme 31).

**Scheme 32:**
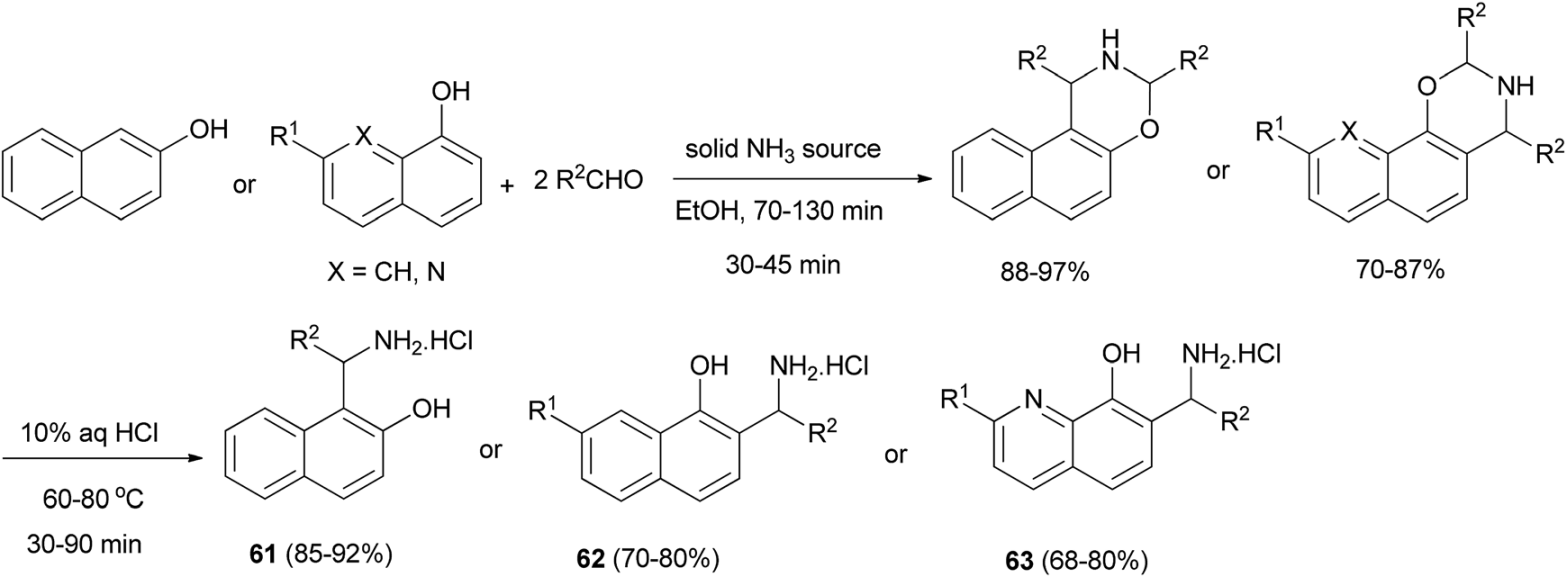
Microwave-assisted synthesis of (aminoalkyl)naphthols 61, 62 and (aminoalkyl)quinolinols 63.

Also, Fulop *et al.*^41^ reported that ammonium carbamate and ammonium hydrogen carbonate can be used as very effective solid ammonia sources to prepare different (aminoalkyl)naphthols 61 and 62 and (aminoalkyl)quinolinols 63 in ethanol and water as solvents under microwave conditions in modified three-component Mannich reactions. Depending on the solid ammonia source, the yields of the desired products obtained were 68–92% (Scheme 32).

**Scheme 33:**
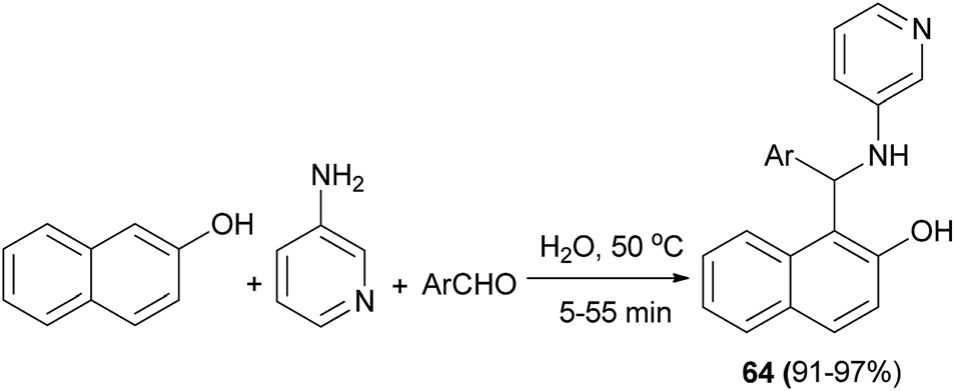
Synthesis of *N*-heteroarylaminonaphthols 64.

The reaction of 2-naphthol, 3-aminopyridine, and aromatic aldehydes in water at 50 °C afforded a number of *N*-heteroarylaminonaphthols 64 after 5–55 min in 91–97% yields (Scheme 33).^42^

**Scheme 34:**
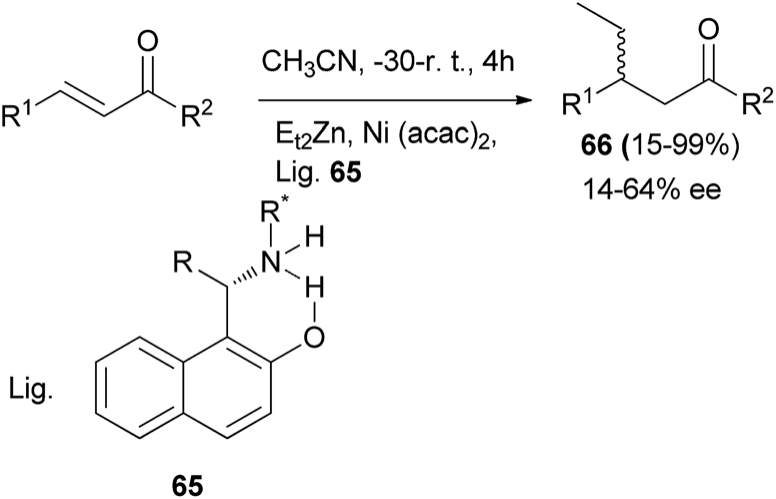
Enantioselective addition of diethylzinc to chalcones using ligand 65.

Palmieri *et al.*^43^ demonstrated that 1-(aminoalkyl)naphthols 65 can be used as ligands in the nickel-catalyzed enantioselective addition of organozinc to chalcones in CH_3_CN at −30 °C to room temperature for 4 h and afforded the corresponding products 66 in 15–99% yields and 14–64% ee (Scheme 34).

**Scheme 35:**
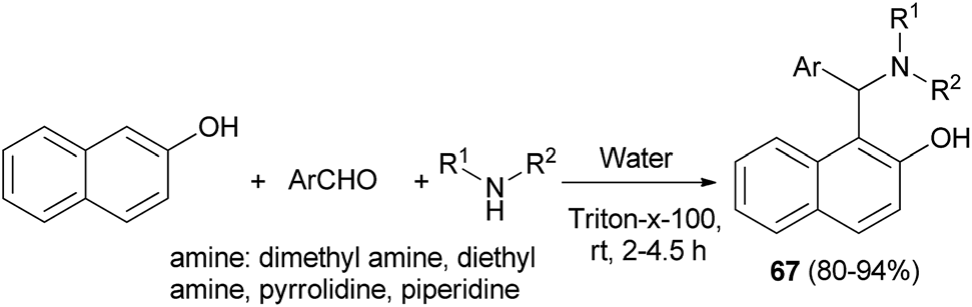
Triton X-100-catalyzed synthesis of Betti bases 67.

Kumar *et al.*^44^ succeeded in the preparation of Betti bases 67 from secondary amine, aromatic aldehydes and 2-naphthol using a non-ionic surfactant (Triton X-100, 5 mol%) at room temperature. All the aromatic aldehydes reacted almost equally well to afford Betti bases after 2–4.5 h in excellent yields (80–94%) (Scheme 35).

**Scheme 36:**
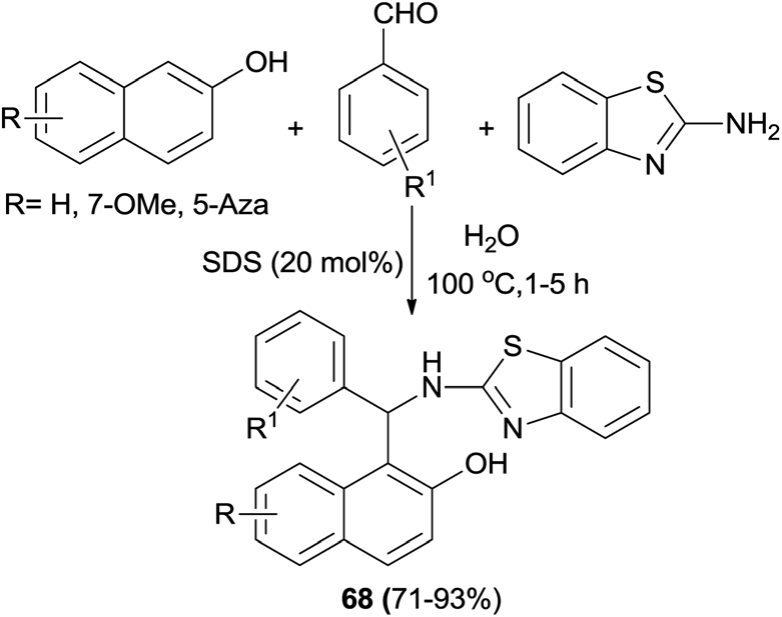
Sodium dodecylsulfate-assisted synthesis of 1-(benzothiazolylamino)methyl-2-naphthols 68 in water.

Kumar *et al.*^45^ have devoted considerable attention to a sodium dodecylsulfate (20 mol%)-catalyzed aminoalkylation of 2-naphthols with aldehydes and 2-aminobenzothiazole in water at reflux. The corresponding products 68 were isolated after 1–5 h in 71–93% yields (Scheme 36).

**Scheme 37:**
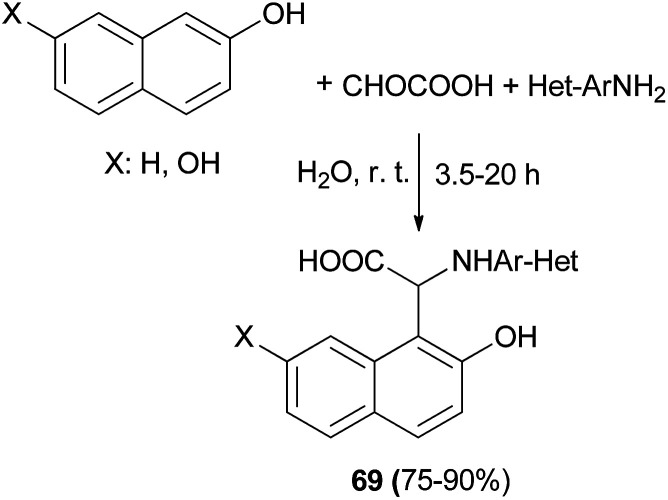
Synthesis of α-naphthylglycine derivatives 69 in water.

**Scheme 38:**
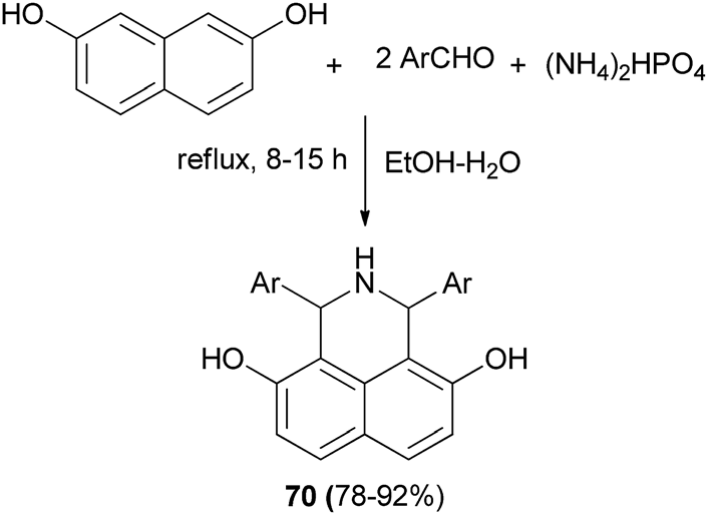
Symmetric aminoalkylation of 2,7-naphthalenediol.

A series of novel *N*-heteroaryl α-arylglycines 69 containing naphthol rings has been prepared by one-pot, three-component condensation reactions of glyoxalic acid, heteroarylamines and naphthols. The reactions were performed in water at ambient temperature within 3.5–20 h which afforded desired products in 75–90% yields (Scheme 37).^46^ A one-pot procedure was reported by Foroughifar *et al.* for the preparation of 4,9-dihydroxy-1,3-diaryl-2,3-dihydro-2-zaphenalenes 70 in 78–92% yields after 8–15 h from aromatic aldehydes, 2,7-naphthalenediol and ammonium hydrogen phosphate ((NH_4_)_2_HPO_4_) in a mixture of ethanol-water (3 : 1) under reflux conditions (Scheme 38).^47^

**Scheme 39:**
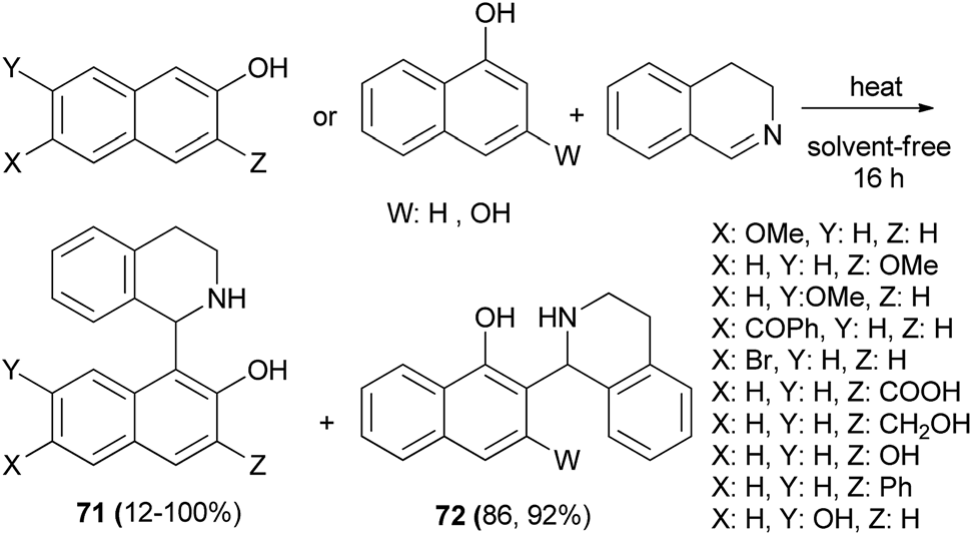
Synthesis of 1-naphthoyltetrahydroisoquinolines 71 and 72.

A self-catalytic protocol was developed using an aza-Friedel–Crafts method to generate 1-naphthoyltetrahydroisoquinoline products 71 and 72 in 12–100% yields under solvent-free conditions. The reaction proceeded at 60, 80 and 90 °C within 16 h in the absence of any additional catalyst (Scheme 39).^48^

**Scheme 40:**
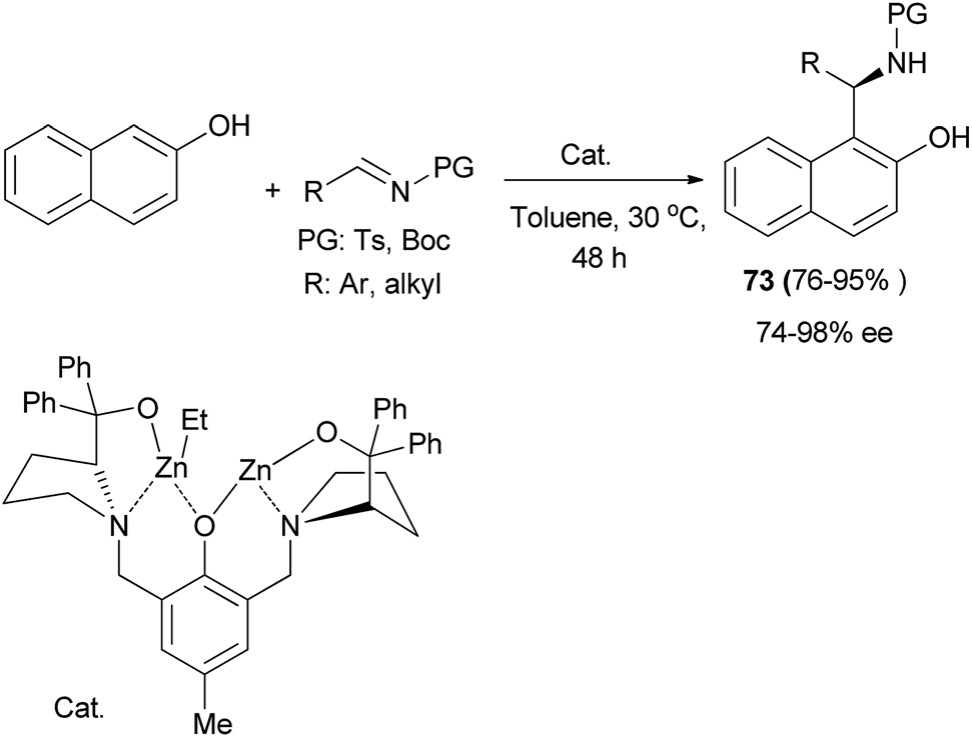
Asymmetric synthesis of Betti base derivatives 73.

Hui *et al.*^49^ have reported an asymmetric aza-Friedel–Crafts reaction of 2-naphthol with tosylimines catalyzed by a dinuclear zinc complex. The expected products 73 were obtained in 76–95% yields and good to excellent enantioselectivities of 74–98% ee in toluene at 30 °C after 48 h (Scheme 40).

**Scheme 41:**
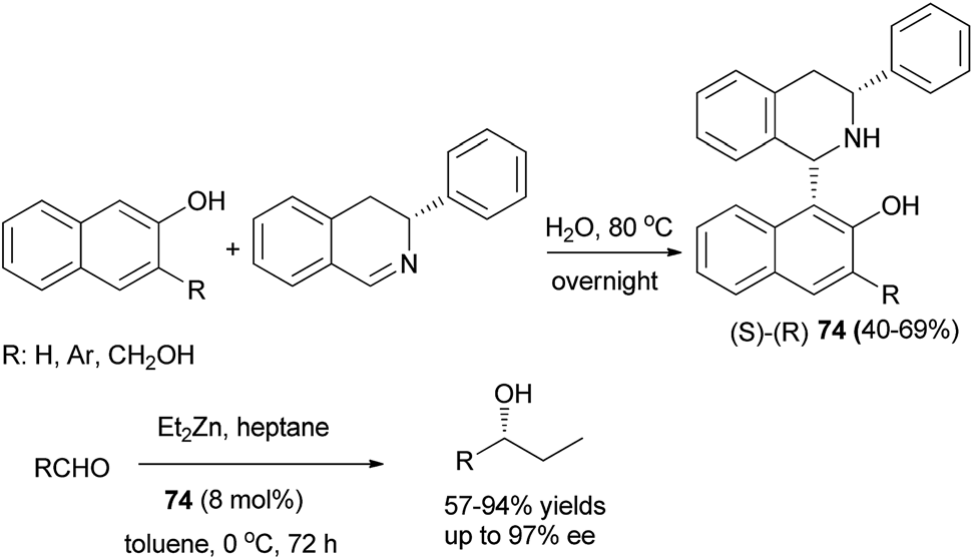
Synthesis of Betti bases 74 and their use in the asymmetric addition of diethylzinc to aldehydes.

Addition of (*R*)-3-phenyl-3,4-dihydroisoquinoline to 2-naphthols in water at 80 °C overnight led to the formation of (*S*)-(*R*)-1,3-disubstituted tetrahydroisoquinolines 74 as chiral ligands in 40–69% yields (Scheme 41).^50^ These chiral ligands were then used to catalyze asymmetric addition of diethylzinc to aldehydes in toluene at 0 °C for 72 h and the desired products were achieved in 57–93% yields with high enantioselectivities (up to 97% ee).

**Scheme 42:**
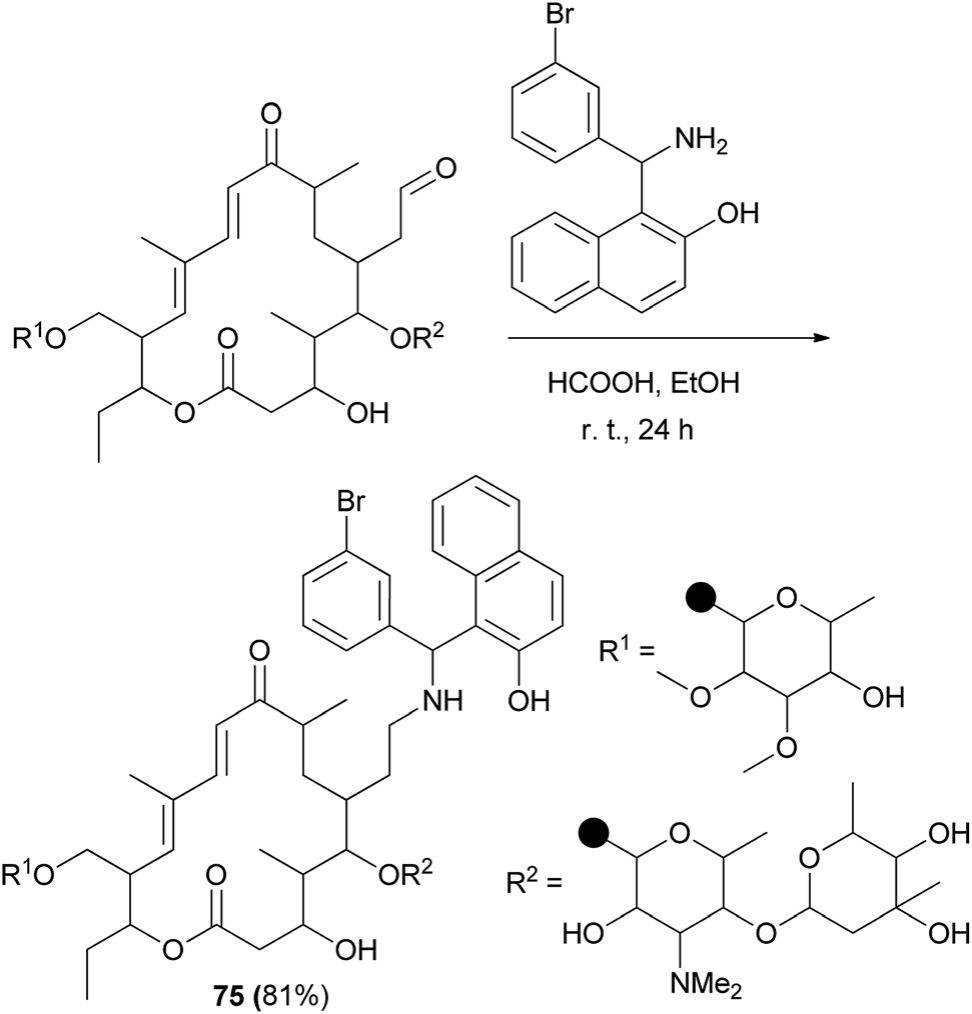
Synthesis of Betti base derivatives of tylosin (75).

TBN (*N*-tylosil-1-α-amino(3-bromophenyl)methyl-2-naphthol; 75) was prepared from the reaction of tylosin tartrate, the Betti base 1-α-amino(3-bromophenyl)methyl-2-naphthol and formic acid in EtOH at room temperature for 24 h in 81% yield (Scheme 42). Investigation of results indicated that TBN is a potent modulator of the P-gp membrane pump and that the compound could be of clinical relevance to improve the efficacy of chemotherapy in MDR cancers.^51^

**Scheme 43:**
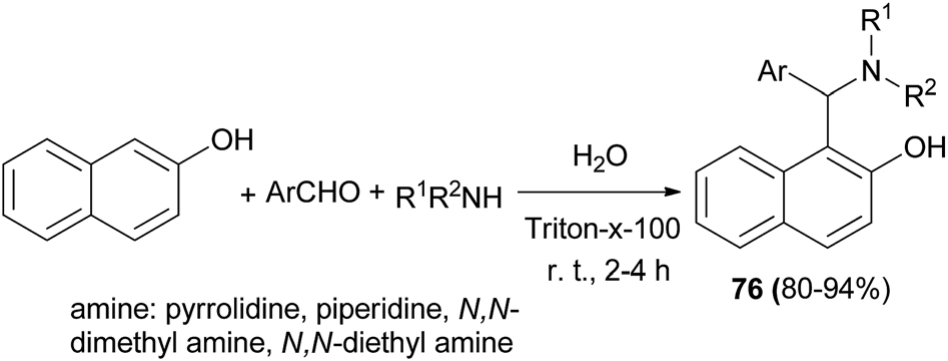
Non-ionic surfactant-catalyzed synthesis of Betti bases 76 in water.

Triton X-100 as a non-ionic surfactant catalyst was used for the synthesis of Betti bases 76 from secondary amine, aromatic aldehydes, and 2-naphthol using Mannich-type reaction in water at room temperature. The catalyst gave the best results and the reaction proceeds through imine formation, which is stabilized by colloidal dispersion and undergoes nucleophilic addition to afford the corresponding *N*,*N*-dialkylated Betti bases in excellent yields (80–94%) after 2–4 h (Scheme 43).^52^

**Scheme 44:**
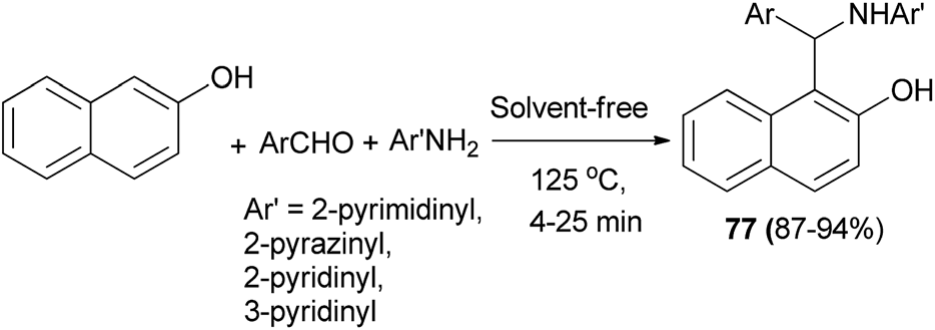
Synthesis of *N*-heteroarylaminonaphthols 77.

Olyaei *et al.*^53^ demonstrated a convenient and efficient method for the synthesis of *N*-heteroarylaminonaphthols 77 by using heteroarylamines such as 2-aminopyrimidine, 2-aminopyrazine, 2-aminopyridine and 3-aminopyridine under solvent-free conditions at 125 °C. The reactions completed in 4–25 min with products obtained in 87–94% yields (Scheme 44).

**Scheme 45:**
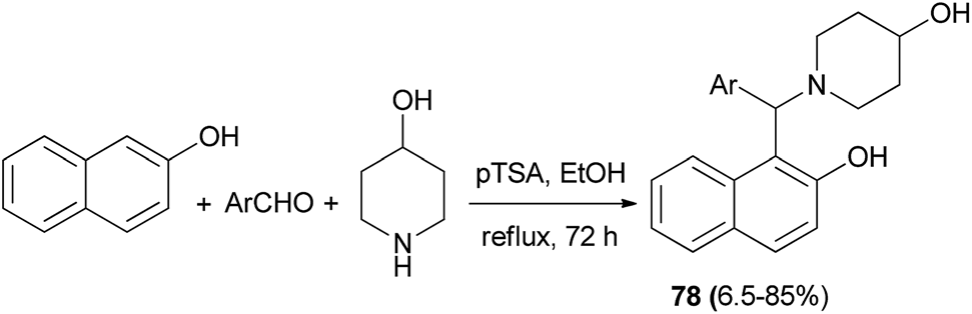
Synthesis of racemic 2-naphthol Mannich bases 78.

Jha *et al.*^54^ have devoted considerable attention to an efficient synthesis of 1-((2-hydroxynaphthalen-1-yl)arylmethyl)piperidin-4-ol prototypes 78 as racemic mixtures *via* the Mannich reaction protocol from 2-naphthol, 4-piperidinol, and different aromatic aldehydes. The reaction proceeded in the presence of *p*-TSA as catalyst in ethanol at reflux and was complete within 72 h to produce the corresponding products in 6.5–85% yields (Scheme 45). These chiral Mannich bases were then resolved utilizing an enzyme-assisted chemo-, regio-, and enantioselective (Novozyme 435®) acetylation process in CHCl_3_.

**Scheme 46:**
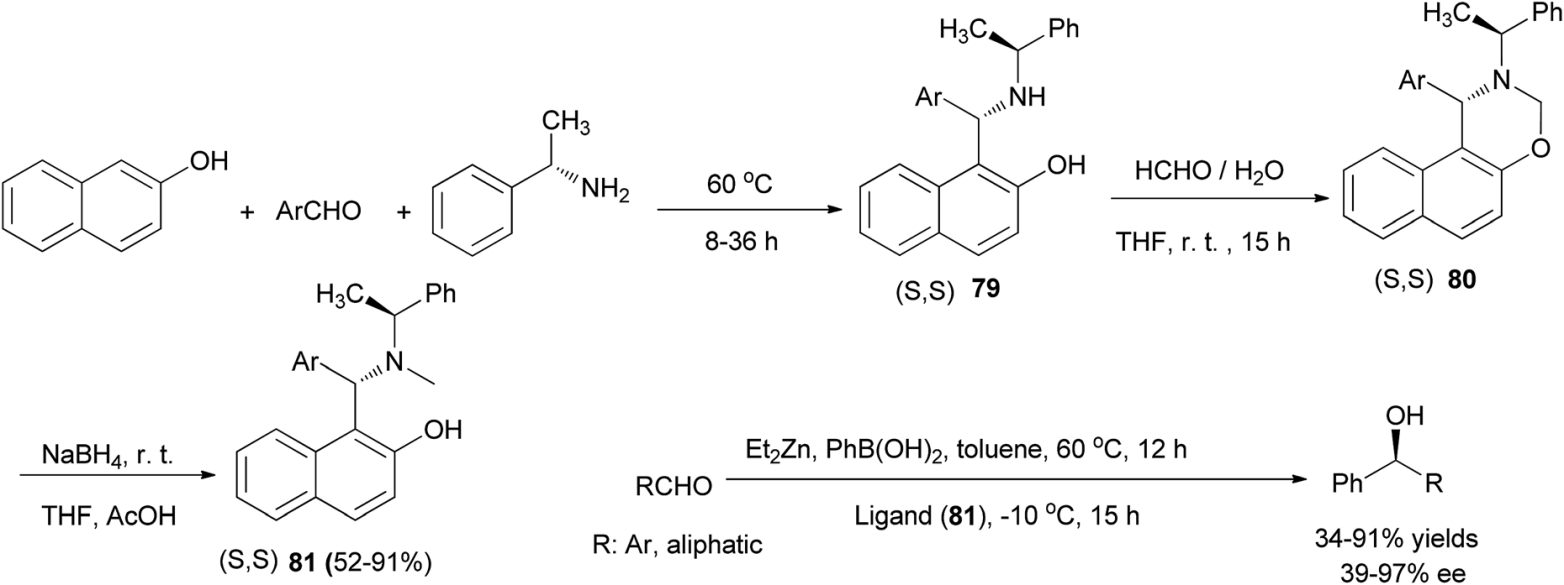
The preparation of chiral aminonaphthol ligands 79 and 81.

Chan *et al.*^55^ reported the synthesis of chiral aminonaphthol ligands 79 from the reaction of 2-naphthol, (*S*)-1-phenylethylamine, and aldehydes at 60 °C under solvent-free conditions within 8–36 h. Then, the reaction of aminonaphthols 79 with 35% aqueous formaldehyde in THF at room temperature afforded naphthoxazines 80, and addition of NaBH_4_ to the solution of naphthoxazines in THF and AcOH at room temperature led to the formation of chiral tertiary aminonaphthol ligands 81 in 52–91% yields. The results of asymmetric phenyl transfer to aromatic aldehydes catalyzed by these chiral ligands in toluene indicated that enantioselectivities (up to 97% ee) were greatly influenced by the electronic and steric effects of the ligands 81 (Scheme 46).

**Scheme 47:**
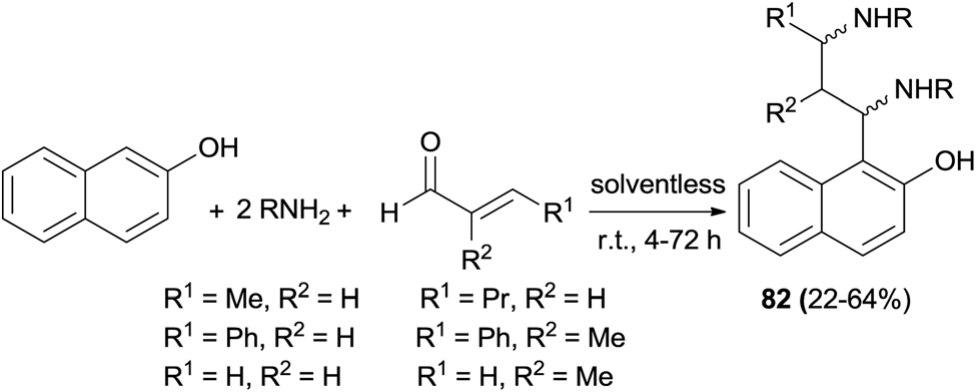
Stereoselective synthesis of vicinal diaminoalkylnaphthols 82.

In 2011, stereoselective synthesis of vicinal diaminoalkylnaphthols 82 in 22–64% yields was reported by Cimarelli *et al.*^56^ The desired products were obtained by three-component Mannich-type reaction of α,β-unsaturated aldehydes with 2-naphthol and amines (benzylamine, pyrrolidine and 1-phenylethanamine) under solventless condition at room temperature within 4–72 h (Scheme 47). The relative and absolute configurations of the products obtained were assigned on the basis of the ^3^*J* values of ^1^H NMR spectra, in combination with the conformational analysis of the molecules done by molecular modelling.

**Scheme 48:**
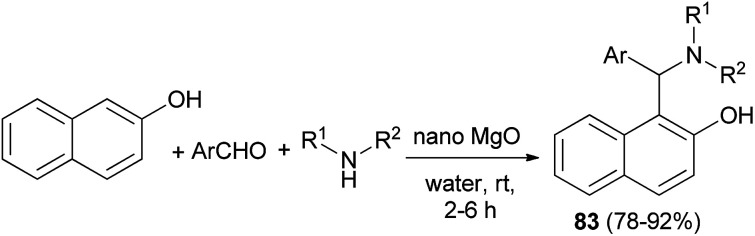
Synthesis of Betti bases 83 over nanocrystalline MgO catalyst.

An eco-friendly method for the synthesis of the Betti bases 1-(α-aminoalkyl)naphthols 83 has been carried out over a basic nanocrystalline MgO catalyst in aqueous condition at room temperature. The reactions worked well with almost all the aldehydes and aliphatic amines within 2–6 h and gave the corresponding products in 78–92% yields (Scheme 48). Surprisingly, the reaction was not successful with aromatic amines which might be due to their reduced nucleophilicity.^57^

**Scheme 49:**
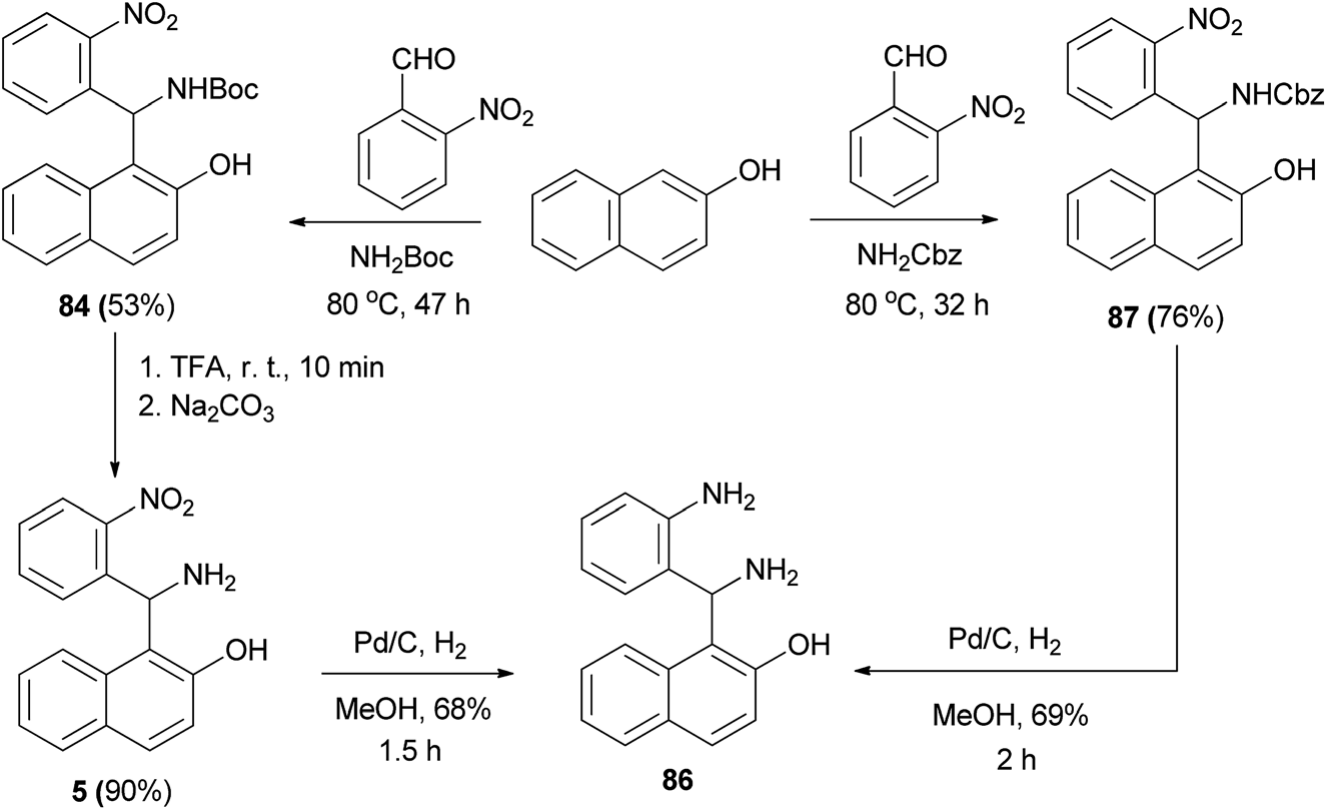
Synthesis of 1-(amino(2-aminophenyl)methyl)-2-naphthol 87.

Nitro derivative 84 was achieved from treatment of 2-naphthol, 2-nitrobenzaldehyde and *tert*-butyl carbamate under solvent-free conditions for 47 h at 80 °C in 53% yield. In the following experiment, the Boc group was removed with trifluoroacetic acid, resulting in 85 in 90% yield. This step was followed by reduction of the nitro group by means of catalytic (Pd/C) hydrogenation, yielding 1-(amino(2-aminophenyl)methyl)-2-naphthol (86) (68%). Also, using the reaction of 2-naphthol, 2-nitrobenzaldehyde and benzyl carbamate under solvent-free conditions, 87 was synthesized at 80 °C after 32 h in 76% yield. Removal of the protecting group and reduction of the nitro group were accomplished in one step by catalytic (Pd/C) hydrogenation, yielding 86 (69%) (Scheme 49).^58^

**Scheme 50:**
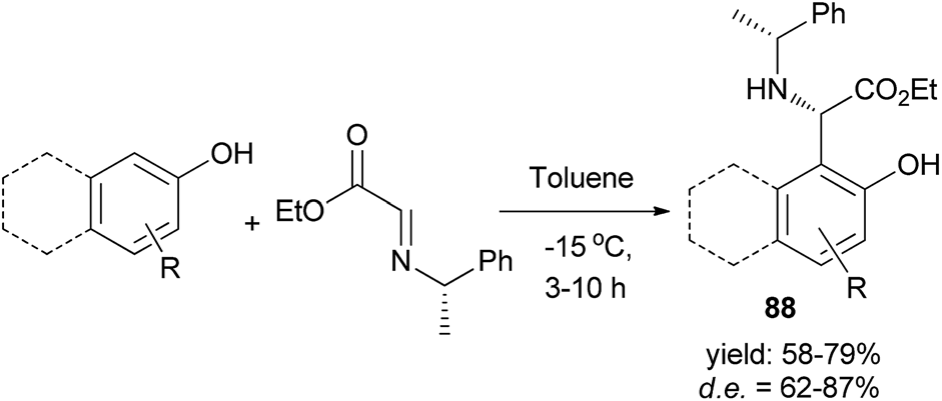
Spontaneous asymmetric synthesis of non-natural hydroxyaryl glycinates 88.

A synthesis of useful enantiomerically pure arylglycinates 88*via* spontaneous reaction between phenol or naphthol derivatives and enantiopure α-imino glyoxylate in toluene at −15 °C within 3–10 h in the absence of an acid catalyst was reported. A library of enantiopure substituted phenol or naphthol glycinates was obtained in 58–79% yields and high diastereoselectivities (62–87%) (Scheme 50). Diastereomerically pure aryl glycinates were obtained *via* flash chromatographic separation of the crude reaction mixture.^59^

**Scheme 51:**
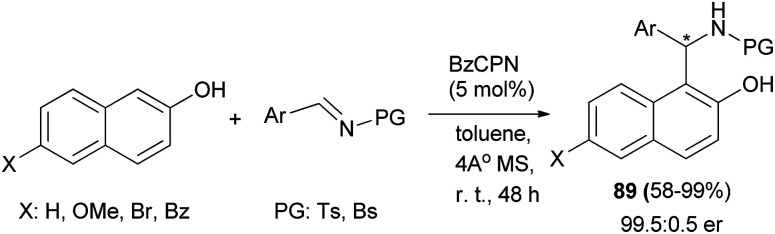
BzCPN-catalyzed asymmetric synthesis of aza-Friedel–Crafts products 89.

**Scheme 52:**
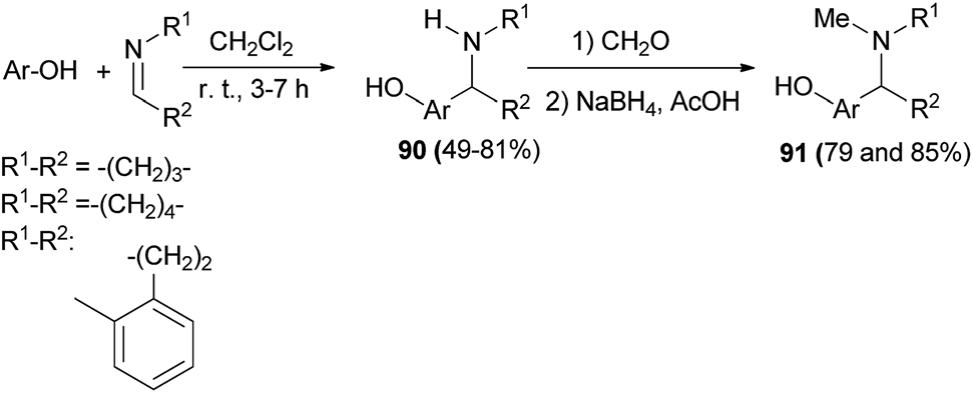
Synthesis of aminocycloalkylnaphthols 90 and 91.

The cupreine-derived bifunctional organocatalyst BzCPN efficiently catalyzes the formation of aza-Friedel–Crafts products 89 in toluene and 4 Å MS as the additives from naphthols and *N*-sulfonylimines within 48 h in good to excellent yields (up to 99%) with high enantioselectivities (up to 99.5 : 0.5 er) under mild reaction conditions, with a low catalyst loading (5 mol%), and in an aerobic environment (Scheme 51).^60^ A large library of aminocycloalkylnaphthols 90 is obtained by the Betti reaction between activated naphthols (1-naphthol, 2-naphthol and 4-methoxy-1-naphthol) and five- and six-membered cyclic imines in CH_2_Cl_2_ within 3–7 h at room temperature in 49–81% yields. Betti base derivatives 90 were methylated at the nitrogen atom by cyclization with formaldehyde to the corresponding oxazolidines, followed by reduction with sodium triacetoxyborohydride in THF, to form the corresponding products 91 in yields of 79 and 85% (Scheme 52).^61^

**Scheme 53:**
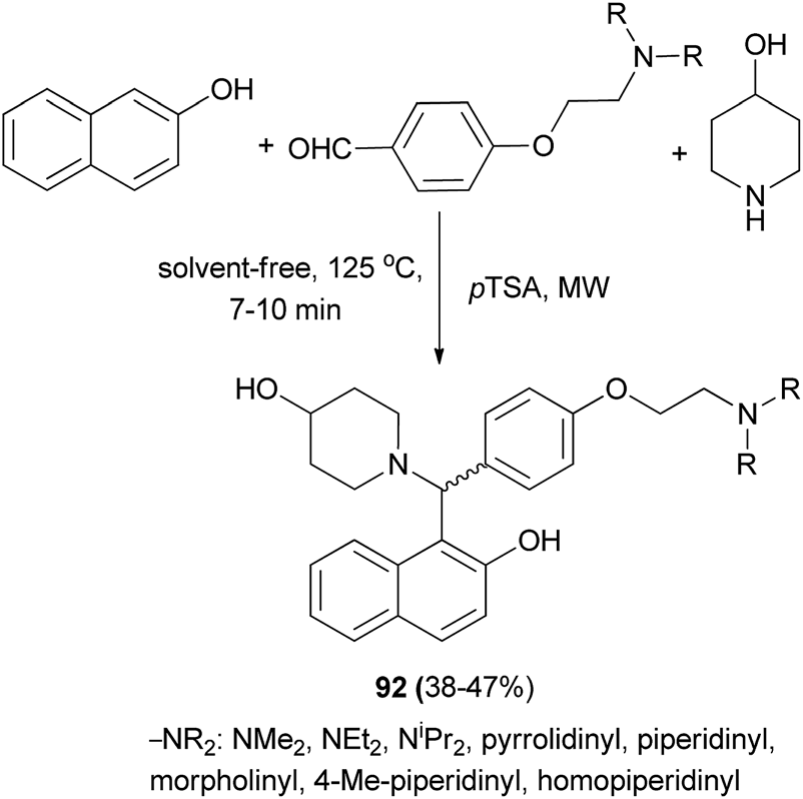
Synthesis of racemic Betti bases 92.

**Scheme 54:**
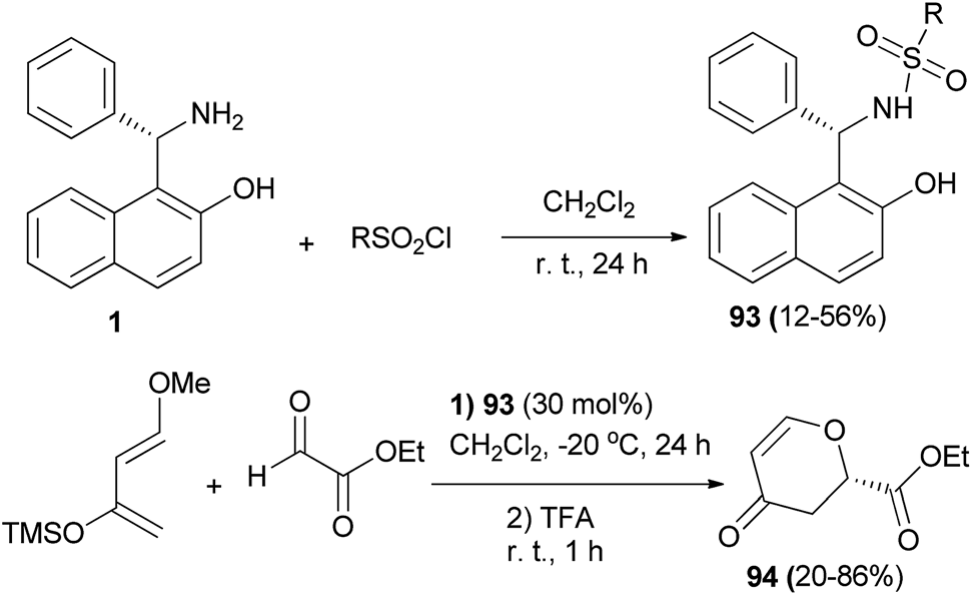
Sulfonamide 93-catalyzed asymmetric hetero-Diels–Alder reaction of ethyl glyoxylate with Danishefsky's diene.

Aminoalkylnaphthol derivatives 92 were obtained in 38–47% yields by performing a Mannich reaction between 2-naphthol, 4-piperidinol and appropriate 4-(2-(dialkylamino)ethoxy)benzaldehydes in the presence of catalytic amounts of *p*-toluenesulfonic acid in a microwave reactor after 7–10 min (Scheme 53).^62^

(*S*)-Betti base 1 was converted into sulfonamide organocatalysts 93 by the reaction with corresponding sulfonyl chlorides in the presence of pyridine in CH_2_Cl_2_ at room temperature for 24 h in 12–56% yields. Next, the hetero-Diels–Alder reaction of ethyl glyoxylate with Danishefsky's diene was carried out in a catalytic manner using the chiral sulfonamide 93 (30 mol%) in CH_2_Cl_2_ at −20 °C for 24 h, followed by treatment with TFA at room temperature for 1 h to obtain corresponding 2-substituted 2,3-dihydropyran-4-ones 94 in 20–86% yields (Scheme 54).^63^

**Scheme 55:**
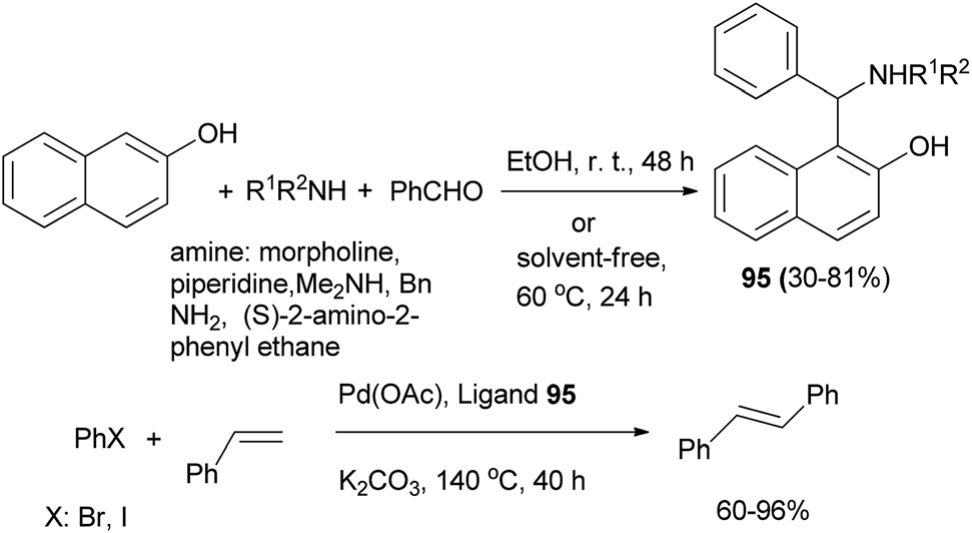
Pd-catalyzed Mizoroki–Heck reaction with Betti bases 95.

**Scheme 56:**
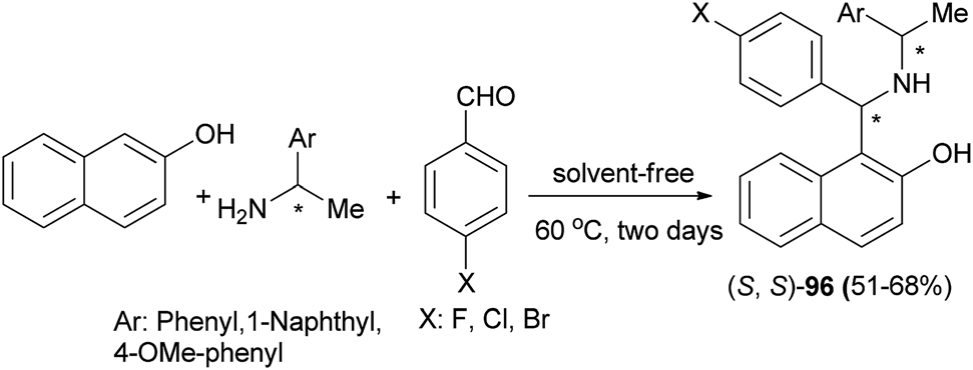
Stereoselective Betti reaction between 2-naphthol, arylaldehydes and (*R*)- or (*S*)-1-arylethylamine.

Bedekar and Chaudhary^64^ achieved 1-(α-aminobenzyl)-2-naphthols 95 by the reaction of 2-naphthol, benzaldehyde and primary or secondary amines in absolute alcohol at room temperature for 48 h or under solvent-free condition at 60 °C for 24 h in 30–81% yield. Compounds 95 were used as ligands in palladium-catalyzed Mizoroki–Heck reaction in dimethylacetamide in the presence of K_2_CO_3_ at 140 °C for 40 h with a variety of substrates and afforded *trans*-stilbene in 60–96% yields. High turnover numbers are observed for the reactions with both aryl bromides and iodides, while aryl chlorides are inert (Scheme 55).

(*S*,*S*)-Aminobenzylnaphthols 96 were obtained by a Betti reaction of 2-naphthol, benzaldehyde or *p*-halobenzaldehyde with (*R*)- or (*S*)-1-arylethylamine for two days at 60 °C without any solvent in 51–68% yields (Scheme 56). The crystal structures of (*S*,*S*)-aminobenzylnaphthols, easily produced by a chromatography-free highly stereoselective Betti reaction, were investigated by means of single-crystal X-ray diffraction analysis, and the main intra- and intermolecular interactions were described.^65^

**Scheme 57:**
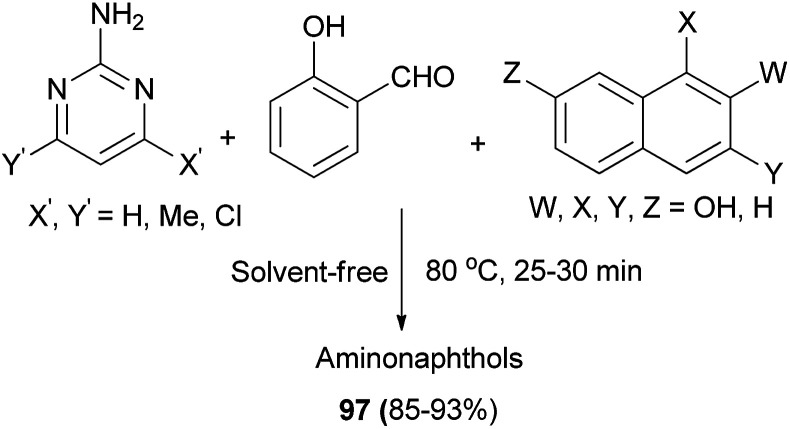
Green synthesis of aminonaphthols 97.

An efficient catalyst-free synthesis of Betti base derivatives *via* Mannich-type one-pot three-component condensation reaction of 2-aminopyrimidine derivatives, salicylaldehyde and naphthols (2-naphthol, 1-naphthol, 2,7-naphthalenediol and 2,3-naphthalenediol) under solvent-free conditions has been described. The reactions were carried out at 80 °C within 25–30 min, affording the desired aminonaphthols 97 in 85–93% yields (Scheme 57).^66^

**Scheme 58:**
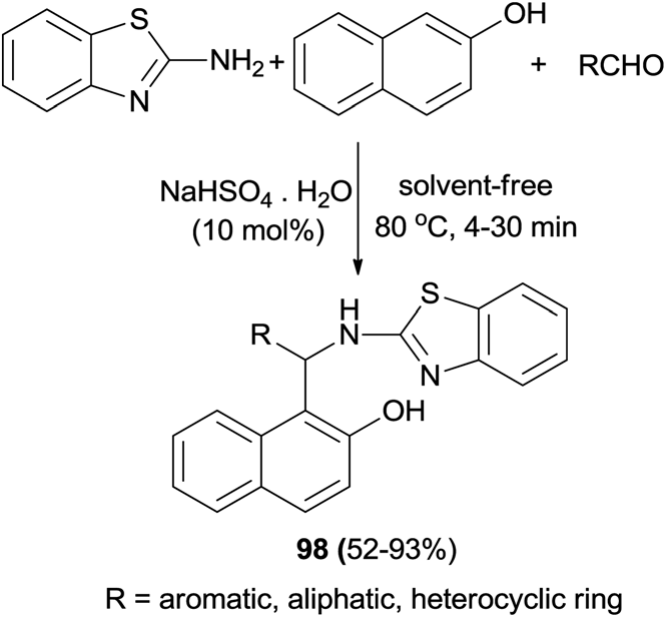
Synthesis of 1-(benzothiazolylamino)methyl-2-naphthols 98 catalyzed by NaHSO_4_·H_2_O.

Hosseinian *et al.*^67^ have reported that 1-(benzothiazolylamino)methyl-2-naphthol derivatives 98 are achieved from one-pot, three-component condensation of aldehydes, 2-naphthol, and 2-aminobenzothiazole in the presence of sodium hydrogen sulfate as an effective catalyst at 80 °C after 4–30 min. The reactions worked well with a variety of heterocyclic aldehydes, aliphatic aldehydes, and aryl aldehydes including those bearing electron-withdrawing and electron-donating groups, and the desired compounds were obtained in 52–93% yields (Scheme 58).

**Scheme 59:**
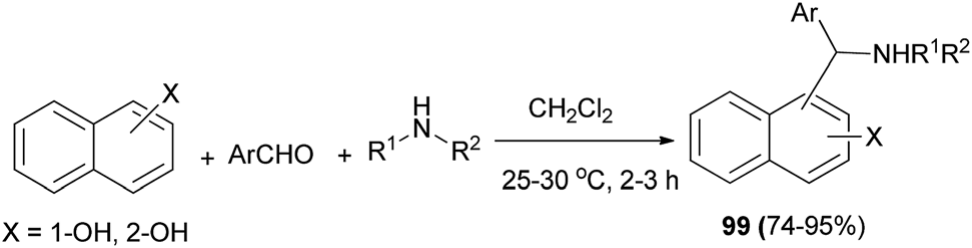
Synthesis of aminoalkylnaphthols 99 at room temperature in CH_2_Cl_2_.

A methodology has been developed for the multicomponent one-pot synthesis of aminoalkylnaphthols 99 in 74–95% yields in dichloromethane under catalyst-free conditions at room temperature within 2–3 h. Secondary amines such as piperidine, pyrrolidine, morpholine, *N*-methylpiperazine, and dimethylamine were used in the reaction and the yields of the desired products were moderate to excellent (Scheme 59).^68^

**Scheme 60:**
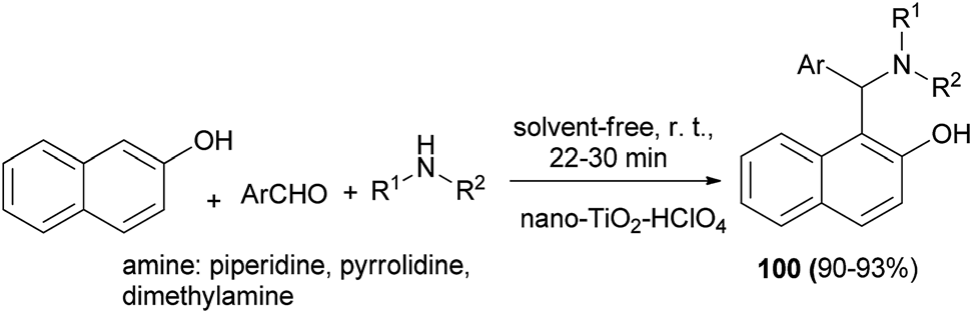
Nanocrystalline TiO_2_–HClO_4_-catalyzed synthesis of 1-(α-aminoalkyl)-2-naphthols 100.

Nanocrystalline TiO_2_–HClO_4_-catalyzed three-component preparation of 1-(α-aminoalkyl)-2-naphthols 100 in 90–93% yields under solvent-free condition at room temperature within 22–30 min has been reported by Shaterian *et al.* This white acidic heterogeneous catalyst is very stable under the reaction conditions and was reused several times without significant loss of activity (Scheme 60).^69^

**Scheme 61:**
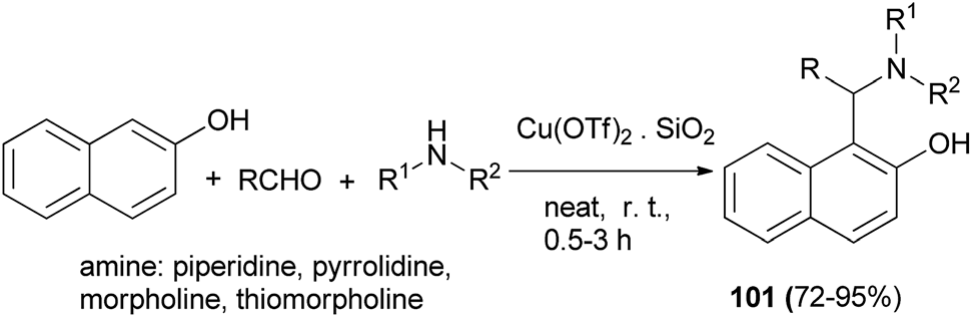
Supported copper triflate-catalyzed synthesis of Betti bases 101.

Jeong *et al.*^70^ described Cu(OTf)_2_·SiO_2_ (10 mol%)-catalyzed three-component coupling of aldehyde, 2-naphthol, and alicyclic amine to generate Betti bases 101 in 72–95% yields under neat conditions at room temperature to 40 °C after 0.5–3 h without additional co-catalyst or additive in air (Scheme 61).

**Scheme 62:**
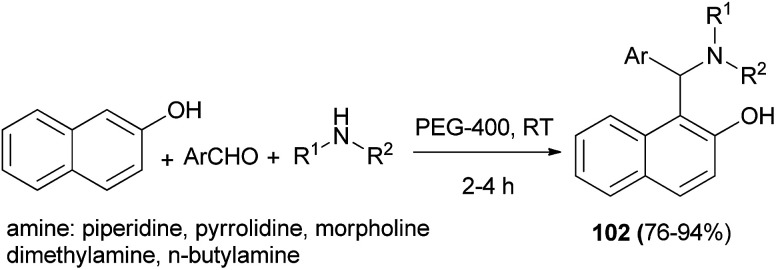
Catalyst-free synthesis of Betti bases 102 in PEG-400.

Betti bases 102 were obtained in 76–94% yields *via* the condensation reaction of aromatic aldehydes, secondary amines and 2-naphthol in PEG-400 as solvent in the absence of catalyst at room temperature after 2–4 h. In this reaction, aromatic aldehydes and heteroaromatic carbaldehydes worked satisfactorily, but the reaction with aromatic amines was not successful (Scheme 62).^71^

**Scheme 63:**
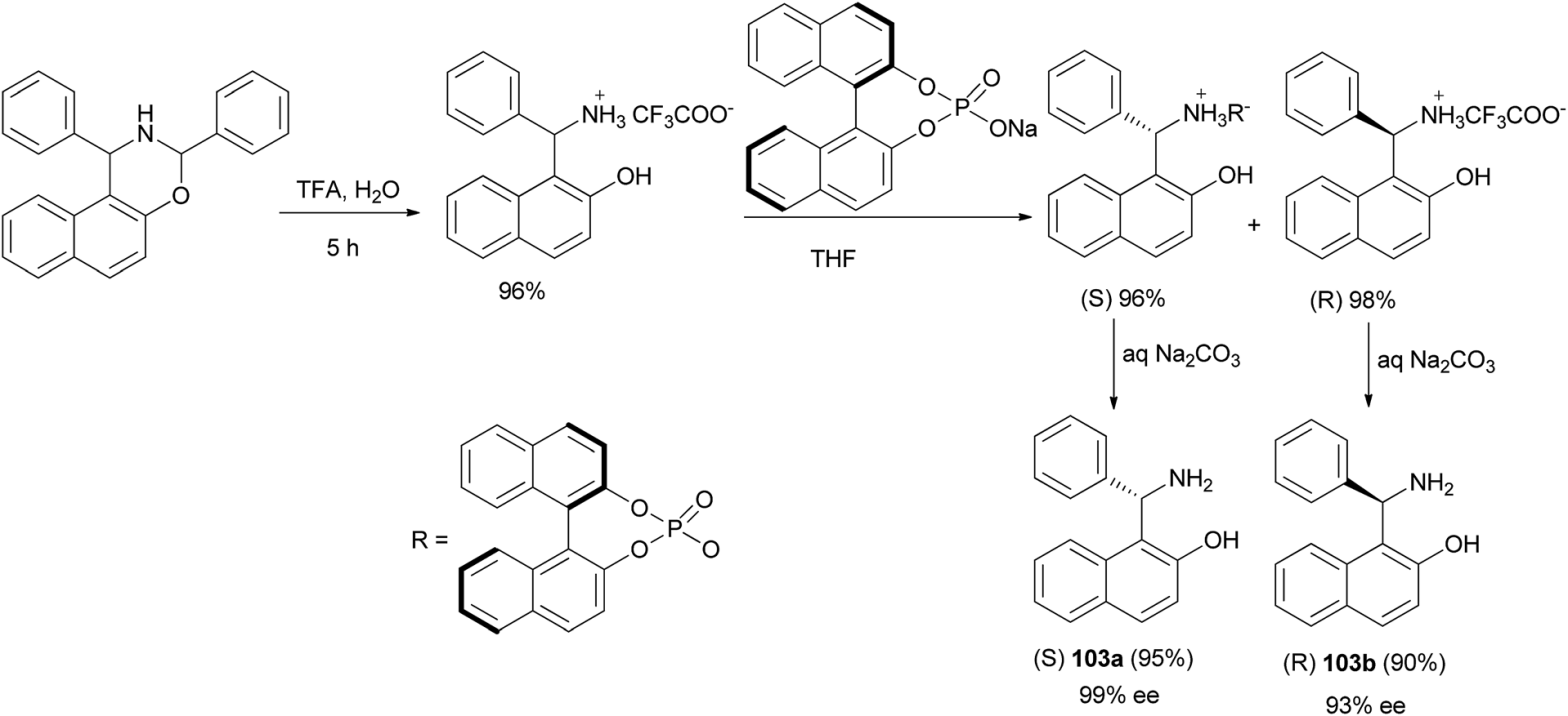
Resolution of Betti bases 103 by (*R*)-1,1′-binaphthalene-2,2′-diyl sodium phosphate.

Song *et al.*^72^ developed a new method for the synthesis of enantiomerically pure Betti bases 103. By using trifluoroacetic acid to replace the more traditionally used hydrochloric acid, the hydrolysis procedure used in the classical synthesis of racemic Betti base was carried out at 50 °C in CH_2_Cl_2_/H_2_O with an improved yield (up to 96%), which was followed by a new and efficient resolution using recyclable (*R*)-1,1′-binaphthalene-2,2′-diyl sodium phosphate to provide enantiomerically pure (*S*)-Betti base 103a in 95% yield with up to 99% ee and (*R*)-Betti base 103b in 93% yield with 90% ee in one resolution step (Scheme 63).

**Scheme 64:**
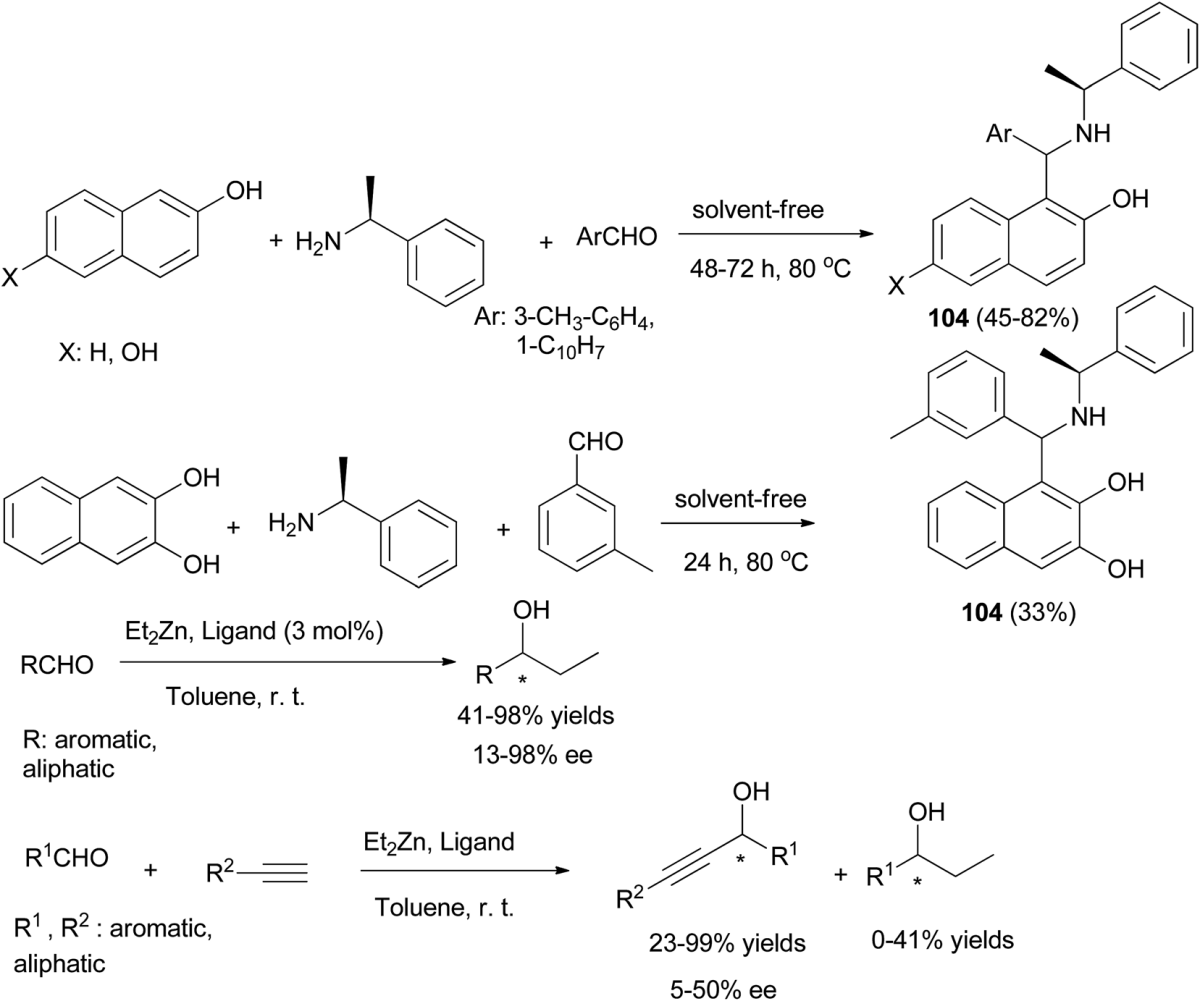
Diastereoselective synthesis of 1,3-aminonaphthols 104 and catalyzed addition of diethylzinc and alkynylzinc reagents to aldehydes.

A series of chiral aminonaphthols 104 has been synthesized diastereoselectively in 33–82% yields by applying a solvent-free ‘Betti-type’ condensation using 2,6- and 2,3-dihydroxynaphthalenes, 2-naphthol, (*S*)-phenylethylamine as a chiral auxiliary, and aldehydes at 80 °C after 24 and 72 h (Scheme 64). The major diastereomers (90–96%) formed could be isolated in pure form.^73^

**Scheme 65:**
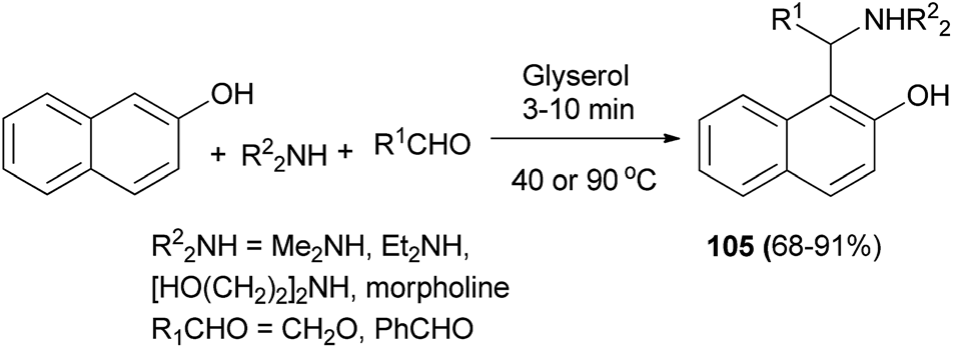
Betti reaction in glycerol.

These chiral aminonaphthols have been used as pre-catalysts for the addition of diethylzinc and alkynylzinc reagents to aldehydes in toluene at room temperature with enantioselectivities of up to 98% and 50% ee, respectively. Ganesan *et al.*^74^ described the Betti reaction in glycerol solvent. The expected Betti bases 105 were obtained at 40 or 90 °C after 3–10 min in 68–91% yields. The reaction works well for representative cyclic, acyclic and aliphatic aldehydes (Scheme 65).

**Scheme 66:**
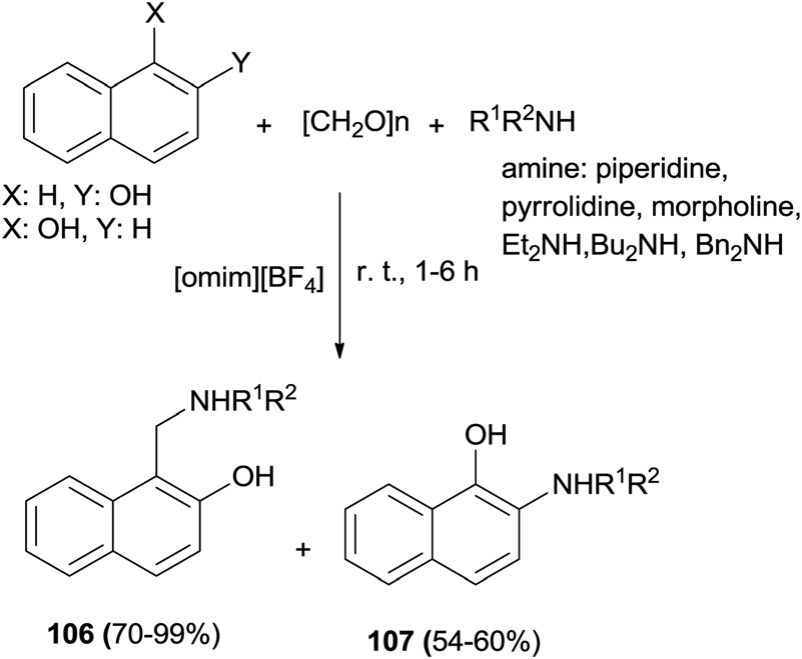
Aminomethylation of naphthols in the presence of [omim][BF_4_] ionic liquids.

Naimi-Jamal *et al.*^75^ published a simple procedure for the three-component one-pot aminomethylation of various aromatic electron-rich compounds using [omim][BF_4_] ionic liquids as the catalyst and solvent at room temperature. The process led to an efficient synthesis of Mannich bases 106 and 107 after 1–6 h in 54–99% yields under mild conditions using no additive or catalyst (Scheme 66).

**Scheme 67:**
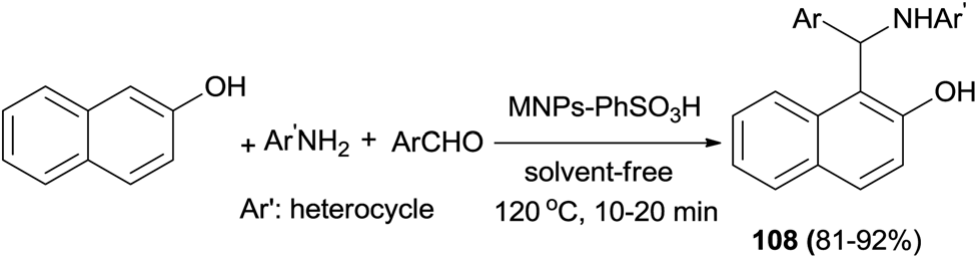
MNPs–PhSO_3_H-catalyzed synthesis of 1-aminoalkyl-2-naphthols 108.

Sulfanilic acid-functionalized silica-coated nano-Fe_3_O_4_ particles (MNPs–PhSO_3_H) as an efficient, reusable and magnetically separable catalyst has been studied for the solvent-free synthesis of 1-aminoalkyl-2-naphthols 108 at 120 °C. Treatment of a variety of aldehydes and 2-naphthol with heterocyclic amines afforded the corresponding 1-aminoalky-2-naphthol derivatives in 81–92% yields after 10–20 min (Scheme 67). The reaction with the usual aromatic amines such as aniline and *p*-toluidine afforded Schiff bases instead of the corresponding aminoalkylnaphthols.^76^

**Scheme 68:**
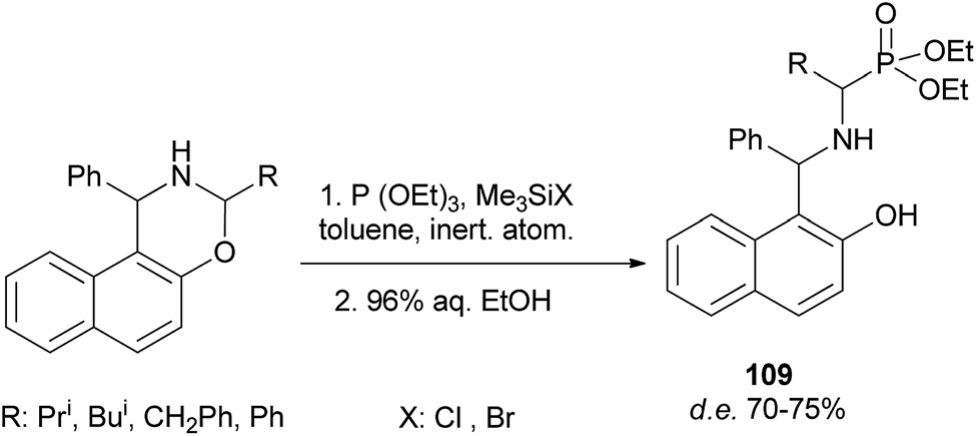
Diastereoselective synthesis of α-aminoalkylphosphonic acid derivatives 109.

A reaction of triethyl phosphite with 3-alkyl-1-phenylnaphthoxazines in the presence of halotrimethylsilanes in toluene with subsequent removal of the trimethylsilyl group by hydrolysis furnished diastereomeric α-aminoalkylphosphonic derivatives of Betti base 109. The highest diastereomeric excess was observed in the reaction with bromotrimethylsilane at low temperature (Scheme 68).^77^

**Scheme 69:**
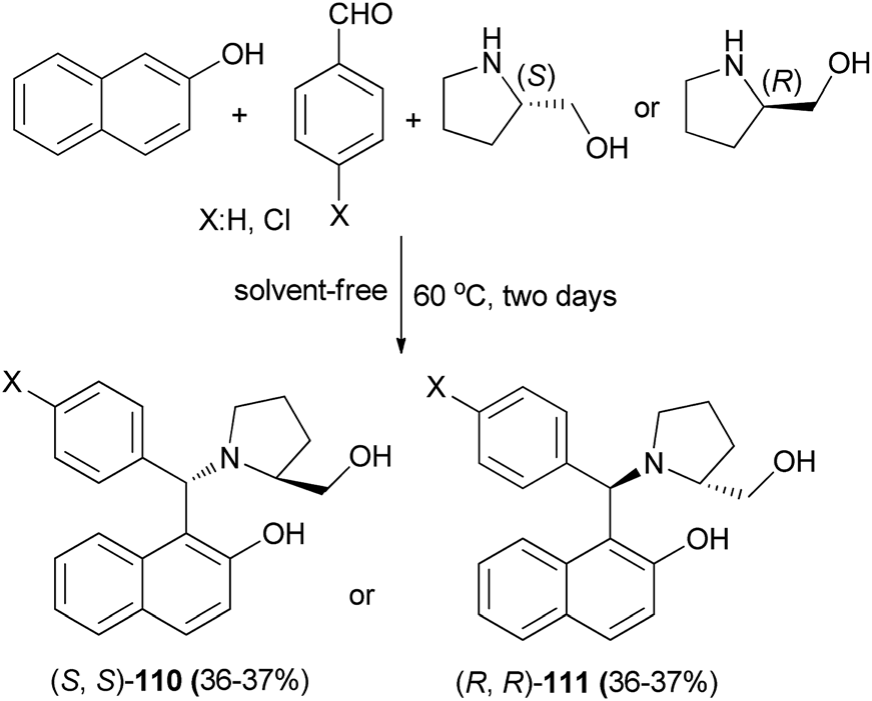
Synthesis of aminobenzylnaphthols 110 and 111.

Aminobenzylnaphthols 110 and 111 were synthesized in 36 and 37% yields *via* the reaction of 2-naphthol, arylaldehyde and (*S*)- or (*R*)-prolinol at 60 °C under solvent-free conditions for two days (Scheme 69). These aminobenzylnaphthols, synthesized from different components and thus having different structural features, were tested as anti-yeast agents inhibiting *Candida albicans*. The activity towards *C. albicans* of these prolinol derivatives was interesting and could represent a promising alternative to overcome the problem of strains resistant to the traditional antifungals.^78^

**Scheme 70:**
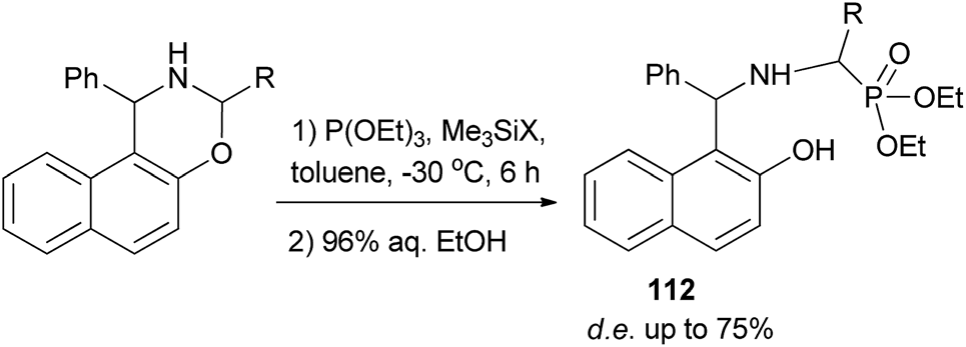
Diastereoselective synthesis of α-aminoalkylphosphonic acid derivatives of Betti base 112.

A reaction of triethyl phosphite with 3-alkyl-1-phenylnaphthoxazines in the presence of halotrimethylsilanes in toluene at −30 °C for 6 h with subsequent removal of the trimethylsilyl group by hydrolysis furnished diastereomeric α-aminoalkylphosphonic derivatives of Betti base 112 with high diastereoselectivity (de up to 75%) (Scheme 70).^79^

**Scheme 71:**
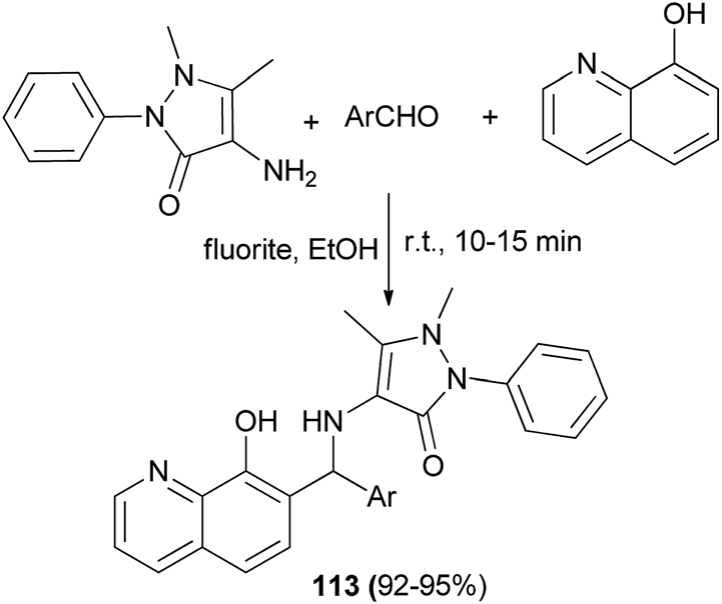
Synthesis of 4-aminoantipyrine derivatives 113 derived from Betti-type reaction.

4-Aminoantipyrine derivatives 113 were achieved in 92–95% yields by the condensation of aromatic aldehyde, 4-aminoantipyrine, and 8-hydroxyquinoline in the presence of fluorite as catalyst in ethanol at room temperature for 10–15 min (Scheme 71). All derivatives showed *in vivo* and *in vitro* anti-inflammatory and anthelmintic activities against reference drugs diclofenac and albendazole.^80^

**Scheme 72:**
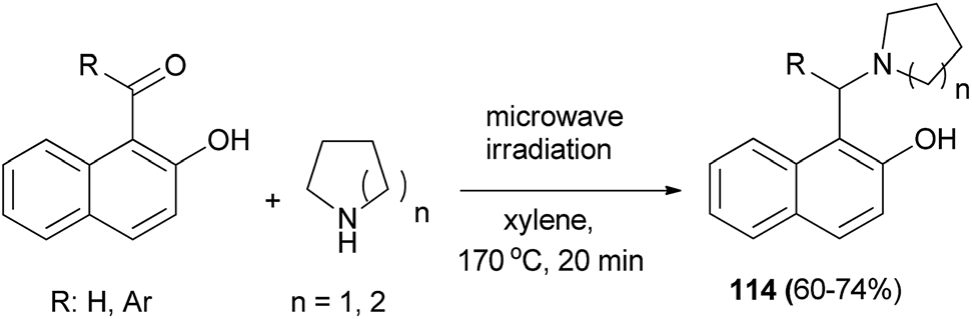
Synthesis of Betti bases 114.

Jana *et al.*^81^ reported the reaction of 2-hydroxynaphthaldehyde with two equivalents of pyrrolidine in xylene under microwave irradiation at 170 °C within 20 min, giving 2-hydroxy-1-naphthylmethylamine (114) as the major product. Similarly, various aldehydes and ketones were reacted with different cyclic saturated amines producing structurally diverse mono- or di-arylmethylamines 114 in 60–74% yields (Scheme 72).

**Scheme 73:**
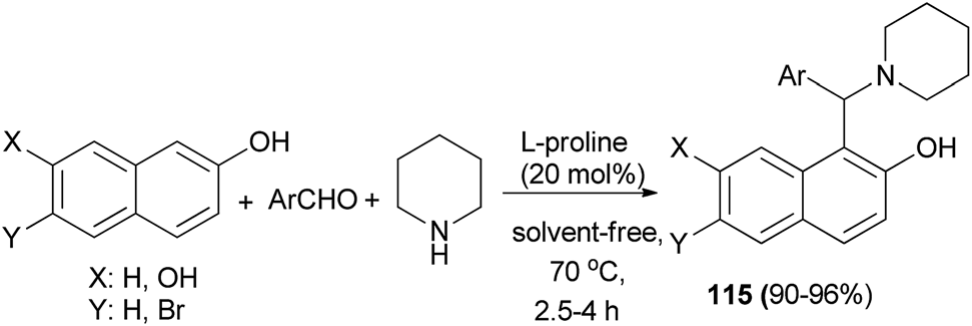
l-Proline-catalyzed synthesis of Betti bases 115.

The direct three-component modified Mannich reaction *via* condensation of aromatic aldehydes, 2-naphthol or 2,7-naphthalendiol and piperidine to generate Betti bases 115 in 90–96% yields has been carried out over l-proline (20 mol%) with high efficiency under solvent-free conditions at 70 °C within 2.5–4 h (Scheme 73).^82^

**Scheme 74:**
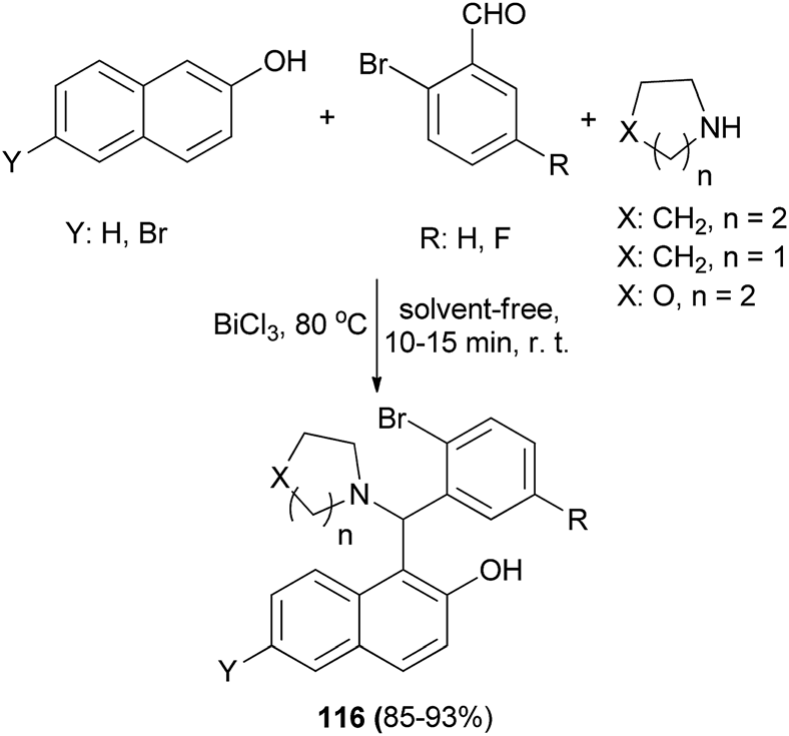
BiCl_3_-catalyzed synthesis of Betti bases 116.

BiCl_3_ (7.5 mol%) as an efficient catalyst has been studied for the synthesis of aminonaphthols 116*via* the reaction of naphthols, 2-bromobenzaldehydes and cyclic secondary amines at 80 °C without solvent within 10–15 min in 85–93% yields (Scheme 74).^83^

**Scheme 75:**
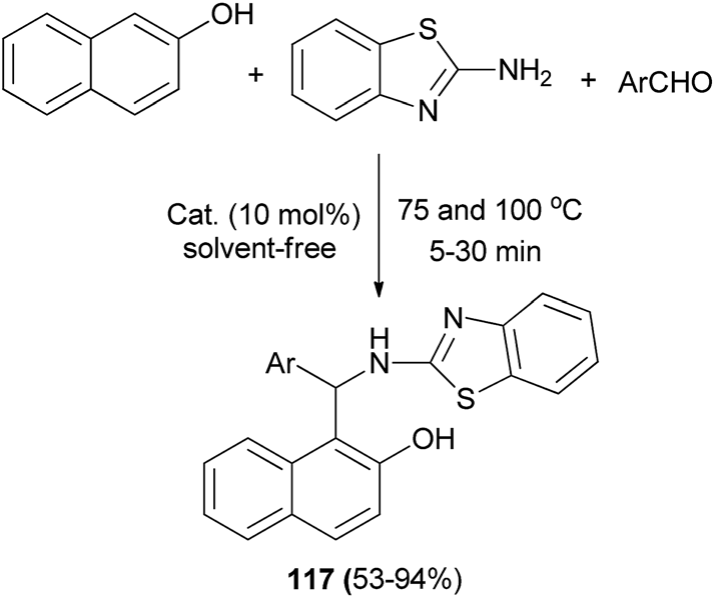
Synthesis of 1-(benzothiazolylamino)methyl-2-naphthols 117 catalyzed by [(CH_2_)_3_SO_3_HMIM][HSO_4_].

A Brønsted acidic ionic liquid, [(CH_2_)_3_SO_3_HMIM][HSO_4_], as an efficient catalyst has been reported for the synthesis of 1-(benzothiazolylamino)methyl-2-naphthols 117 at 100 °C (or 75 °C for heterocyclic aldehydes) under solvent-free condition within 5–30 min. A wide range of aldehydes readily undergo condensation with 2-naphthol and 2-aminobenzothiazole to afford the desired products in 53–94% yields (Scheme 75).^84^

**Scheme 76:**
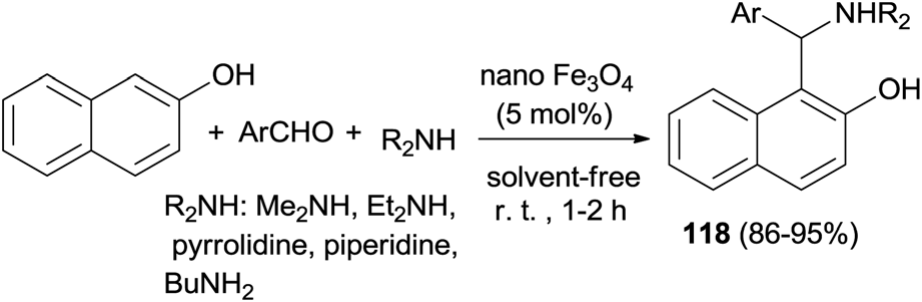
Synthesis of Betti bases 118, catalyzed by nano Fe_3_O_4_ at room temperature.

Heravi *et al.*^85^ have shown solventless synthesis of Betti bases 1-(α-aminoalkyl)naphthols 118 catalyzed by Fe_3_O_4_ nanoparticles (5 mol%) at room temperature. All the aromatic aldehydes and cyclic and acyclic amines reacted with 2-naphthol almost equally well to afford the Betti bases in 86–95% yields after 1–2 h. The reaction was not successful with aromatic amines, which might be due to their reduced nucleophilicity (Scheme 76).

**Scheme 77:**
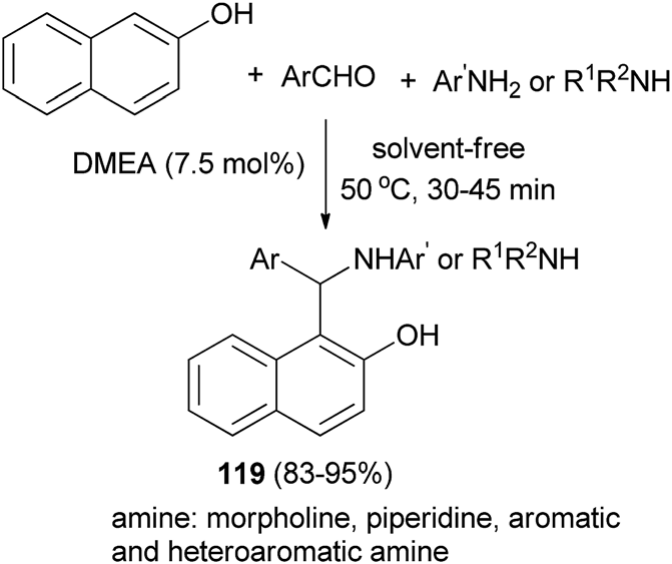
Synthesis of arylaminonaphthols 119 using DMEA under solvent-free conditions.

Shahrisa *et al.*^86^ have obtained arylaminonaphthols 119 from the condensation of 2-naphthol, aldehydes, and arylamines in the presence of *N*,*N*-dimethylethanolamine (7.5 mol%) as an organocatalyst at 50 °C under solvent-free conditions. The reaction of aromatic and heteroaromatic aldehydes with piperidine, morpholine, aromatic amines and heteroarylamines in the presence of catalytic amounts of DMEA also afforded desired products after 30–45 min in 83–95% yields (Scheme 77).

**Scheme 78:**
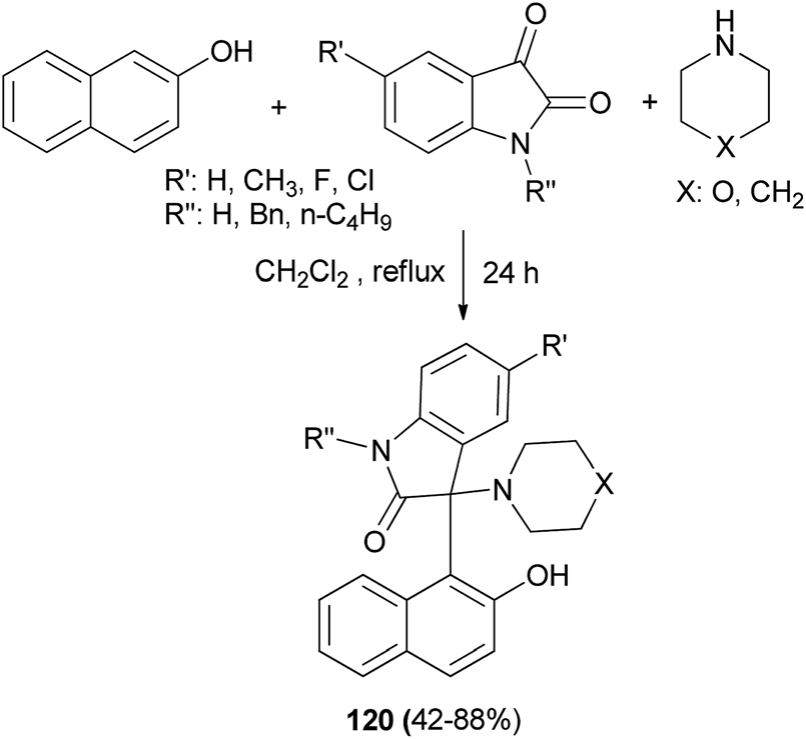
Synthesis of Betti bases 120*via* three-component reaction of 2-naphthol, cyclic amines and isatins.

The first example of incorporating the oxindole moiety into Betti bases has been reported by Yan *et al.*^87^ The new type of Betti bases 120 were conveniently synthesized in 42–88% yields from the three-component reaction of 2-naphthol, isatins and cyclic amines, such as piperidine or morpholine, in CH_2_Cl_2_ at reflux within 24 h without any other catalyst (Scheme 78). Other secondary amines, such as pyrrolidine, dimethylamine, diethylamine, and di(*n*-propyl)amine, and α-naphthol, resorcinol, and pyrogallol did not afford the expected products under similar reaction conditions.

**Scheme 79:**
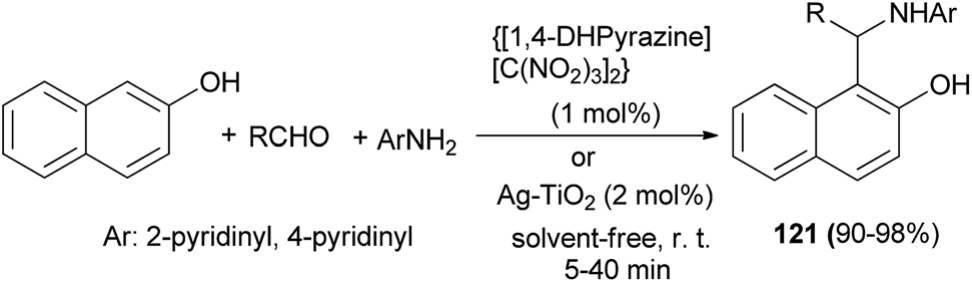
Synthesis of 1-(α-aminoalkyl)-2-naphthols 121.

Pyrazine-1,4-diium trinitromethanide {[1,4-DHPyrazine][C(NO_2_)_3_]_2_} as a green and novel nanostructured molten salt catalyzed the synthesis of new 1-(α-aminoalkyl)-2-naphthol derivatives 121*via* the reaction between 2- or 4-aminopyridine, aromatic aldehydes and 2-naphthol at room temperature under solvent-free conditions in comparison with Ag–TiO_2_ nanocomposite. The reactions with {[1,4-DHPyrazine][C(NO_2_)_3_]_2_} (1 mol%) and Ag–TiO_2_ (2 mol%) afforded the desired products after 5–25 min and 10–40 min in 92–98% and 90–95% yields, respectively (Scheme 79).^88^

**Scheme 80:**
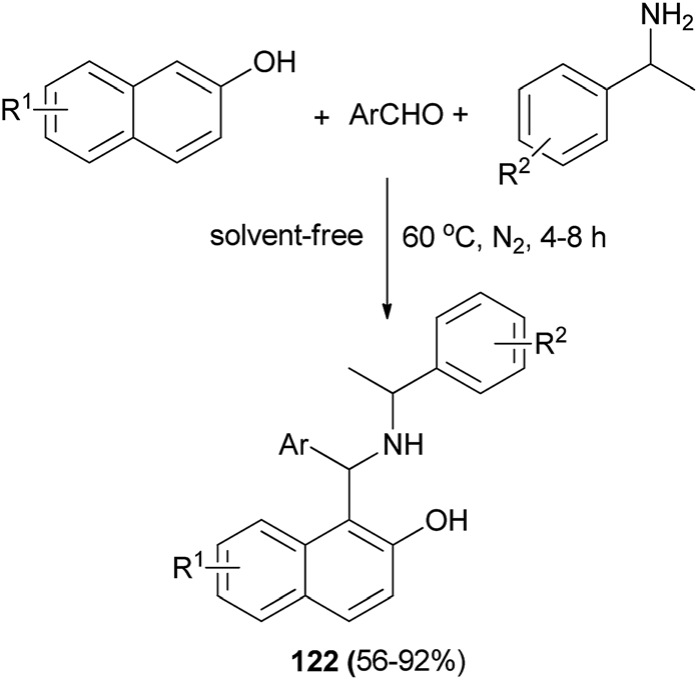
Synthesis of Betti base derivatives 122.

Xu *et al.*^89^ demonstrated that Betti bases 122 can be easily synthesized without a solvent by condensation of 2-naphthols, aromatic aldehydes, and 1-phenylethylamines at 60 °C for 4–8 h under nitrogen atmosphere in 56–92% yields (Scheme 80). Also, these compounds can be converted to γ-amino alcohols and downstream pyrrolidine derivatives by highly diastereoselective [1,2]-Wittig rearrangement and intramolecular cyclization with perfect chirality transfer (up to >99.9% de) and in good yields.

**Scheme 81:**
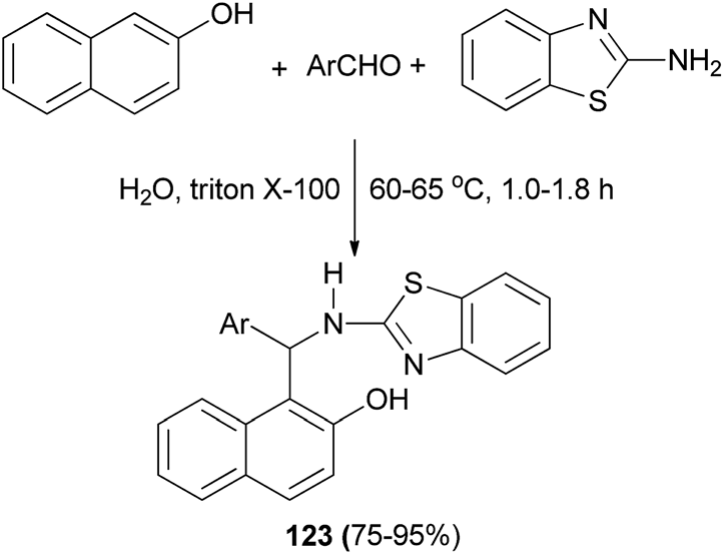
Triton X-100-catalyzed synthesis of 2-aminobenzothiazolomethylnaphthol derivatives 123.

An efficient method has been described for the synthesis of 2-aminobenzothiazolomethylnaphthol derivatives 123 by one-pot three-component reaction of aldehydes, 2-naphthol, and 2-aminobenzothiazole using Triton X-100 as a catalyst in water at 60–65 °C within 1.0–1.8 h in 75–95% yields (Scheme 81). It was shown that the synthesized derivatives demonstrated good antimicrobial activity against Gram-positive and Gram-negative bacteria and antifungal activity against fungal strains such as *Aspergillus niger*, *Aspergillus fumigatus*, and *Aspergillus flavus*.^90^

**Scheme 82:**
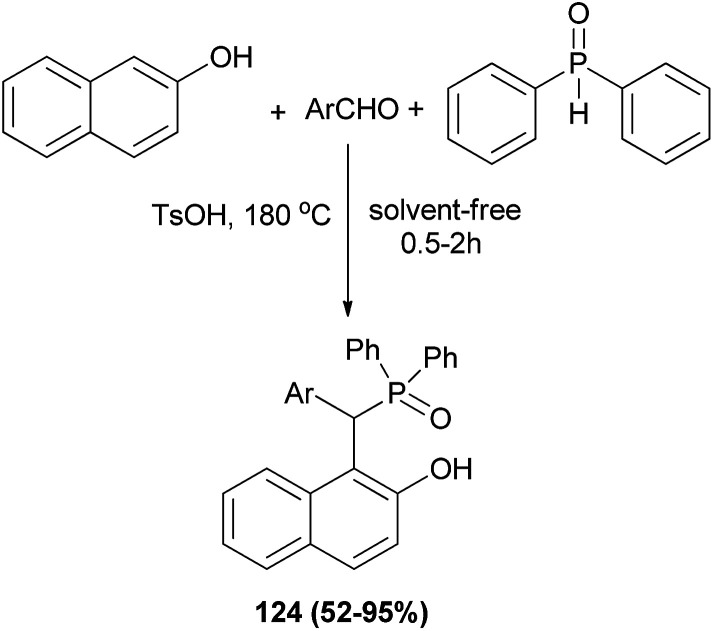
Synthesis of bifunctional phosphorus Betti bases 124.

Wu *et al.*^91^ demonstrated for the first time a straightforward and one-pot strategy to synthesize the bifunctional phosphorus Betti bases 124 in 52–95% yields under solvent-free conditions at 180 °C within 0.5–2 h *via* the condensation of 2-naphthol, arylaldehydes and diphenylphosphine oxide in the presence of *p*-toluenesulfonic acid (Scheme 82). The procedure worked well with almost all the aldehydes.

**Scheme 83:**
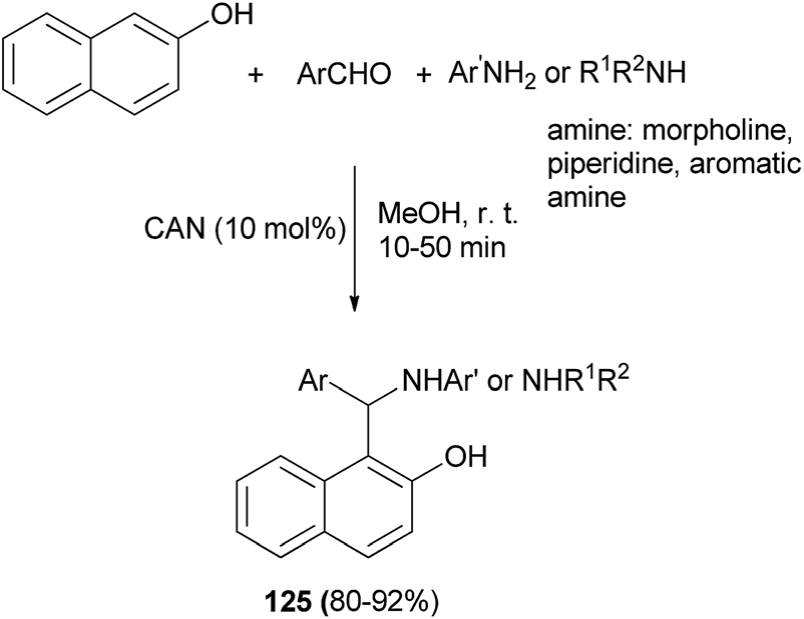
Synthesis of Betti bases 125 catalyzed by CAN.

Abdel Hameed *et al.*^92^ reported an efficient multicomponent, one-pot synthesis of Betti bases 125 catalyzed by cerium(iv) ammonium nitrate (CAN) (10 mol%) in MeOH as solvent at ambient temperature. The reaction worked well for representative anilines, morpholine, piperidine and aromatic aldehydes, and led to the formation of the desired products after 10–50 min in 80–92% yields (Scheme 83).

**Scheme 84:**
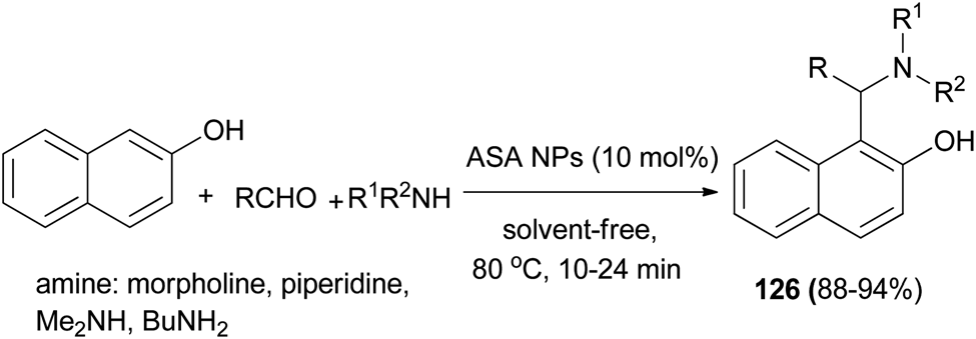
Synthesis of 1-(α-aminoalkyl)-2-naphthol derivatives 126 in the presence of ASA NPs.

The work of Nasr-Esfahani *et al.*^93^ demonstrated that various Betti bases 126 could be synthesized in one-pot three-component condensation of aldehydes, 2-naphthol, and cyclic and acyclic amines in the presence of aluminatesulfonic acid nanoparticles (ASA NPs) (10 mol%) as recoverable catalyst at 80 °C under solvent-free conditions. Aromatic aldehydes containing either electron-donating or electron-withdrawing groups reacted successfully and gave the products within 10–24 min in 88–94% yields (Scheme 84).

**Scheme 85:**
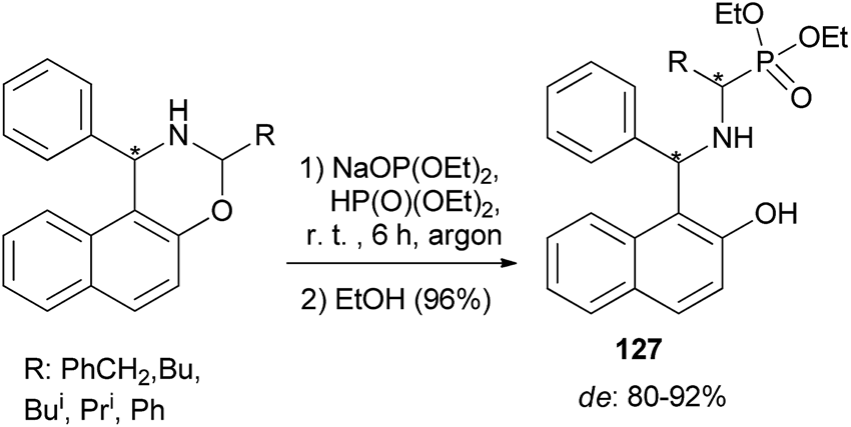
Diastereoselective synthesis of α-aminophosphonates 127.

Sadkova *et al.*^94^ developed a diastereoselective (de 80–92%) synthesis of α-aminophosphonates 127 by reaction of diethyl phosphite sodium salt with 3-*R*-1-phenyl-2,3-dihydro-1*H*-naphth[1,2-*e*]-[1,3]oxazines being the products of aminoacetalization of aldehydes with 1-(α-aminobenzyl)-2-naphthol (Betti base). The reaction mixtures were vigorously stirred for 6 h at room temperature, followed by the addition of 96% ethanol (Scheme 85).

**Scheme 86:**
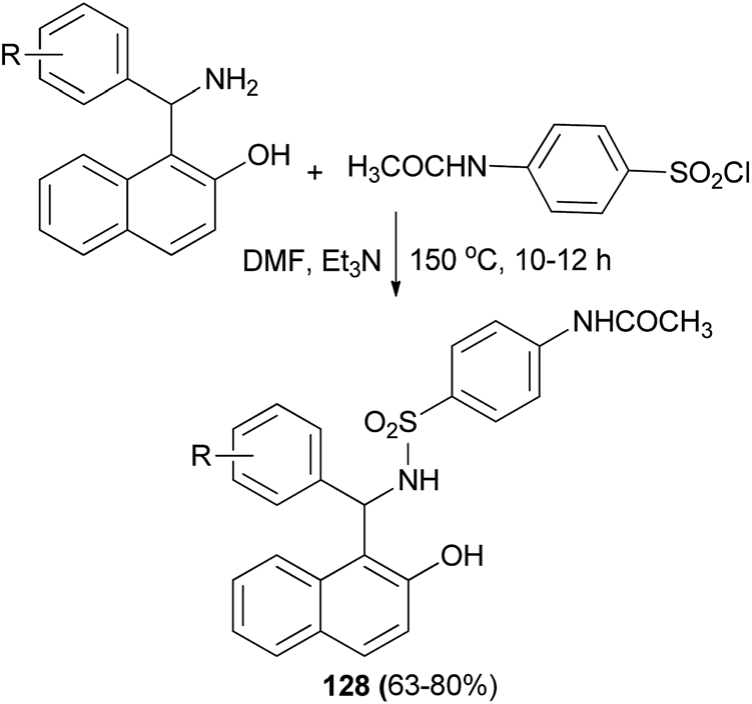
Synthesis of *N*-(4-(*N*-((2-hydroxynaphthelen-1-yl)(phenyl)methyl)sulfamoyl)phenyl)acetamide derivatives 128.

Meshram *et al.*^95^ synthesized a series of sulfonamides fused with Betti bases 128 in 63–80% yields by the reaction of substituted 1-(amino(phenyl)methyl)naphthalen-2-ol and acetamidobenzenesulfonyl chloride in DMF and triethylamine at 150 °C for 10–12 hours (Scheme 86). On the basis of the results obtained from docking studies, some of the synthesized sulfonamide derivatives 128 might show significant anticancer activity by inhibiting DNA topoisomerase II.

**Scheme 87:**
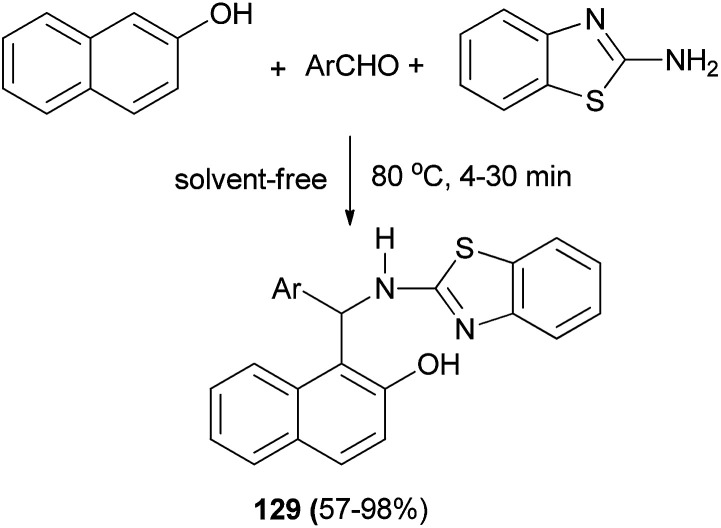
Synthesis of compounds 129 catalyzed by oxalic acid.

A green protocol for one-pot three-component synthesis of 1-(benzothiazolylamino)methyl-2-naphthols 129*via* the reaction of aromatic aldehydes, 2-naphthol and 2-aminobenzothiazole catalyzed by oxalic acid (20 mol%) under thermal and solvent-free conditions is described by Maghsoodlou *et al.* The reaction was carried out under solvent-free condition at 80 °C for 4–30 min to give the corresponding products in 57–98% yields (Scheme 87).^96^

**Scheme 88:**
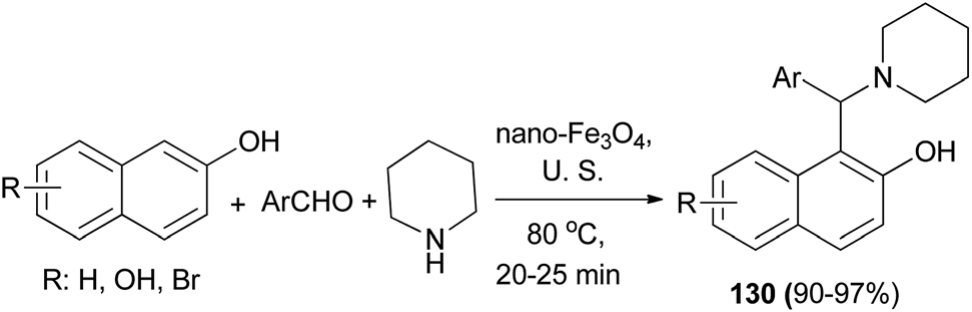
Fe_3_O_4_ is an efficient and robust catalyst for the one-pot synthesis of Betti bases 130.

The direct three-component reaction *via* condensation of aldehydes, 2-naphthols and piperidine has been reported, generating 1-(aryl(piperidin-1-yl)methyl)naphthalene-2-ol derivatives 130 over Fe_3_O_4_ magnetic nanoparticles with high efficiency (90–97% yields) under ultrasound irradiation (frequency of 40 kHz and an input power of 600 W) and solvent-free condition at 80 °C for 20–25 min (Scheme 88).^97^

**Scheme 89:**
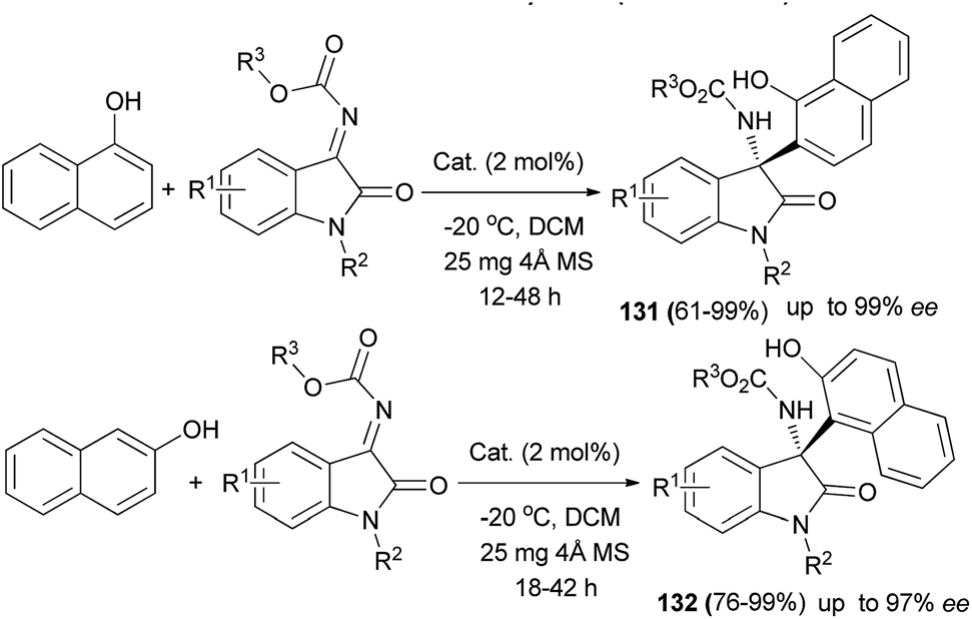
Reaction scope of aza-Friedel–Crafts reaction of naphthols to isatin ketimines.

Aza-Friedel–Crafts reaction of *N*-alkoxycarbonyl isatin ketimines with naphthols using a new 2-adamantyl-substituted quinine-derived squaramide catalyst (2 mol%) and 25 mg 4 Å molecular sieve in CH_2_Cl_2_ at −20 °C for 12–48 h afforded chiral tetrasubstituted 3-amino-2-oxindoles 131 and 132 with excellent enantioselectivity up to greater than 99% ee and in 61–99% yields (Scheme 89).^98^

**Scheme 90:**
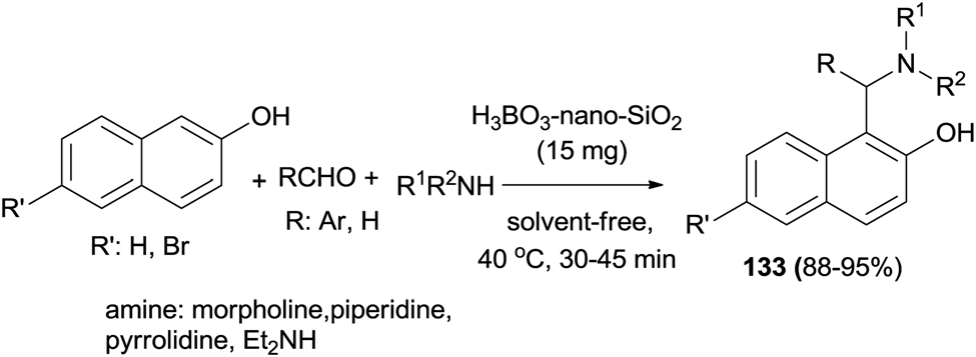
One-pot synthesis of aminonaphthols 133, using nano-SiO_2_–H_3_BO_3_ as a catalyst.

Nano-SiO_2_–H_3_BO_3_ was introduced as an environmentally benign and recyclable heterogeneous catalyst for the synthesis of aminonaphthols 133 at 40 °C under solvent-free conditions after 30–45 min in 88–95% yields without an additional co-catalyst or additive in air by Teimuri-Mofrad *et al.*^99^ (Scheme 90).

**Scheme 91:**
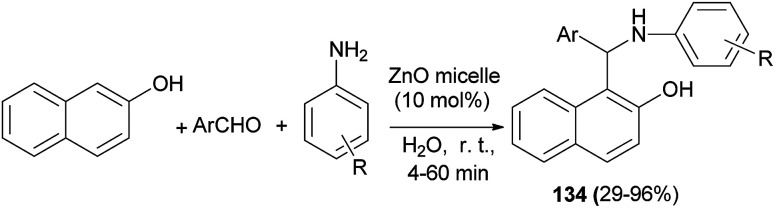
Synthesis of Betti base derivatives 134 catalyzed by reverse ZnO nanomicelles.

Pei *et al.*^100^ reported a highly efficient one-pot three-component Betti reaction using reverse ZnO micelles as a recoverable and reusable catalyst. Reactions were conducted with 2-naphthol, aromatic aldehydes, amines and reverse ZnO nanomicelles (10 mol%) in water at room temperature within 4–60 min, affording Betti base derivatives 134 in 29–96% yields (Scheme 91).

**Scheme 92:**
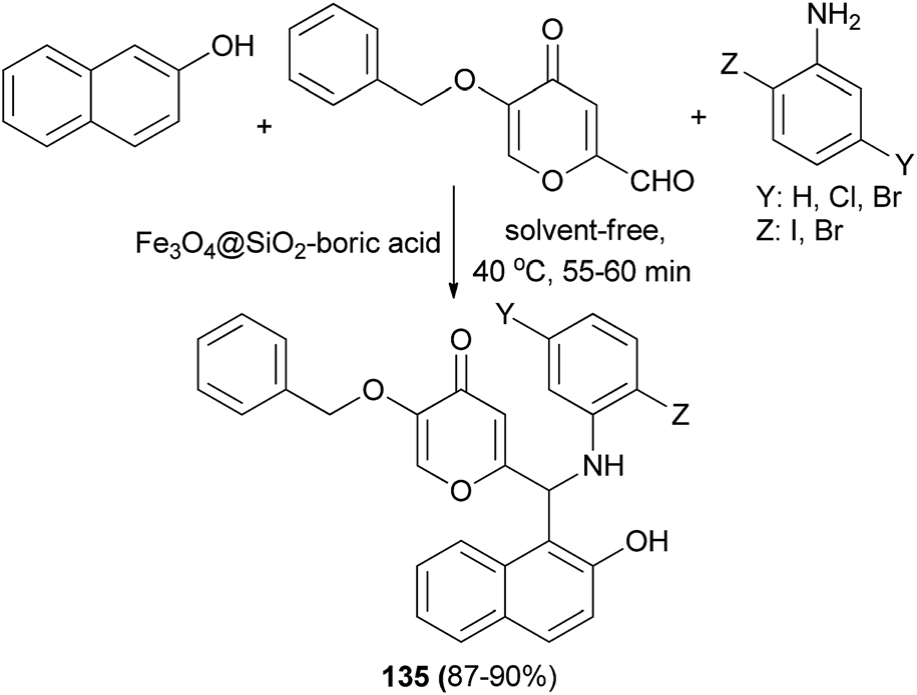
Synthesis of Betti bases 135 based on kojic acid derivatives.

Novel Betti bases 135 based on kojic acid derivatives have been synthesized by coupling 2-naphthol, aniline derivatives and kojic aldehyde in the presence of Fe_3_O_4_@SiO_2_–boric acid nanocatalyst at 40 °C min under solvent-free condition (Scheme 92). The reactions were carried out efficiently within 55–60 min and the desired products obtained in 87–90% yields.^101^

**Scheme 93:**
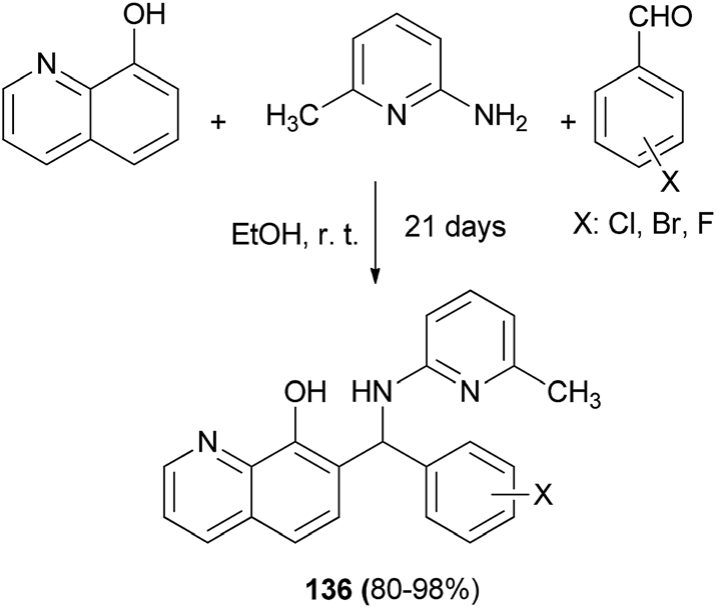
Synthesis of Betti bases 136 by a modified Betti reaction.

Georgieva *et al.*^102^ synthesized several Betti bases 136 by a modified Betti reaction *via* the condensation reaction of primary heterocyclic amine, aromatic aldehydes, 8-quinolinol and halogeno-substituted aromatic aldehydes. The reaction mixtures were allowed to stand for 21 days in absolute ethanol at room temperature in a closed flask affording the corresponding products in yields of 80–98% (Scheme 93).

**Scheme 94:**
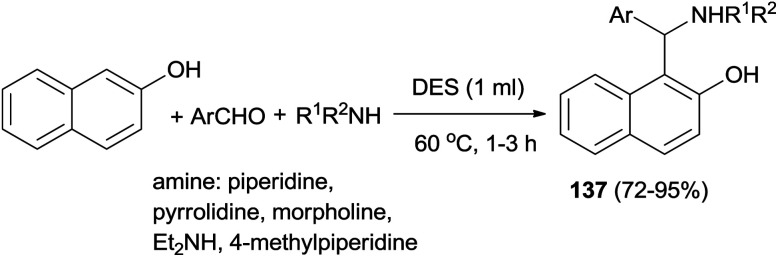
Synthesis of 1-aminoalkyl-2-naphthols 137 in deep eutectic solvent (DES).

Synthesis of aminoalkylnaphthols 137 from aldehydes, 2-naphthol, and secondary amines in deep eutectic solvent (DES) based on urea and choline chloride has been developed. A broad range of aminoalkylnaphthols can be obtained at 60 °C within 1–3 h in 72–95% yields in biodegradable choline chloride-based DES (urea–ChCl (2 : 1)). Heteroaromatic as well as sterically hindered aromatic aldehydes also gave good product yields with aliphatic aldehydes failing to produce corresponding Betti bases (Scheme 94).^103^

**Scheme 95:**
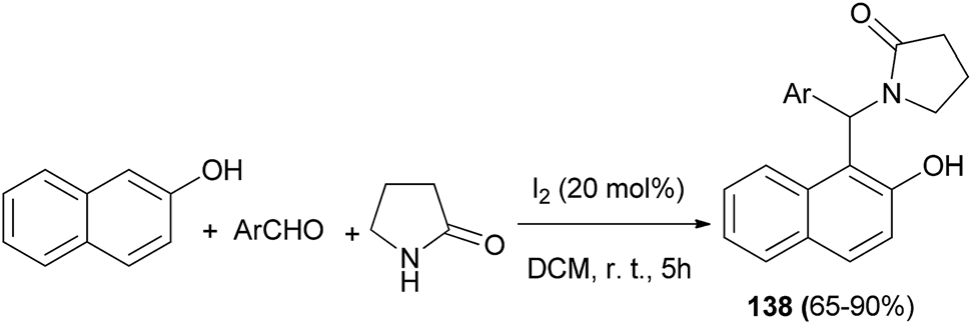
Iodine-catalyzed synthesis of 1-((2-hydroxynapthalen-1-yl)(aryl))pyrrolidin-2-ones 138.

Iyer *et al.*^104^ achieved a series of 1-((2-hydroxynaphthalen-1-yl)(phenyl)(methyl))pyrrolidin-2-one derivatives 138 in 65–90% yields by an efficient iodine (20 mol%)-catalyzed domino reaction involving various aromatic aldehydes, 2-pyrrolidinone and 2-naphthol in CH_2_Cl_2_ at room temperature after 5 h (Scheme 95).

Subsequently they were evaluated for cytotoxicity against breast cancer (MCF-7) and colon cancer (HCT116) cell lines. Regarding the cytotoxicity, the relative inhibition activity was notably found to be moderate to high in MCF-7 cell line. Also, they were docked into the active site of phosphoinositide 3-kinase (PI3K) (PDB ID: 4JPS) which is a crucial regulator of apoptosis or programmed cell death. Results showed that the hydrophobic interactions in the binding pockets of PI3K exploited the affinity of the most favorable binding ligands.

**Scheme 96:**
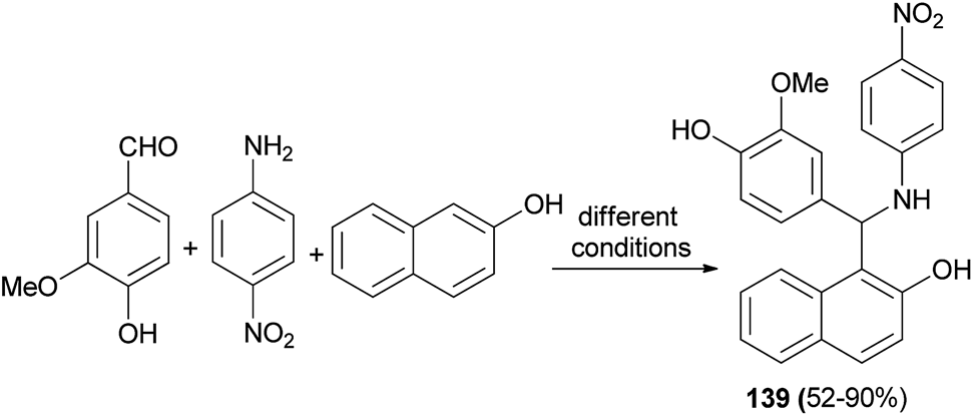
Synthesis of vanillin-based aminoalkylnaphthol 139.

Cost-effective green chemical methods are reported for the one-pot multicomponent solventless synthesis of 1-aminoalkyl-2-naphthol 139 in 52–90% yields by the reaction of vanillin, 2-naphthol and 4-nitroaniline in the presence of tannic acid as a Lewis acid catalyst. A mixture was stirred by following various green protocols such as oil bath at 120–125 °C for 10–15 min, microwave irradiation at 100 W for 1–4 min, hot plate with magnetic stirrer at about 120–125 °C for 20–25 min, grindstone method for 10–15 min and finally conventional heating method with a few drops of methanol at reflux for 60–90 min (Scheme 96). Antibacterial activity of the synthesized compound against *Bacillus subtilis* was tested by zone inhibition method, showing negligible inhibition against the growth of this bacterium.^105^

**Scheme 97:**
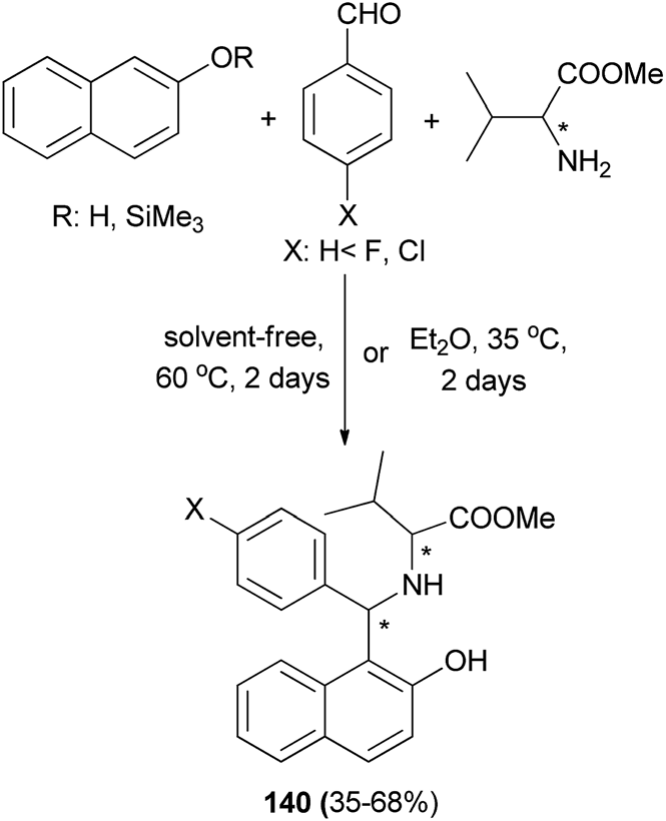
Synthesis of aminobenzylnaphthol 140 bearing two stereogenic centers.

Cardellicchio and Capozzi^106^ succeeded in the preparation of aminobenzylnaphthol 140 bearing two stereogenic centers from the condensation of 2-naphthol, arylaldehydes and valine methyl ester at 60 °C under solvent-free condition for two days or in Et_2_O at 35 °C for two days in 35–68% yields (Scheme 97).

**Scheme 98:**
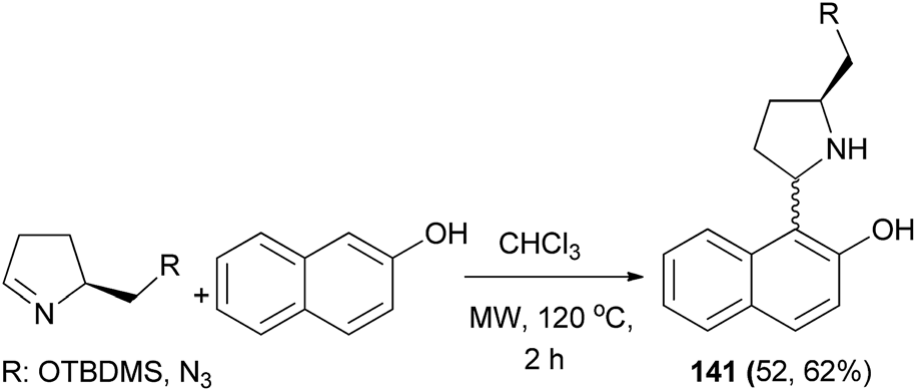
Synthesis of Betti bases 141.

The Betti bases 141 in 52 and 62% yields were obtained from the reaction of (*S*)-5-{[(*tert*-butyldimethylsilyl)oxy]methyl}pyrrolidin-2-one or (*S*)-5-(azidomethyl)pyrrolidin-2-one and 2-naphthol in dry CHCl_3_ under microwave irradiation at 120 °C for 2 h (Scheme 98).^107^

**Scheme 99:**
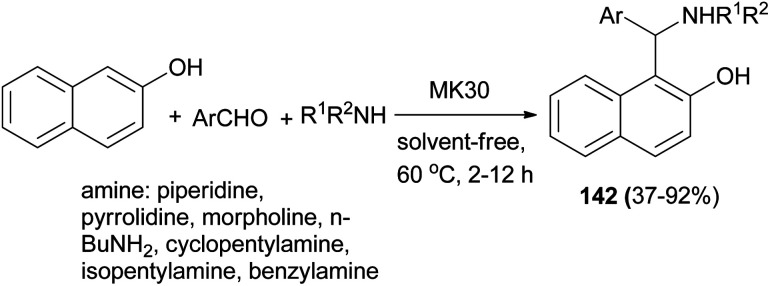
MK30-catalyzed synthesis of Betti bases 142.

Bosica *et al.*^108^ reported that the reaction of 2-naphthol, aromatic aldehydes and amines in the presence of montmorillonite K30 as a heterogeneous catalyst at 60 °C under neat conditions yielded aminocycloalkylnaphthols 142 after 2–12 h. The catalyst is fully recoverable and recyclable for up to 5 runs. Furthermore, it could catalyze reactions involving both secondary and primary aliphatic amines to give products usually in 37–92% yields (Scheme 99).

**Scheme 100:**
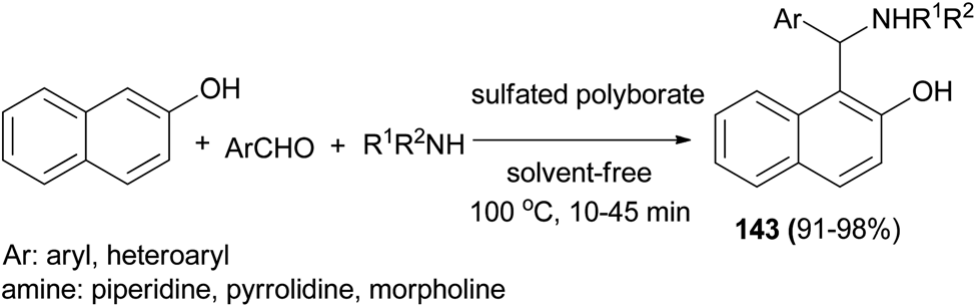
Solvent-free synthesis of Betti bases 143 catalyzed by sulfated polyborate.

Chaturbhuj *et al.*^109^ succeeded in the preparation of 1-aminoalkyl-2-phenol Betti bases 143 from one-pot three-component condensations of aldehyde, 2-naphthol, and morpholine in the presence of sulfated polyborate catalyst, under a solvent-free condition at 100 °C. Various aromatic/heteroaromatic aldehydes and cyclic amines (morpholine, piperidine, and pyrrolidine) reacted well and afforded higher yields (91–98%) of Betti base in shorter reaction time (10–45 min) (Scheme 100).

**Scheme 101:**
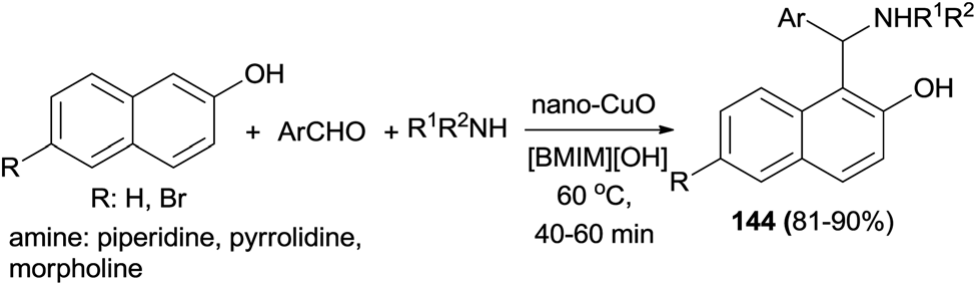
Synthesis of Betti bases 144 catalyzed by nano-CuO–ionic liquid.

Synthesis of Betti base derivatives 144 catalyzed by nano-CuO–ionic liquid has been reported from the reaction of 2-naphthols, aromatic aldehydes and cyclic amines in [BMIM][OH] at 60 °C. The desired products were obtained in a short period of time (40–60 min) in good yields (81–90%) (Scheme 101). All of the synthesized aminonaphthol derivatives 144 exhibit good inhibitory effects toward mild steel corrosion with inhibition efficiencies in the range of 84–95%. Therefore, these compounds can be practically used as effective corrosion inhibitors for acid pickling applications.^110^

**Scheme 102:**
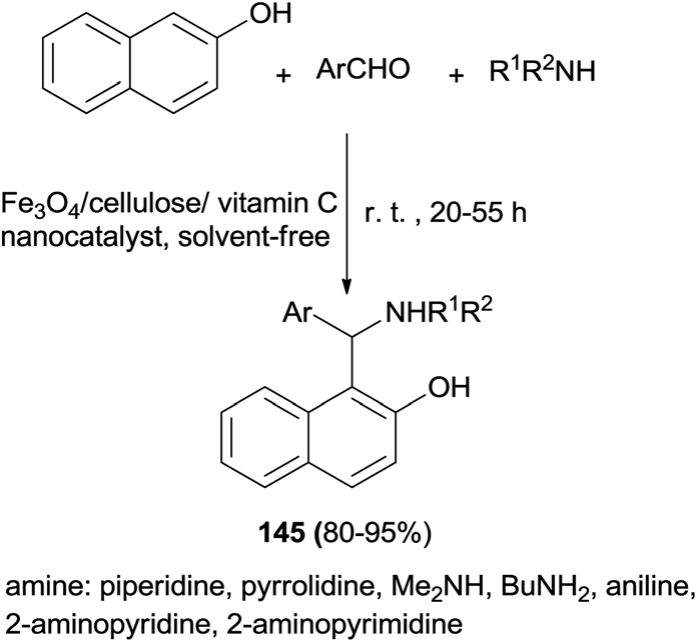
Nanocomposite of Fe_3_O_4_/cellulose/vitamin C as a new biopolymer catalyst for synthesis of 1-(α-aminoalkyl)naphthols 145.

Janati^111^ introduced a nanocomposite of Fe_3_O_4_/cellulose/vitamin C as a new biopolymer catalyst for synthesis of pharmacologically active 1-(α-aminoalkyl)naphthols 145*via* a one-pot condensation of 2-naphthol, alkylamines and aldehydes at room temperature within 20–55 h under solvent-free conditions in 80–95% yields (Scheme 102).

**Scheme 103:**
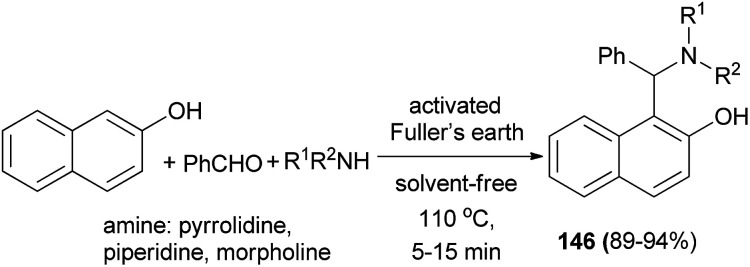
Activated Fuller's earth-catalyzed synthesis of 1-(amido/amino)alkyl-2-naphthols 146.

Chaturbhuj *et al.*^112^ succeeded in preparing 1-(amino)alkyl-2-naphthols 146*via* one-pot multi-component reaction of benzaldehyde, 2-naphthol and cyclic amines using activated Fuller's earth as a heterogeneous catalyst under solvent-free condition at 110 °C within 5–15 min in 89–94% yields (Scheme 103).

**Scheme 104:**
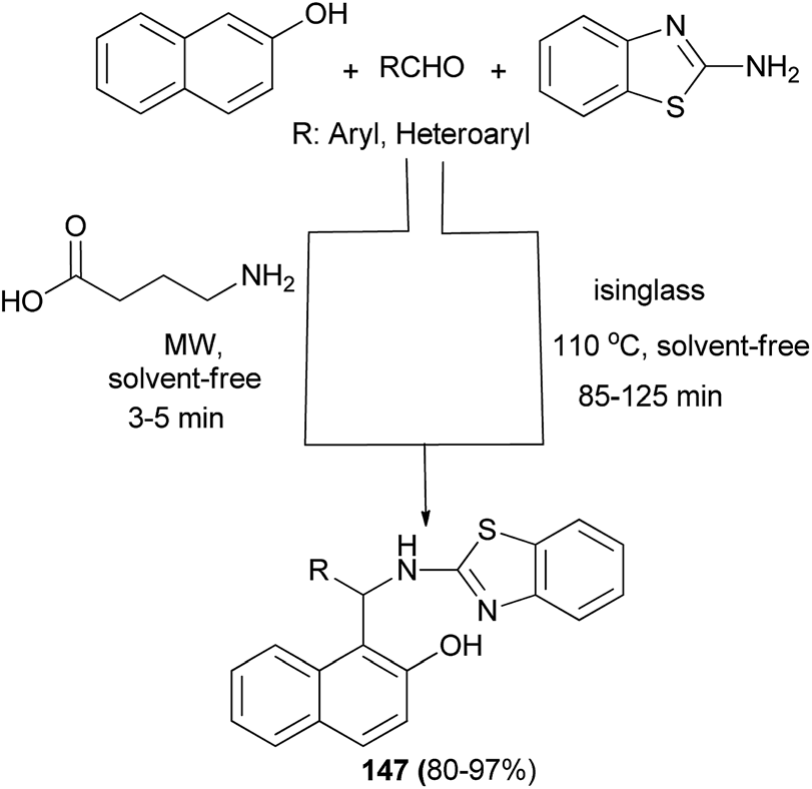
Synthesis of compounds 147.

γ-Aminobutyric acid (GABA) and isinglass, a collagen peptide, have been utilized as highly efficient bifunctional biocatalysts for the efficient and convenient synthesis of 2-aminobenzothiazolomethyl-2-naphthols through a one-pot three-component Mannich reaction between diverse aldehydes, 2-naphthol and 2-aminobenzothiazole under solvent-free condition in high yields. The reaction in the presence of GABA (10 mol%) was carried out under a microwave irradiation power of 900 W for 3–5 min and with isinglass at 110 °C for 85–125 min to afford the desired products 147 in 87–97% and 80–95% yields, respectively (Scheme 104).^113^

**Scheme 105:**
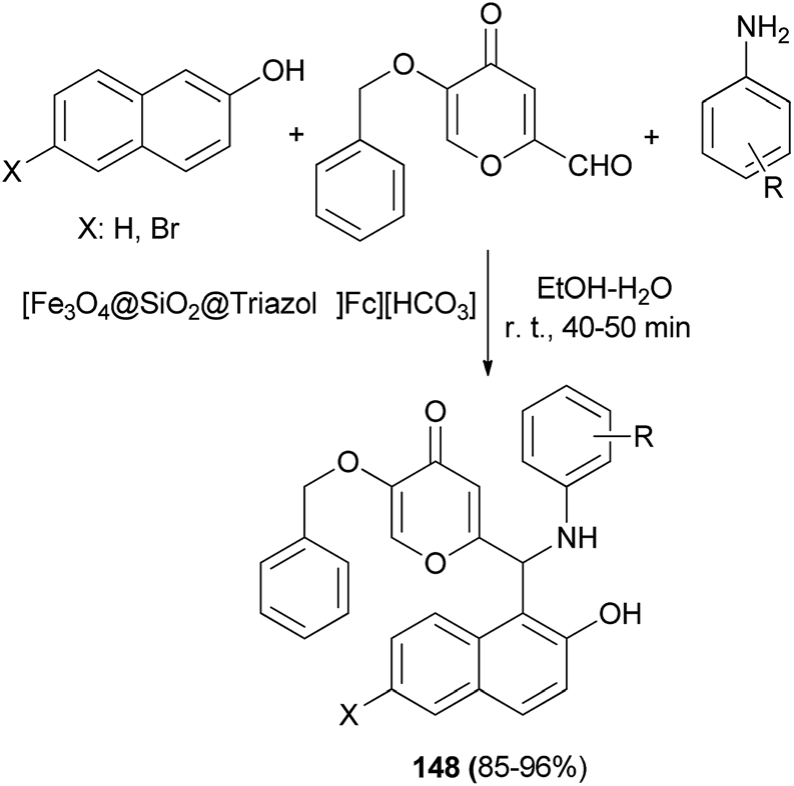
Synthesis of Betti bases 148 using [Fe_3_O_4_@SiO_2_@Triazol-Fc][HCO_3_] nanocatalyst.

Application of novel 1,2,3-triazolylferrocene-containing ionic liquid supported on Fe_3_O_4_ nanocatalyst in the synthesis of new pyran-substituted Betti bases 148 has been reported by Safa *et al.*^114^ The catalytic activity of the magnetic nanoparticles was evaluated in the one-pot three-component synthesis of a wide variety of Betti bases 148 in 85–96% yields from kojic aldehyde, 2-naphthol or 6-bromo-2-naphthol and aniline derivatives in EtOH–H_2_O at room temperature for 40–50 min (Scheme 105).

**Scheme 106:**
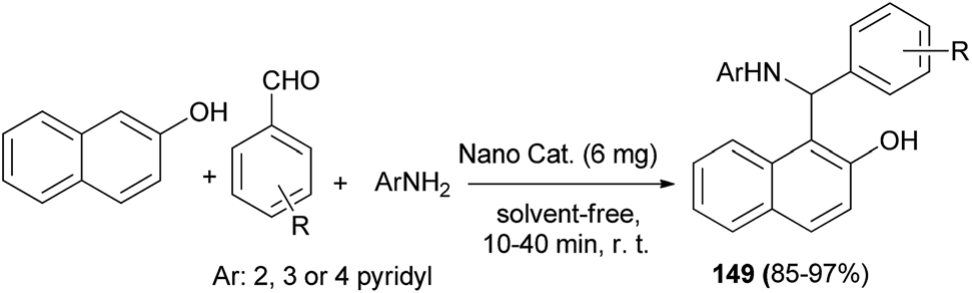
Synthesis of 1-(α-aminoalkyl)-2-naphthols 149 with Fe_3_O_4_@SiO_2_@MoSB.

Synthesis of 1-(α-aminoalkyl)-2-naphthol derivatives 149 employing combined ultrasonic/Mo Schiff base complex immobilized on Fe_3_O_4_ nanoparticles (Fe_3_O_4_@SiO_2_@MoSB) as a heterogeneous acid catalyst has been reported *via* the reaction of 2-naphthol, aromatic aldehydes and 2-, 3- or 4-aminopyridine under ultrasound irradiation without solvent at room temperature. All the reactions proceeded equally smoothly and afforded the desired products in 85–97% yields and short reaction time within 10–40 min irrespective of the nature of the substituents (Scheme 106). Additionally, binding interactions of 1-(phenyl(pyridin-2-ylamino)methyl)naphthalen-2-ol with various types of rigid DNA and HSA have been investigated by molecular modeling study. *In vitro* studies under physiological conditions showed that the desired derivative interacts with calf-thymus DNA *via* an intercalative binding mode.^115^

**Scheme 107:**
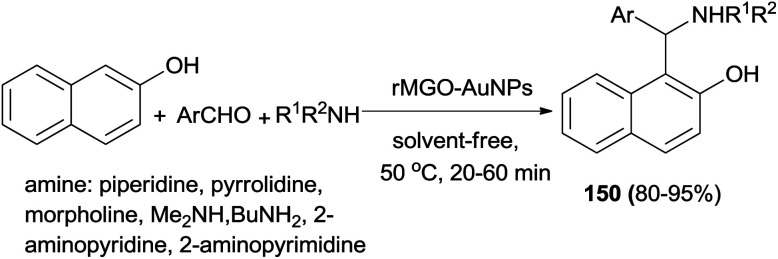
Preparation of 1-(α-aminoalkyl)naphthols 150 using rMGO–AuNPs.

Fathalipour *et al.*^116^ studied aqueous suspension of biocompatible reduced graphene oxide–AuNPs composite (rMGO–AuNPs) as an effective recyclable catalyst in a Betti reaction. In this process, 1-(α-aminoalkyl)naphthols 150 were obtained at 50 °C under solvent-free conditions after 20–60 min in 80–95% yields (Scheme 107).

## 4. Synthesis of bis-Betti base derivatives and synthetic applications

**Scheme 108:**
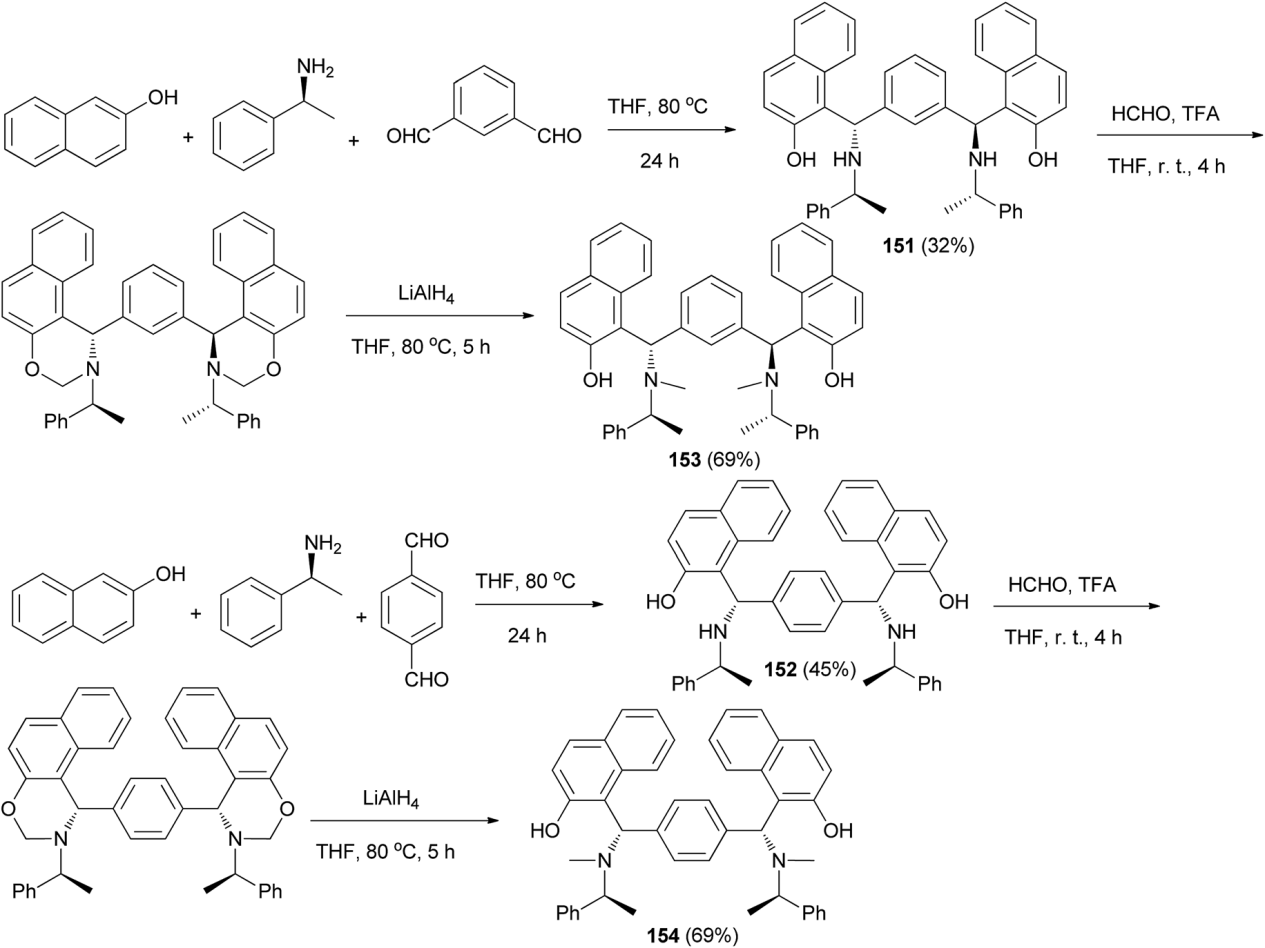
Synthesis of bis-Betti bases 151–154.

Among Betti bases, the bis derivatives are less studied. Zhang *et al.*^117^ reported new kinds of chiral alkylaminobenzylnaphthols (bis-Betti bases) 151 and 152 enantioselectively synthesized for the first time *via* the reaction of methylbenzylamine, dialdehydes and 2-naphthol in THF at 80 °C for 24 h in 32 and 45% yields, respectively. Subsequently, treatment of bis-Betti bases 151 and 152 with formaldehyde in the presence of TFA in THF at room temperature for 4 h followed by reduction with LiAlH_4_ within 5 h in THF at 80 °C afforded bis-Betti bases 153 and 154 in 60 and 69% yields, respectively (Scheme 108).

**Scheme 109:**
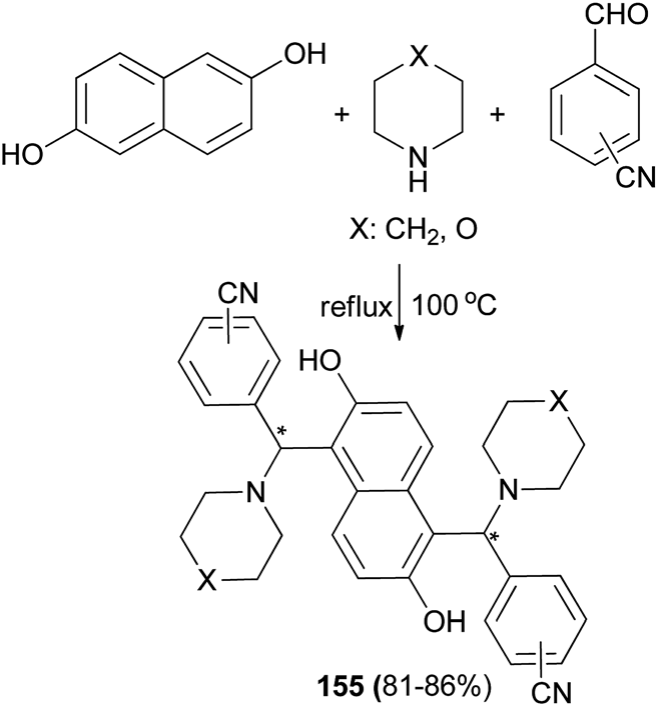
One-pot preparation of bis-Betti bases 155.

Xiong^118^ described a short and facile synthesis of bis-Betti bases 155 with two chiral carbon centers *via* the reaction of 2,6-dihydroxynaphthalene, piperidine or morpholine and cyanobenzaldehyde at 100 °C under reflux condition, affording the anticipated products in 81–86% yields (Scheme 109). This reaction has two steps. Firstly, the reaction of 2,6-dihydroxynaphthalene with cyanobenzaldehyde and piperidine or morpholine affords mono-Betti base products. And then, mono-Betti base product is used as 2-naphthalene continuously to react with excess cyanobenzaldehyde and piperidine or morpholine to give the final bis-Betti base products.

**Scheme 110:**
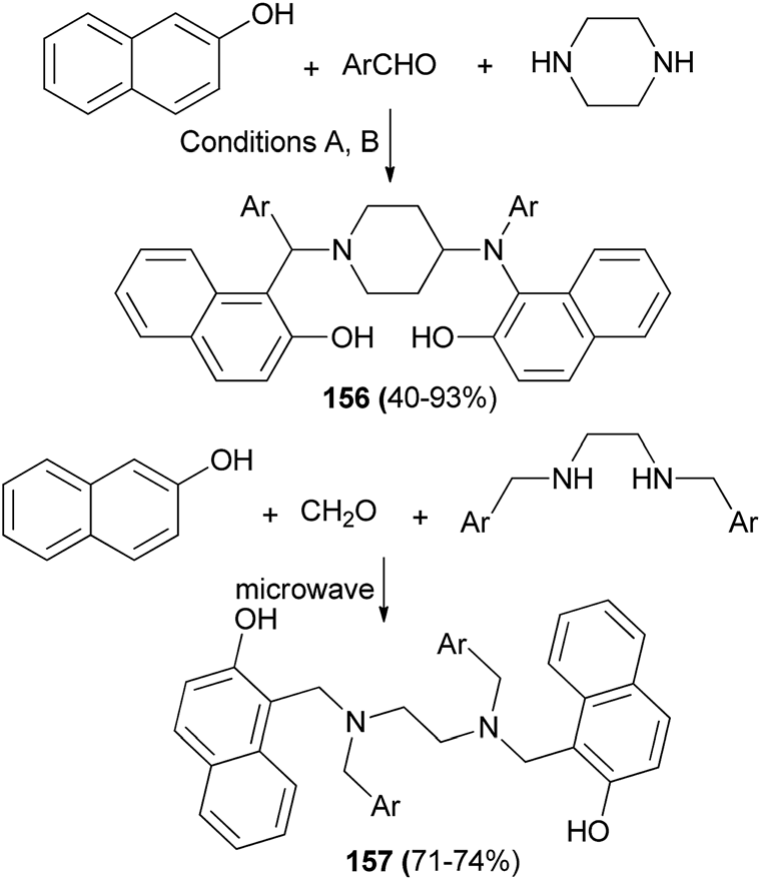
Synthesis of bis-Mannich bases 156 and 157.

Microwave-assisted synthesis of bis-Mannich bases 156 and 157 of 2-naphthols derived from aromatic aldehydes and diamines namely piperazine and *N*,*N*′-dialkylethylenediamines was studied under two conditions, as follows: (A) solvent-free microwave irradiation using a CEM Discover S Class microwave oven at 125 °C for five minutes in the absence of any catalyst; (B) reflux in ethanol for 72 h in the presence of catalytic amount of *p*TSA (Scheme 110).^119^

**Scheme 111:**
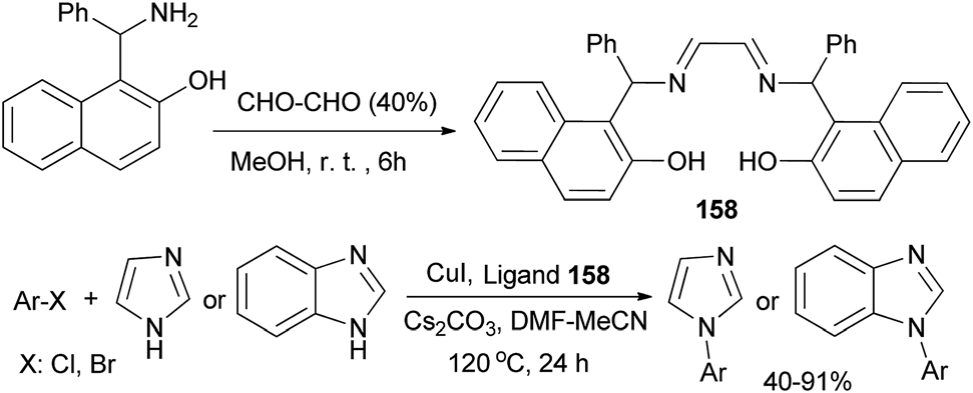
Synthesis of bis-Betti base 158 and application in copper-catalyzed *N*-arylation of imidazoles.

Weng and Li^120^ disclosed that bis-Betti base-derived tetradentate ligand 158 could be synthesized by the reaction of Betti base with glyoxal in MeOH at room temperature for 6 h. The combination of CuI, Cs_2_CO_3_, DMF–MeCN and the proper amount of 158 was a more efficient system to catalyze the *N*-arylation of imidazoles at 120 °C within 24 h. To explore the scope of this catalytic system, a variety of aryl bromides and aryl chlorides were examined, and the corresponding products in 40–91% yields were obtained (Scheme 111).

**Scheme 112:**
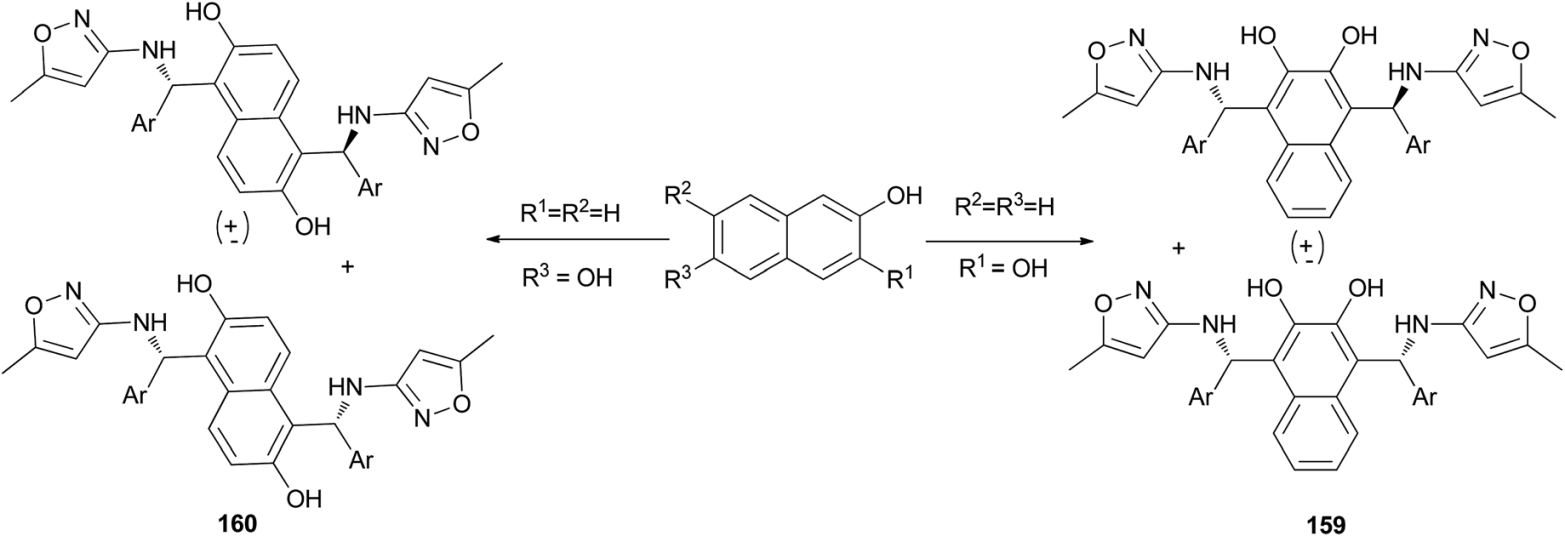
Synthesis of bis-Betti bases 159 and 160 under solvent-free conditions.

An efficient, expeditious, and diastereoselective one-pot pseudo-five-component reaction for the synthesis of bis-Betti bases 159 and 160 has been reported. The reaction of 2,3-dihydroxynaphthalene or 2,6-dihydroxynaphthalene, two equivalents of arylaldehydes, and two equivalents of 3-amino-5-methylisoxazole was carried out at 80 °C under solvent-free conditions within 0.5–6 h, affording the corresponding products in 70–95% yields (Scheme 112).^121^

**Scheme 113:**
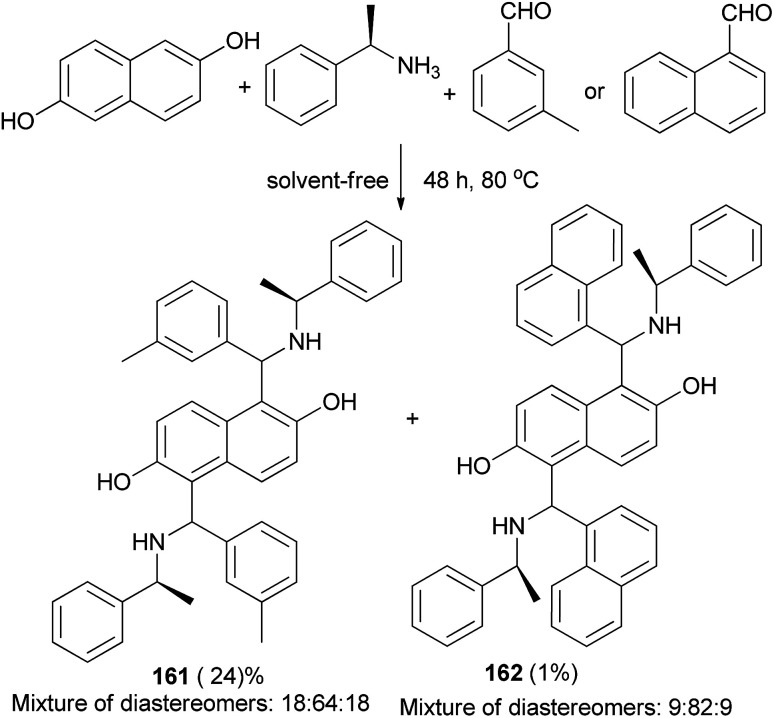
Diastereoselective synthesis of bis-Betti bases 161 and 162.

Bis-Betti bases 161 and 162 have been synthesized diastereoselectively by applying a solvent-free ‘Betti-type’ condensation using 2,6-dihydroxynaphthalene, (*S*)-phenylethylamine, and *m*-methylbenzaldehyde or 1-naphthaldehyde at 80 °C for 48 h. The major diastereomers formed could be isolated in pure form (Scheme 113).^73^

**Scheme 114:**
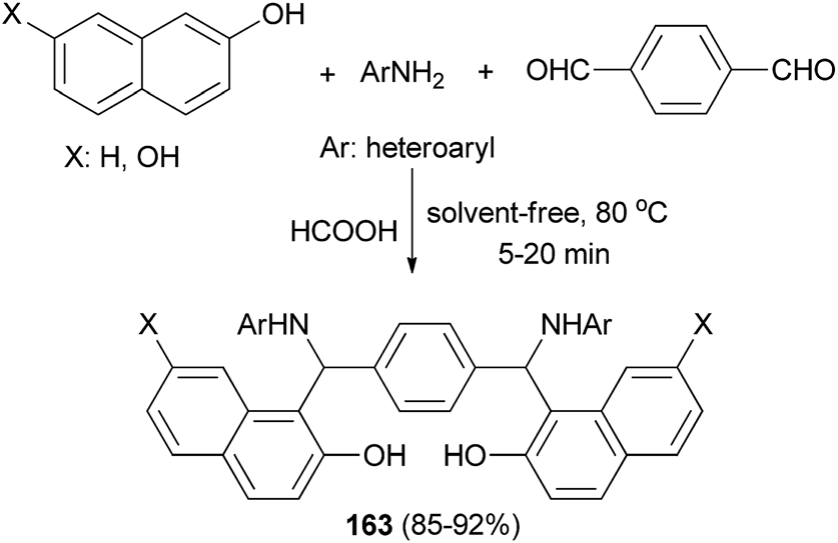
Synthesis of bis-Betti base derivatives 163.

A mild, efficient and straightforward method has been developed for the synthesis of bis-Betti bases 163*via* a pseudo-five-component, one-pot condensation reaction of heteroarylamines, terephthaldehyde and naphthols in the presence of formic acid as catalyst at 80 °C within 5–20 min under solvent-free conditions (Scheme 114). A wide range of heteroarylamines with electron-donating and electron-withdrawing groups and naphthols were well tolerated under the reaction conditions and afforded products in 85–92% yields.^122^

**Scheme 115:**
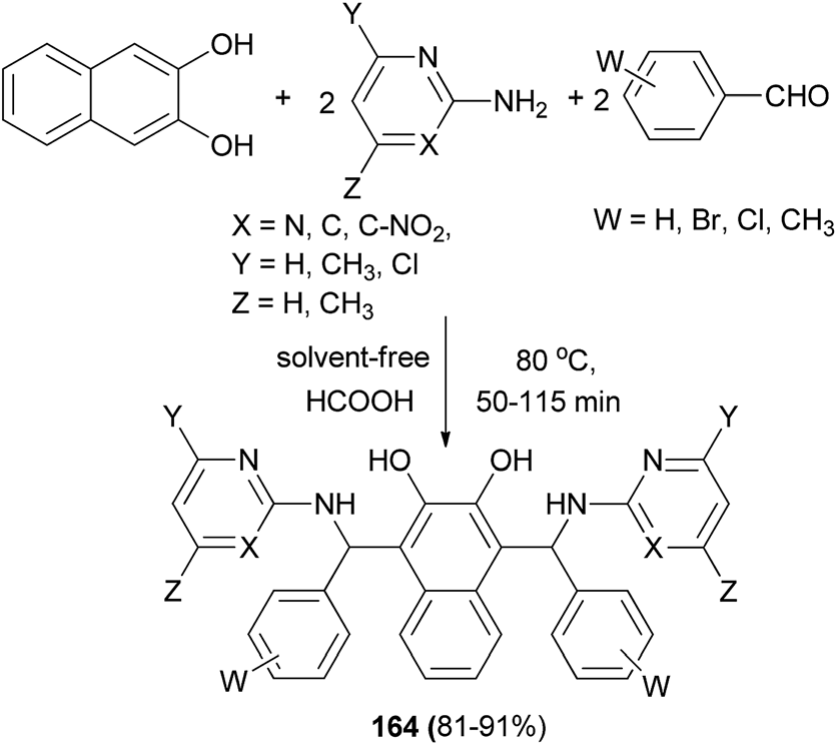
One-pot synthesis of bis-Betti bases 164.

A simple and efficient procedure for the synthesis of bis-Betti bases 164*via* a one-pot pseudo-five-component reaction of one equivalent of 2,3-dihydroxynaphthalene, two equivalents of arylaldehydes, and two equivalents of heteroarylamines in the presence of formic acid catalyst at 80 °C for 50–115 min under solvent-free conditions has been described. This reaction worked well with heteroaromatic amines such as 2-aminopyrimidines and 2-aminopyridine derivatives and afforded desired products in 81–91% yields (Scheme 115).^123^

**Scheme 116:**
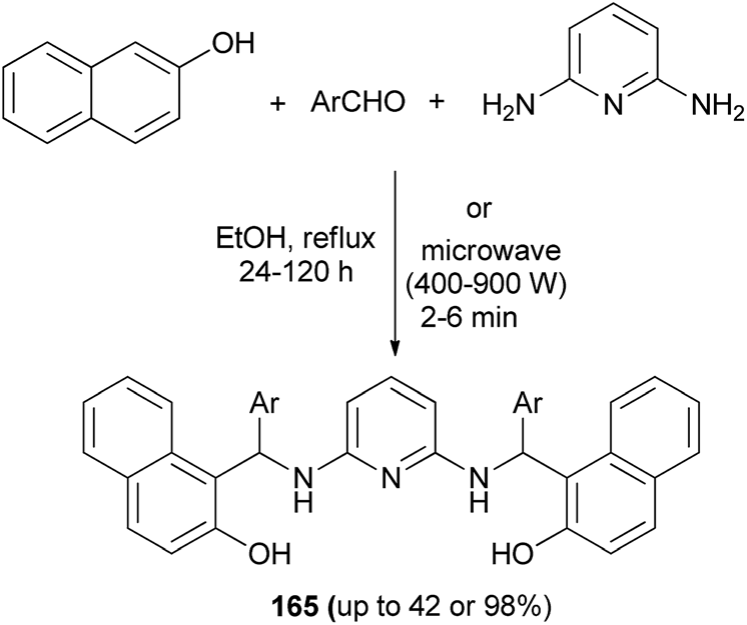
Synthesis of bis-Betti bases 165.

Abdul and Hussain^124^ have exploited an efficient method for the synthesis of *N*,*N*′-bis-[(2-hydroxynapthalene-1-yl)(substituted phenyl)methyl]-2,6-diaminopyridine derivatives 165 by one-pot three-component reaction of 2-naphthol, 2,6-diaminopyridine and substituted aromatic aldehydes in ethanol at 80 °C within 24–120 h and under microwave irradiation within 2–6 min at high power (400–900 W) without any catalyst. The isolated yields in ethanol condition (up to 98%) are higher than in microwave-assisted solvent-free condition (up to 42%) (Scheme 116).

**Scheme 117:**
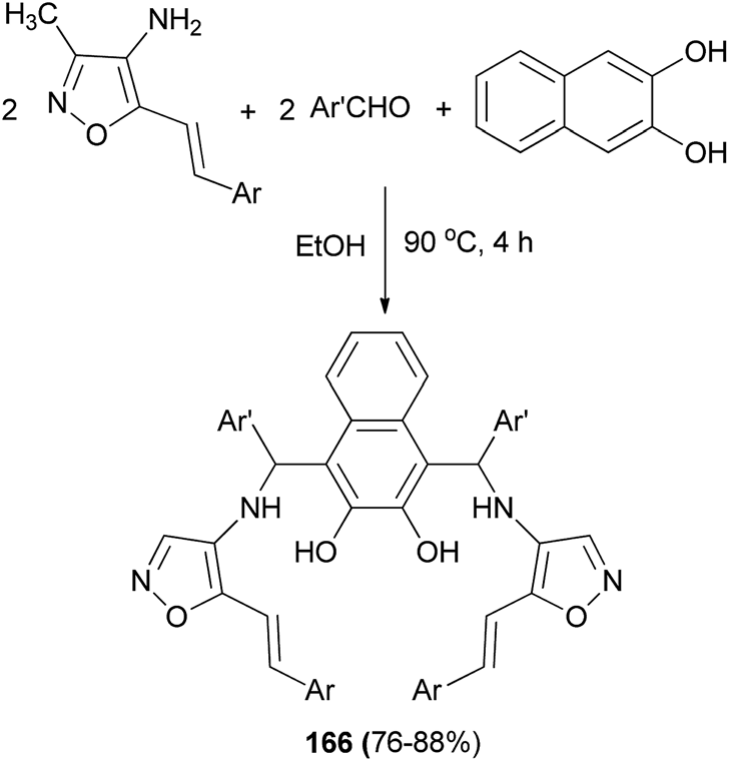
Synthesis of bis-Betti bases 166.

The reaction of 4-amino-3-methyl-5-styrilisooxazole, aromatic aldehydes and 2,3-dihydroxynaphthalene in ethanol at 90 °C for 4 h afforded bis-Betti bases 166. In all cases, the products were obtained in 76–88% yields (Scheme 117).^125^

## 5. Conclusions

The high number of publications in the literature that have recently appeared on the syntheses of Betti bases and bis-Betti base derivatives with different methods using various types of naphthols, amines and aldehydes in various conditions indicates the importance of these compounds. These procedures are commonly classified as Mannich aminoalkylations. The non-racemic nature of the Betti base and derivatives could be of special interest for organic chemists working in the field of ligand–metal catalyzed reactions. It is also clear that the most important area of application of the non-racemic aminonaphthols is their use in asymmetric transformations, either as chiral ligands or as chiral auxiliaries. Investigation of Betti base structures indicated that some of these products possess biological activity. However, in spite of the number of structures reported, it is possible to synthesize and investigate applications of the various structures of these compounds due to their importance.

## Conflicts of interest

There are no conflicts to declare.

## Supplementary Material
