# Correction: Recent advances in the synthesis and synthetic applications of Betti base (aminoalkylnaphthol) and bis-Betti base derivatives

**DOI:** 10.1039/d0ra90013c

**Published:** 2020-02-14

**Authors:** Abolfazl Olyaei, Mahdieh Sadeghpour

**Affiliations:** Department of Chemistry, Payame Noor University (PNU) PO Box 19395-4697 Tehran Iran olyaei_a@pnu.ac.ir; Department of Chemistry, Takestan Branch, Islamic Azad University Takestan Iran

## Abstract

Correction for ‘Recent advances in the synthesis and synthetic applications of Betti base (aminoalkylnaphthol) and bis-Betti base derivatives’ by Olyaei *et al.*, *RSC Adv.*, 2019, **9**, 18467–18497.

The authors apologise that two related references were not cited in the original article. Ref. 2 and 4 should be updated to include the two new references as ref. 2*b* and 4*b*. The authors also apologise for unattributed text overlap in the Introduction and Section 2 with ref. 2*b* and 4*b*.

Citations to the work of Cardellicchio *et al.* (ref. 2*b*)^1^ and Naso (ref. 4*b*)^2^ should have been included at the following locations in the original article.

On page 18467, a citation to ref. 4*b* should be added to the end of the sentences beginning “In 1897,…” and “In 1898,…”. Therefore, the sentences should be changed to “In 1897, Betti obtained his degree with a thesis on the reaction of methylisoxazolones with aldehydes and co-authored with Roberto Schiff the first two papers of his career.^3,4*a*,*b*^ In 1898, Betti moved to the University of Florence as an assistant of Ugo Schiff, who was founder and director of the Institute of Chemistry.^4*b*^”

On page 18648, the sentence beginning “Noyori considered him…” should be changed to “Noyori considered him to be the real pioneer of asymmetric synthesis,^6^ since Betti reacted methylmagnesium iodide and benzaldehyde in the presence of *N*,*N*-dimethylbornylamine.^2*b*^”

On page 18648, a citation should be added to ref. 4*b* at the end of the second paragraph, therefore the final sentence beginning “Finally, the base…” should be changed to “Finally, the base was also easily resolved into its optical isomers by means of tartaric acid.^4*b*,9^”

On page 18648, a citation should be added to ref. 2*b* at the end of the final sentence of Section 2. The sentence beginning “Addition of a…” should be changed to “Addition of a solution of sodium hydroxide to chloride yielded Betti base **1** (75% yield).^2*b*,7,13^”

The authors also regret their oversight in omitting to acknowledge the source of the image in Fig. 1. The corrected caption for Fig. 1 is shown below.

Fig. 1: Italian Senator Mario Betti. This image was reproduced from https://www.liberliber.it/online/autori/autori-b/mario-betti. A version of this image was also originally published in *Substantia: An International Journal of the History of Chemistry*, 2017, **1**(2), 111–121.

The Royal Society of Chemistry apologises for these errors and any consequent inconvenience to authors and readers.

## Supplementary Material
